# Multi-redox indenofluorene chromophores incorporating dithiafulvene donor and ene/enediyne acceptor units

**DOI:** 10.3762/bjoc.20.8

**Published:** 2024-01-15

**Authors:** Christina Schøttler, Kasper Lund-Rasmussen, Line Broløs, Philip Vinterberg, Ema Bazikova, Viktor B R Pedersen, Mogens Brøndsted Nielsen

**Affiliations:** 1 Department of Chemistry, University of Copenhagen, Universitetsparken 5 DK-2100 Copenhagen Ø, Denmark; 2 Sino-Danish College (SDC), University of Chinese Academy of Sciences, Beijing, China

**Keywords:** alkynes, chromophores, fused-ring systems, heterocycles, redox chemistry

## Abstract

Large donor–acceptor scaffolds derived from polycyclic aromatic hydrocarbons (PAHs) with tunable HOMO and LUMO energies are important for several applications, such as organic photovoltaics. Here, we present a large selection of PAHs based on central indenofluorene (IF) or fluorene cores and containing various dithiafulvene (DTF) donor units that gain aromaticity upon oxidation and a variety of acceptor units, such as vinylic diesters, enediynes, and cross-conjugated radiaannulenes (RAs) that gain aromaticity upon reduction. In some cases, the DTF units are expanded by pyrrolo annelation. The optical and redox properties of these compounds, in some cases carbon-rich, were studied by UV–vis absorption spectroscopy and cyclic voltammetry. Synthetically, the work explores IF diones or fluorenone as central building blocks by subjecting the carbonyl groups to a variety of reactions; that are, phosphite- or Lawesson’s reagent-mediated olefination reactions (to introduce DTF motifs), Ramirez/Corey–Fuchs dibromo-olefinations followed by Sonogashira couplings (to introduce enediynes motifs), and Knoevenagel condensations (to introduce the vinylic diester motif). By a subsequent Glaser–Hay coupling reaction, a RA acceptor unit was introduced to provide a DTF-IF-RA donor–acceptor scaffold with a low-energy charge-transfer absorption and multi-redox behavior.

## Introduction

**Figure 1:**
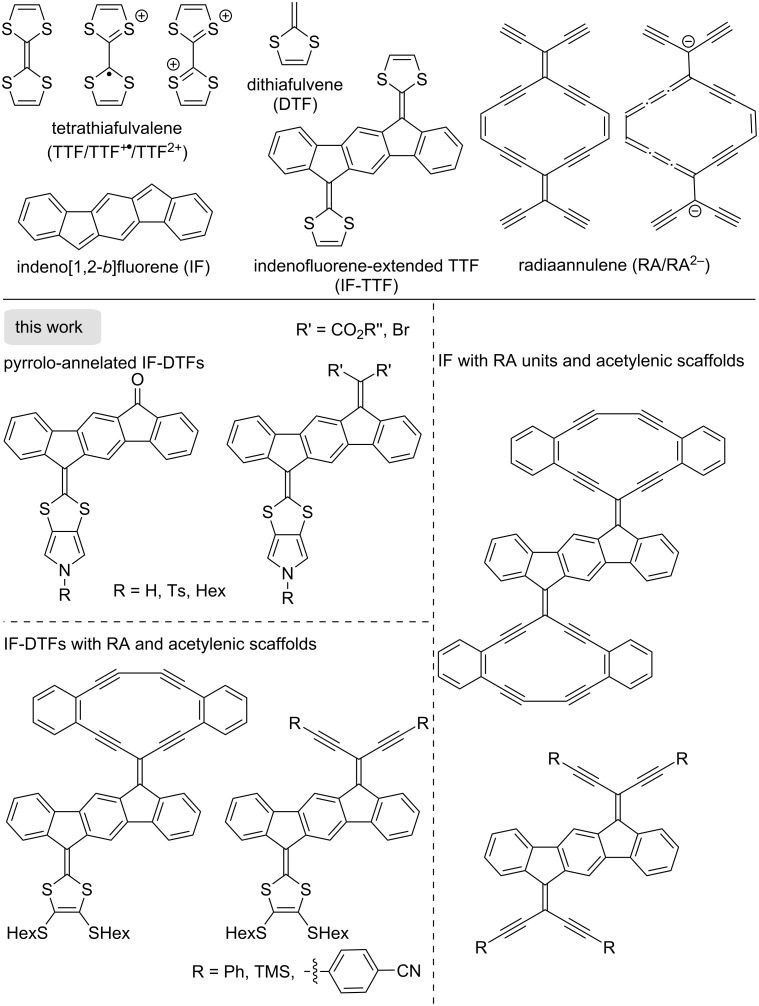
Overview of structural motifs relevant for the work described herein.

Tetrathiafulvalene (TTF, Figure 1) is a redox-active molecule that has been widely explored in materials chemistry and supramolecular chemistry [1–8]. TTF reversibly undergoes two sequential one-electron oxidations, generating first a radical cation (TTF^+•^) and subsequently a dication (TTF^2+^) containing two 6π-aromatic 1,3-dithiolium rings. The redox properties and geometries of the redox states have been finely tuned by extending the conjugated system with various cores, such as polycyclic aromatic hydrocarbons (PAHs), resulting in so-called extended TTFs [9–12]. One example of this is the introduction of an indeno[1,2-*b*]fluorene (IF) core [13], providing indenofluorene-extended TTFs (IF-TTFs) of the general structure shown in Figure 1. The π-system can be further expanded as well at the dithiole rings. For example, we have recently developed a synthetic protocol for fusing a pyrrole unit to one of the dithiole rings of an IF-TTF, allowing for dimerization of extended TTFs via the nitrogen atom by different linkers [14].

Donor–acceptor chromophores can be obtained by replacing one of the dithiafulvene (DTF) rings of the IF-TTF by an electron acceptor. Cyclic and acyclic acetylenic scaffolds comprised of enediyne units are known to behave as good electron acceptors [15–16], and we became interested in combining the IF-DTF scaffold with such motifs to generate novel multi-redox systems. For example, the radiaannulene moiety RA shown in Figure 1 (or its truncated counterpart with one of the exocyclic enediyne units removed) [17–18] is a particularly good electron acceptor as it gains 14π-aromaticity upon reduction. In this work, we also want to further explore pyrrolo-annelated IF-DTFs with different substituents on the nitrogen atom, and the functionalization at the other end of the IF core with electron-accepting moieties. An overview of general motifs targeted in this work is shown in Figure 1.

## Results and Discussion

### Synthesis

**Figure 2:**
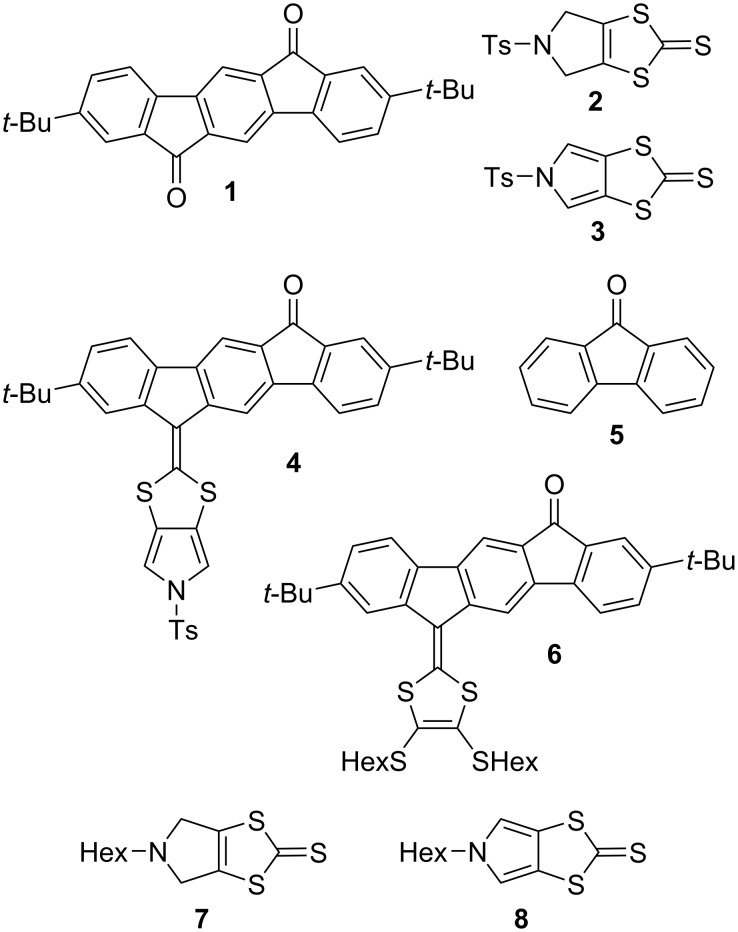
Dione/ketones **1**, **4**–**6** and 1,3-dithiole-2-thione compounds **2**, **3**, **7**, and **8** are building blocks used in this work.

The synthetic building blocks **1**–**8** used in this work are shown in Figure 2. The dione **1** and the ketones **4** and **6** were synthesized according to literature procedures [14,19–20], as were the 1,3-dithiole-2-thiones **2** and **3** [21]. Fluorenone **5** is commercially available. The new building blocks **7** and **8** were prepared according to related literature procedures [21], as described in Supporting Information File 1.

**Scheme 1:**
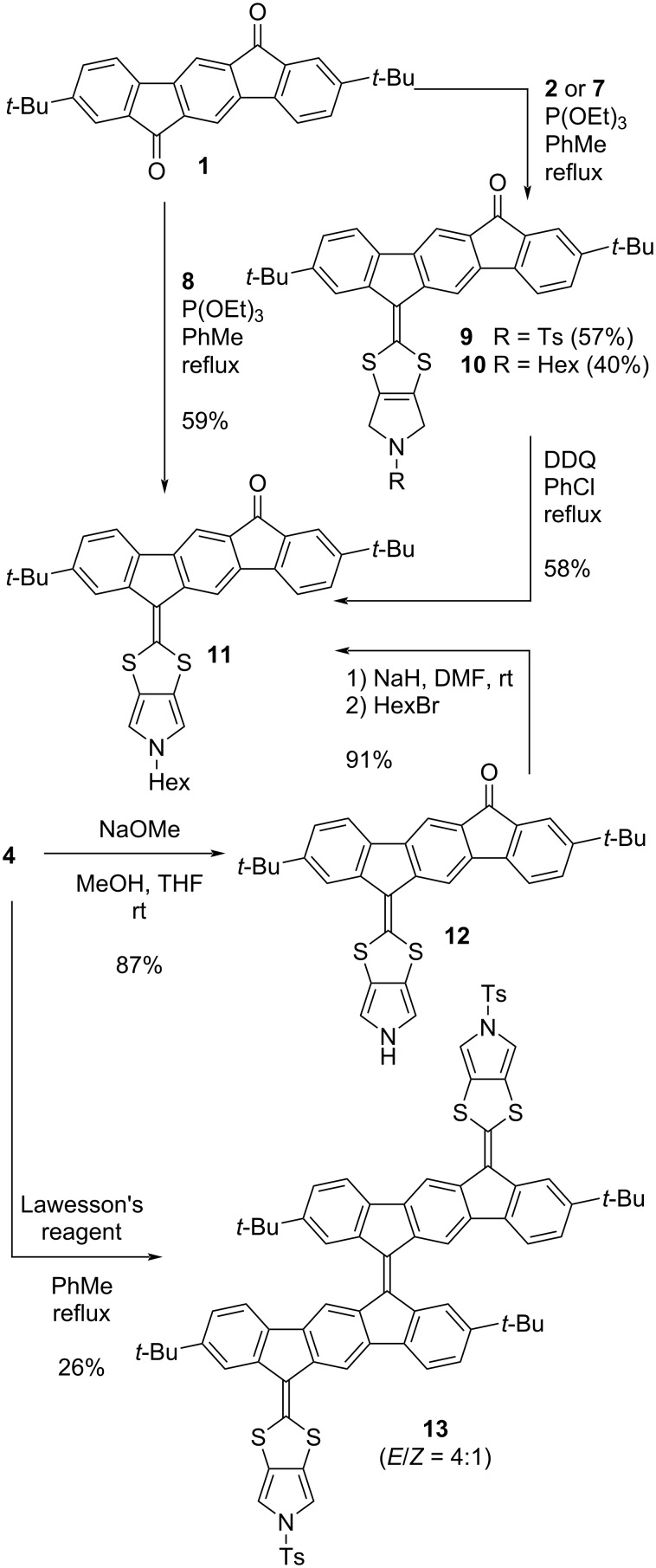
Synthesis of IF-DTF ketones **9–12** and dimer **13**.

**Scheme 2:**
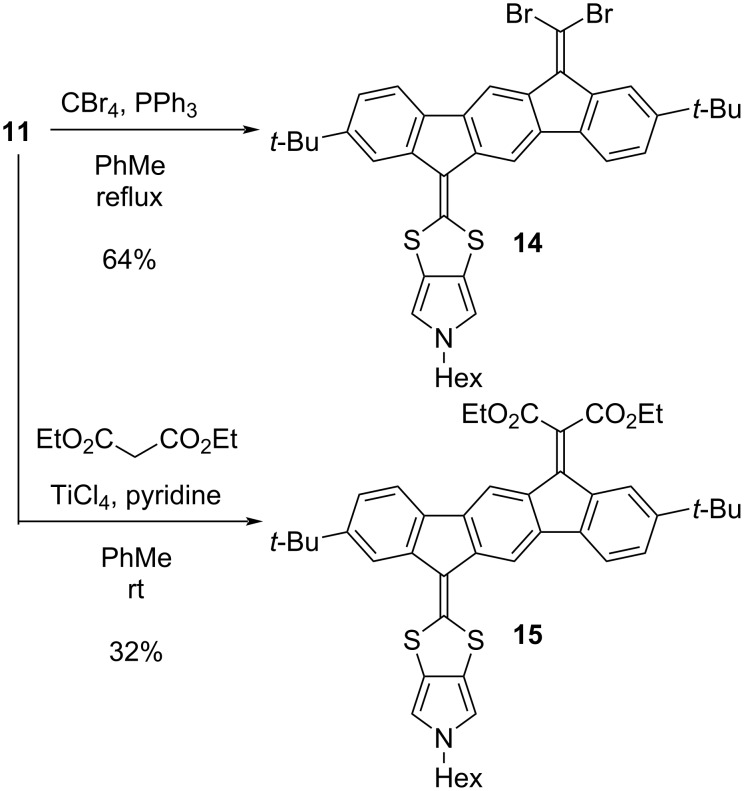
Further functionalization of the IF-DTF ketone **11** via Ramirez/Corey–Fuchs dibromo-olefination and Knoevenagel condensation.

Our first objective was to explore further annellation of dihydropyrrole and pyrrole units at the DTF moiety of an IF-DTF. A phosphite-mediated coupling of either 1,3-dithiole-2-thione **2**, **7**, or **8** with IF dione **1** afforded IF-DTFs **9**–**11**, as shown in Scheme 1. Compound **11** was also obtained from building block **4** via the pyrrolo-annelated IF-DTF **12** by removal of the tosyl (Ts) group under alkaline conditions, followed by nucleophilic substitution to incorporate the hexyl chain on the pyrrole. Furthermore, treatment of the IF-DTF ketone **4** with Lawesson’s reagent (using a recently established protocol [20]) yielded the large dimer **13** as a mixture of *E* and *Z* isomers (ca. 4:1). Further functionalization of the IF-DTF ketone **11** was obtained by Ramirez/Corey–Fuchs dibromo-olefination and Knoevenagel condensation to yield vinylic dibromide **14** and diester **15**, respectively, as illustrated in Scheme 2. We noted that the dibromo-olefination reaction was first discovered by Ramirez and co-workers [22] and used in the first step of the Corey–Fuchs reaction that ultimately provides an alkyne [23].

**Scheme 3:**
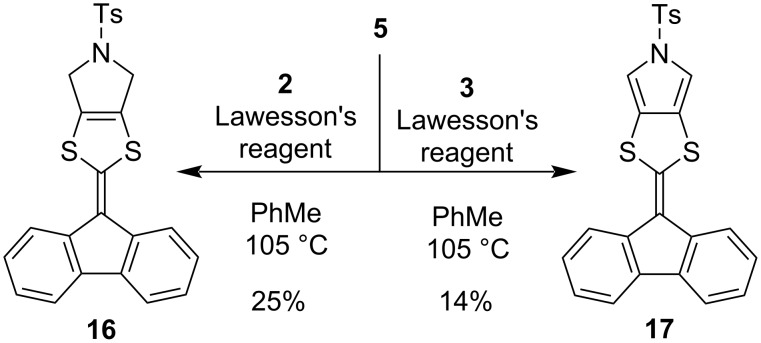
Coupling of 1,3-dithiole-2-thione building blocks **2** and **3** with fluorenone **5** to afford fluorene-extended DTFs **16** and **17**.

To elucidate the properties of the donor part itself of the pyrrolo-annelated IF-DTF systems, we prepared compounds **16** and **17** containing a smaller fluorene PAH. These compounds were prepared by a Lawesson’s reagent-promoted coupling between fluorenone **5** and the Ts-protected 1,3-dithiole-2-thione building blocks **2** and **3**, respectively, shown in Scheme 3 (albeit in modest yields). Fluorene-based DTF compounds have previously been explored in various elaborate systems [24–27].

**Scheme 4:**
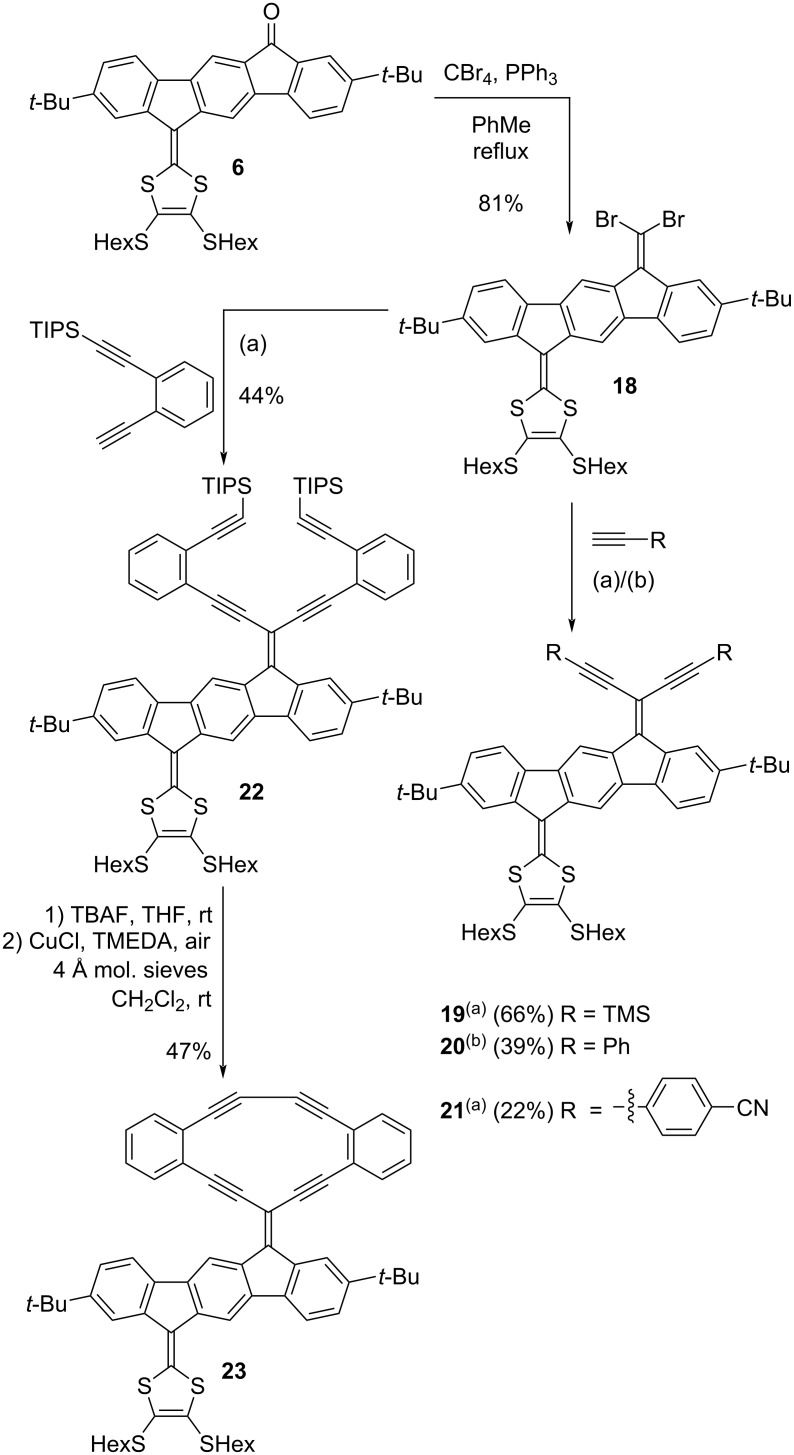
Synthesis of acetylenic scaffolds based on IF-DTF. Conditions: (a) Pd(PPh_3_)_2_Cl_2_, CuI, THF, Et_3_N, rt. (b) Pd_2_dba_2_, P(*t*-Bu)_3_, CuI, THF, Et_3_N, rt.

Next, we wanted to explore IF-DTFs as motifs for acetylenic scaffolding (Scheme 4). Starting from IF-DTF building block **6**, dibromo-olefinated compound **18** was obtained by a Ramirez/Corey–Fuchs reaction. Two-fold Sonogashira couplings with trimethylsilylacetylene, ethynylbenzene, or 4-ethynylbenzonitrile yielded compounds **19**–**21**, while two-fold Sonogashira coupling with ((2-ethynylphenyl)ethynyl)triisopropylsilane resulted in compound **22**. Desilylation of the alkynes of compound **22** with tetrabutylammonium fluoride (TBAF) and subsequent intramolecular Glaser–Hay coupling of the terminal alkynes afforded the macrocyclic DTF-IF-RA scaffold **23**. Molecular sieves (4 Å) were added to the reaction mixture as this has previously been shown to significantly promote the Glaser–Hay coupling [28]. Compounds **20** and **21** were unfortunately very sensitive compounds that were found to easily degrade, which made their characterization somewhat difficult (vide infra).

**Scheme 5:**
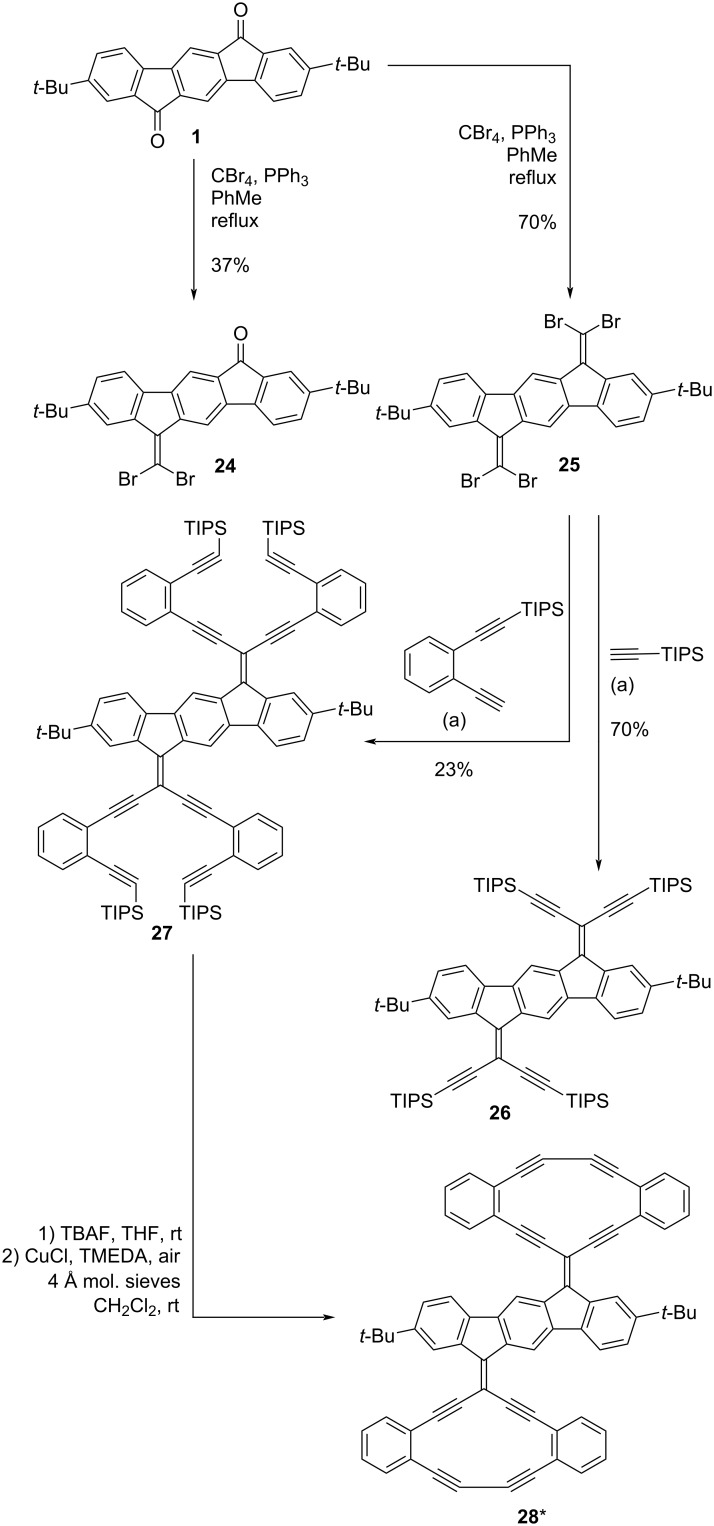
Synthesis of acetylenic scaffolds with IF as central core. *Not fully characterized due to poor solubility. Conditions: (a) Pd(PPh_3_)_2_Cl_2_, CuI, THF, Et_3_N, rt.

We also targeted other enediyne acetylenic scaffolds with IF as central core as shown in Scheme 5. Starting from IF dione **1**, compounds **24** and **25** were synthesized via Ramirez/Corey–Fuchs dibromo-olefinations. Four-fold Sonogashira couplings of compound **25** with triisopropylsilylacetylene and ((2-ethynylphenyl)ethynyl)triisopropylsilane yielded compounds **26** and **27**, respectively. A two-fold, intramolecular Glaser–Hay coupling of compound **27** (after desilylation) was attempted under the conditions that were successful in the synthesis of compound **23** (Scheme 4). A compound that may tentatively be assigned to **28** was observed by MALDI–MS analysis of the reaction mixture, but less than needed for an NMR sample was isolated. Furthermore, the isolated compound proved quite insoluble in all investigated deuterated solvents, and therefore it was not possible to determine the purity of the product by this method.

**Scheme 6:**
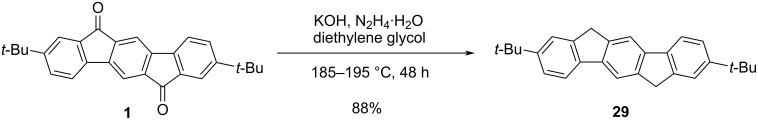
Reduction of IF dione **1** to dihydro-IF **29**.

In an initial attempt to investigate other synthetic pathways to extended IF compounds, the reduced IF **29** was synthesized from IF dione **1** by a Wolff–Kishner reduction of the two ketones as shown in Scheme 6. Compound **29** could potentially after deprotonation be reacted with electrophiles as previously established [29] for the parent structure [30] without *tert*-butyl substituents.

### UV–vis absorption spectroscopy

**Figure 3:**
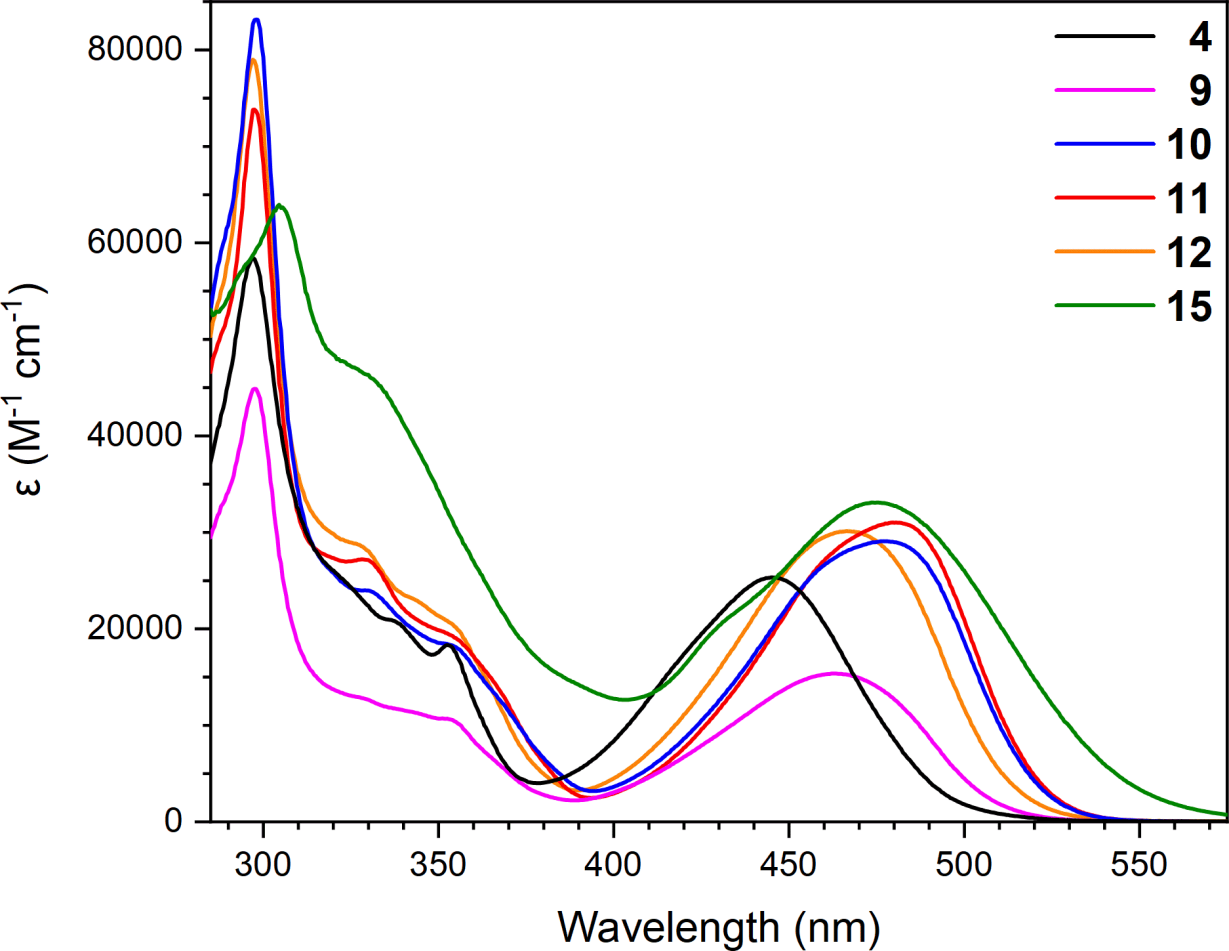
UV–vis absorption spectra of compounds **4**, **9**–**12**, and **15** in PhMe at 25 °C.

UV–vis absorption spectra of the known compound **4** [14] and new compounds **9**–**12** and **15** are depicted in Figure 3, and the data are presented in Table 1. A redshift of the longest-wavelength absorption maximum is observed for all new compounds compared to that of **4**. For compounds **11** and **12**, this indicates that the inductive electron-withdrawing or -donating influences of the substituent group (Ts group in **4** and Hex group in **11**) on the nitrogen atom in the pyrrole ring have an effect on the absorption in the visible spectrum of pyrrolo-annelated IF-DTF ketones. Interestingly, the absorption of the dihydropyrrole IF-DTF **9** is redshifted relative to that of the pyrrole IF-DTF **4**, while the absorption does not change significantly when comparing IF-DTFs **10** and **11**, indicating that the extent to which the absorption changes upon oxidation from a dihydropyrrole to a pyrrole unit depends on the substituent on the N of the dihydropyrrole/pyrrole ring. Introducing the diester electron-acceptor in compound **15** does not change the absorption significantly, compared to compound **11**. When changing the solvent from PhMe to CH_2_Cl_2_, we observed a redshift of the longest-wavelength absorption maximum for compounds **10** and **11**, indicating some charge-transfer character of the absorption (see Figure S1 in Supporting Information File 1).

**Table 1:** UV–vis absorption data of compounds in PhMe or CH_2_Cl_2_ at 25 °C (absorption maxima λ_max_ and molar absorptivities ε).

Compound	λ_max_ [nm] (ε [10^3^ M^−1^ cm^−1^])	Compound	λ_max_ [nm] (ε [10^3^ M^−1^ cm^−1^])

**4** ^a^	297 (58), 445 (25)	**16** ^b^	297 (3.6), 395 (21)*, 409 (22)
**9** ^a^	297 (45), 462 (20)	**17** ^b^	297 (7.1), 383 (27), 393 (25)*
**10** ^a^	298 (68), 478 (24)	**22** ^b^	262 (51), 300 (55), 402 (17) (broad), 489 (27)
**11** ^a^	298 (74), 480 (31)	**23** ^b^	297 (95), 401 (21)*, 426 (24), 444 (23)*, 529 (34)
**12** ^a^	297 (79), 466 (30)	**26** ^b^	296 (76), 413 (52), 440 (70)
**13** ^b,c^	269 (69), 312 (84), 574 (43)	**27** ^b^	306 (46), 444 (24)*, 461 (25), 534 (1.8) (broad)
**15** ^a^	304 (60), 475 (34)	**30** ^b,d^	251, 400*, 412

^a^PhMe; ^b^CH_2_Cl_2_; ^c^*E*/*Z* ratio of 4:1; ^d^reference [14]; *shoulder peak.

**Figure 4:**
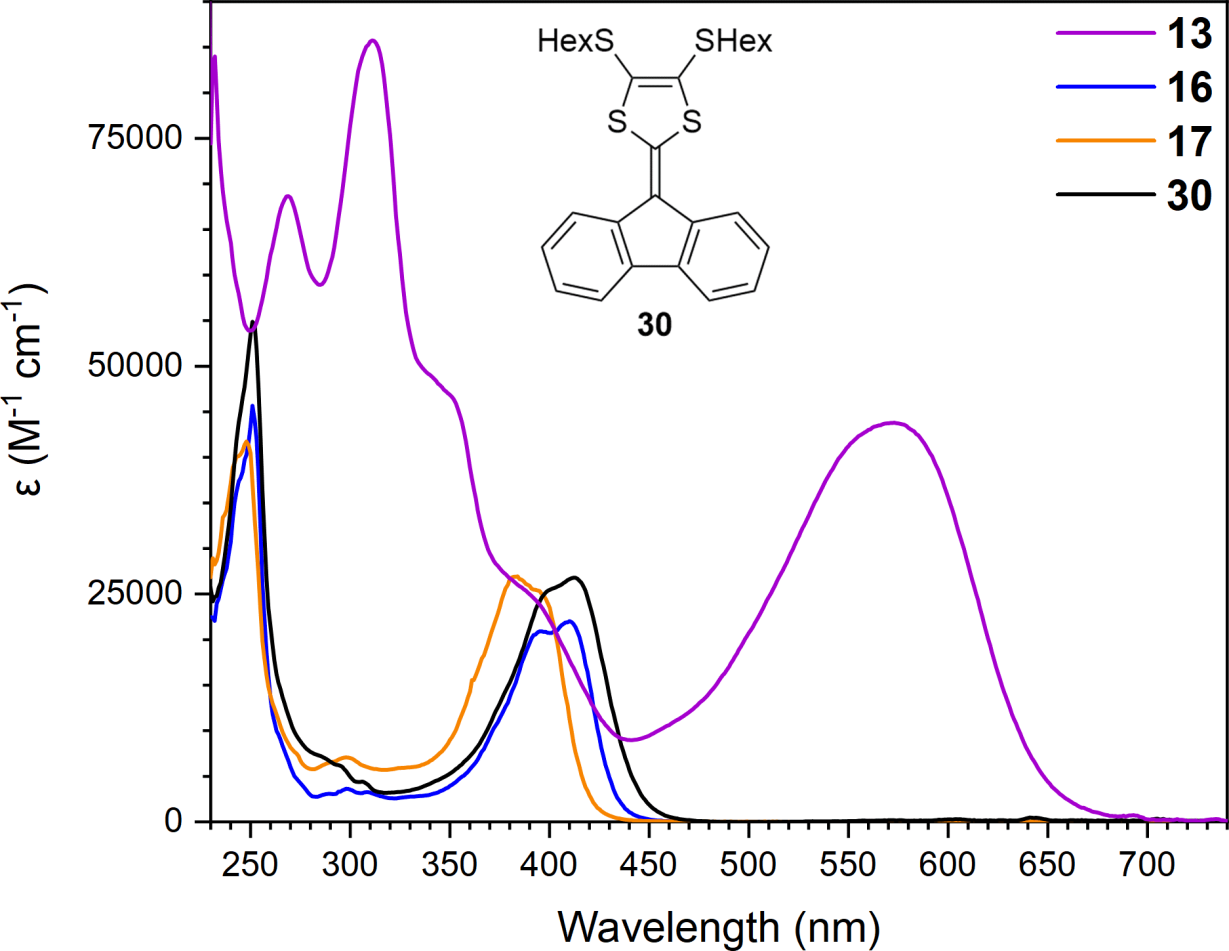
UV–vis absorption spectra of compounds **13**, **16**, **17**, and **30** in CH_2_Cl_2_ at 25 °C.

UV–vis absorption spectra of the known compound **30** [20] and new compounds **13**, **16**, and **17** are shown in Figure 4, and the data are presented in Table 1. Compared to compound **30**, the longest-wavelength absorption maximum of compound **16** is slightly blueshifted while the absorption maximum of compound **17** is significantly blueshifted. This indicates that annelation of the dihydropyrrole ring to the DTF moiety does not change the absorption maximum significantly compared to the two SHex substituents, while annelation of a pyrrole ring results in an absorption maximum at significantly shorter wavelength. These compounds have blueshifted longest-wavelength absorptions relative to the donor–acceptor scaffolds incorporating a pyrrolo-annelated DTF unit. Of these compounds, the large dimer **13** stands out with a significantly redshifted and intense longest-wavelength absorption maximum (λ_max_ at 574 nm) expanding to ca. 680 nm.

**Figure 5:**
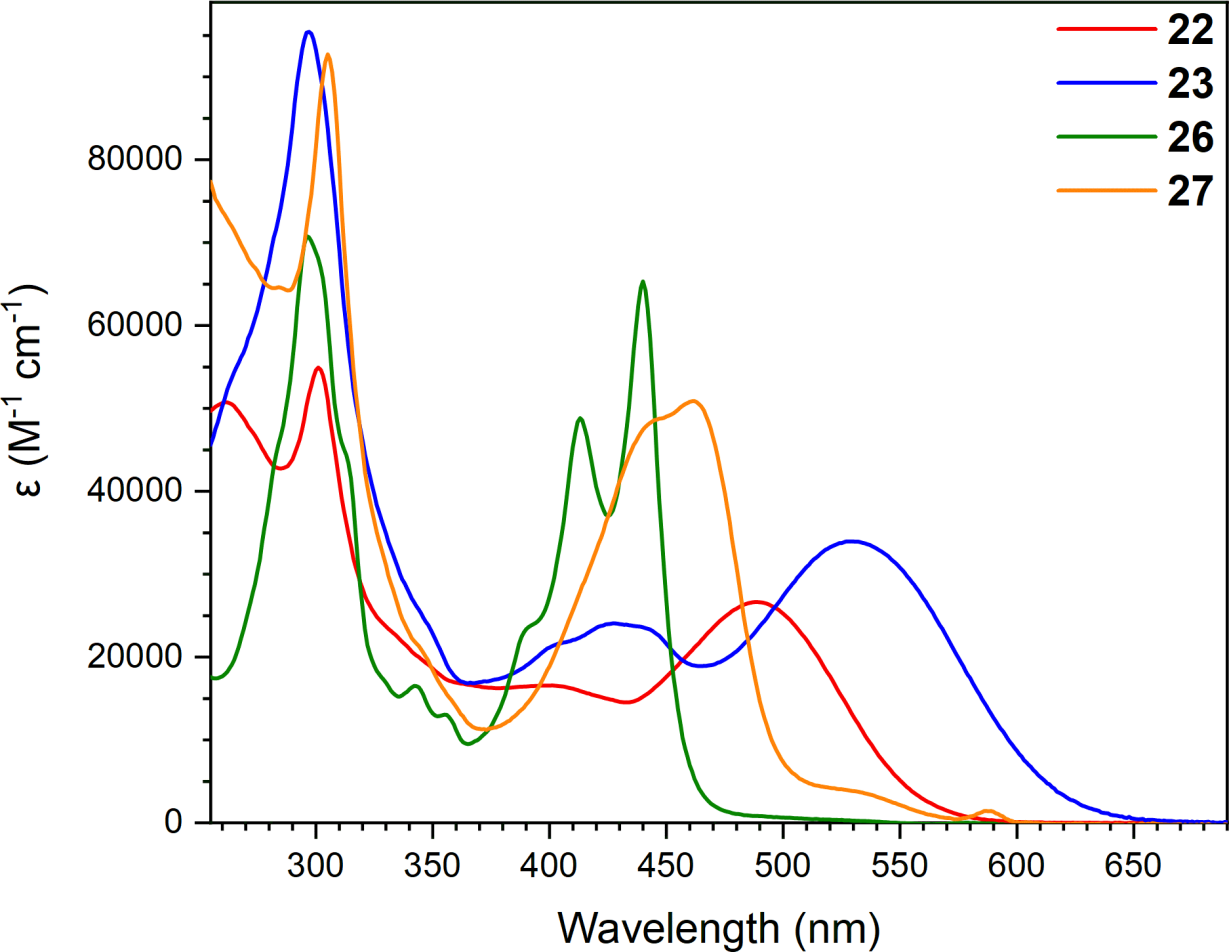
UV–vis absorption spectra of compounds **22**, **23**, **26**, and **27** in CH_2_Cl_2_ at 25 °C.

UV–vis absorption spectra of compounds **22**, **23**, **26**, and **27** are depicted in Figure 5. By comparing donor–acceptor chromophores **22** and **23**, it is observed that the RA moiety of DTF-IF-RA scaffold **23** induces a significant redshift, presumably due to the stronger electron-accepting character of the RA unit (and hence a lower-energy LUMO) compared to the acyclic acetylenic scaffold of compound **22** (in line with first reduction potentials, vide infra). For compound **27**, a shorter longest-wavelength absorption maximum at 461 nm is observed; this is a symmetric compound for which no donor–acceptor “push–pull” system is present (albeit a broad tail to the absorption is observed), in contrast to **22** and **23**. The absorption maxima of compound **26** are significantly blueshifted, presumably due to the smaller conjugated system. The same trend with a shorter longest-wavelength absorption maximum that was observed for compound **27** was also observed for this compound.

The degradation of compound **20** in the presence of light and oxygen is visible as a color change upon leaving a sample of the compound in solution in an open vial, unshielded from light (Figure S2 in Supporting Information File 1). This degradation was investigated by UV–vis absorption spectroscopy; the absorption spectrum was measured over time for three different samples, and a notable change in the longest-wavelength absorption maximum was only observed for the sample that was exposed to both light and oxygen (see Figures S3 and S4 in Supporting Information File 1). We speculate that this degradation is due to the reaction with singlet oxygen generated by the compound as a photosensitizer; indeed, we have recently shown [31] that IF-TTF compounds are reactive towards singlet oxygen at the central fulvene bond but, in contrast, IF-TTFs (without an acetylenic moiety as in **20**) are themselves poor photosensitizers for singlet oxygen.

### Electrochemistry

**Figure 6:**
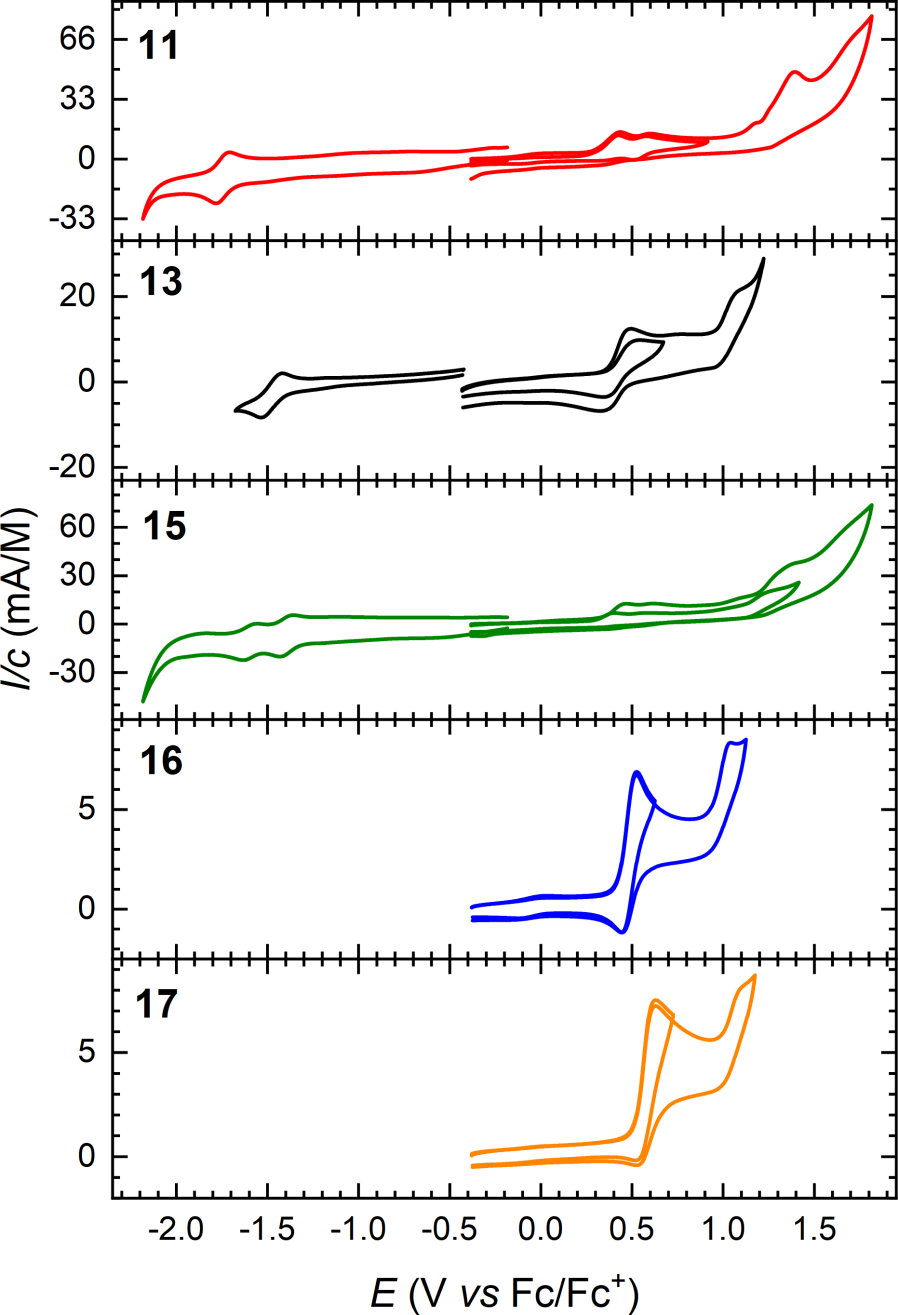
Cyclic voltammograms of compounds **11** (in MeCN), **13** (in CH_2_Cl_2_), **15** (in MeCN), **16** (in CH_2_Cl_2_), and **17** (in CH_2_Cl_2_); supporting electrolyte: 0.1 M Bu_4_NPF_6_, scan rate: 0.1 V/s. All potentials are depicted against the Fc/Fc^+^ redox couple.

**Figure 7:**
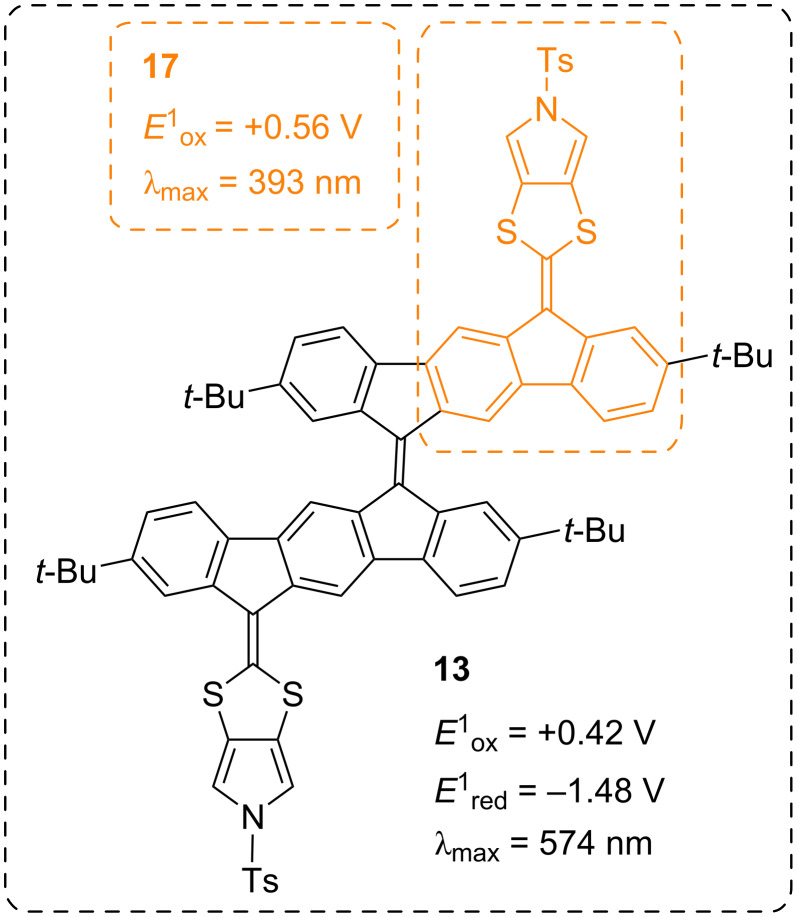
Comparison of properties of compounds **13** and **17**.

Cyclic voltammograms of compounds **11**, **13**, **15**, **16**, and **17** (in MeCN for compounds **11** and **15** and in CH_2_Cl_2_ for compounds **13**, **16**, and **17**, all with 0.1 M Bu_4_NPF_6_ as supporting electrolyte) are shown in Figure 6, and potentials against ferrocene (Fc/Fc^+^) (obtained from differential pulse voltammetry, see Supporting Information File 1) are summarized in Table 2. Compounds **11** and **15** showed two irreversible first oxidations at +0.34 V and +0.38 V vs Fc/Fc^+^, showing that replacing the ketone with the stronger electron withdrawing vinylic diester renders the first oxidation more difficult (by 40 mV). An anodic shift of 40 mV was also observed for the second oxidation. Oppositely, compound **15** underwent a significantly easier first reduction than **11** (−1.00 V vs −1.35 V), and it also underwent a second reduction. The pyrrolo-annelated dimer **13** showed a reversible oxidation at +0.42 V followed by an irreversible oxidation at +1.01 V, and two reversible reductions at −1.48 V and −1.81 V. Here, the acceptor properties are not promoted by incorporating an acceptor unit as in **15**, but instead by the bifluorenylidene motif [32] obtained by dimerizing two pyrrolo-annelated IF-DTF units. Notably, the dimer **13** underwent a first oxidation more readily (by as much as 0.14 V) than the corresponding fluorene-DTF donor **17** (both containing the same *N*-tosylated pyrrolo-DTF unit). The low electrochemical HOMO–LUMO gap of **13** is paralleled by a low-energy longest-wavelength absorption maximum (vide supra, Figure 7).

**Table 2:** Electrochemical data from differential pulse voltammetry of compounds in CH_2_Cl_2_ (with 0.1 M Bu_4_NPF_6_) if not otherwise stated; potentials in volts vs Fc/Fc^+^.

Compound	*E* ^1^_ox_	*E* ^2^_ox_	*E* ^1^_red_	*E* ^2^_red_

**11** ^a^	+0.34	+0.52	−1.35	–
**13** ^b^	+0.42	+1.01	−1.48	−1.81
**15** ^a^	+0.38	+0.56	−1.00	−1.21
**16**	+0.47	+0.99	–	–
**17**	+0.56	+1.07	–	–
**22**	+0.41	+0.76	−1.80	–
**23**	+0.41	+0.81	−1.50	−1.78
**26**	+0.84	–	−1.64	−1.98
**27**	+0.85	–	−1.63	−1.89

^a^In MeCN. ^b^*E/Z* ratio of 4:1.

A quasi-reversible first oxidation was observed at +0.47 V for the fluorene compound **16** and an irreversible oxidation at +0.99 V. Compound **17** experienced a quasi-reversible first oxidation at +0.56 V and an irreversible oxidation at +1.07 V. Thus, the dihydropyrrolo-annelated DTF compound is more easily oxidized than the pyrrolo-annelated DTF compound. These fluorene compounds did not experience a reduction within the potential window.

**Figure 8:**
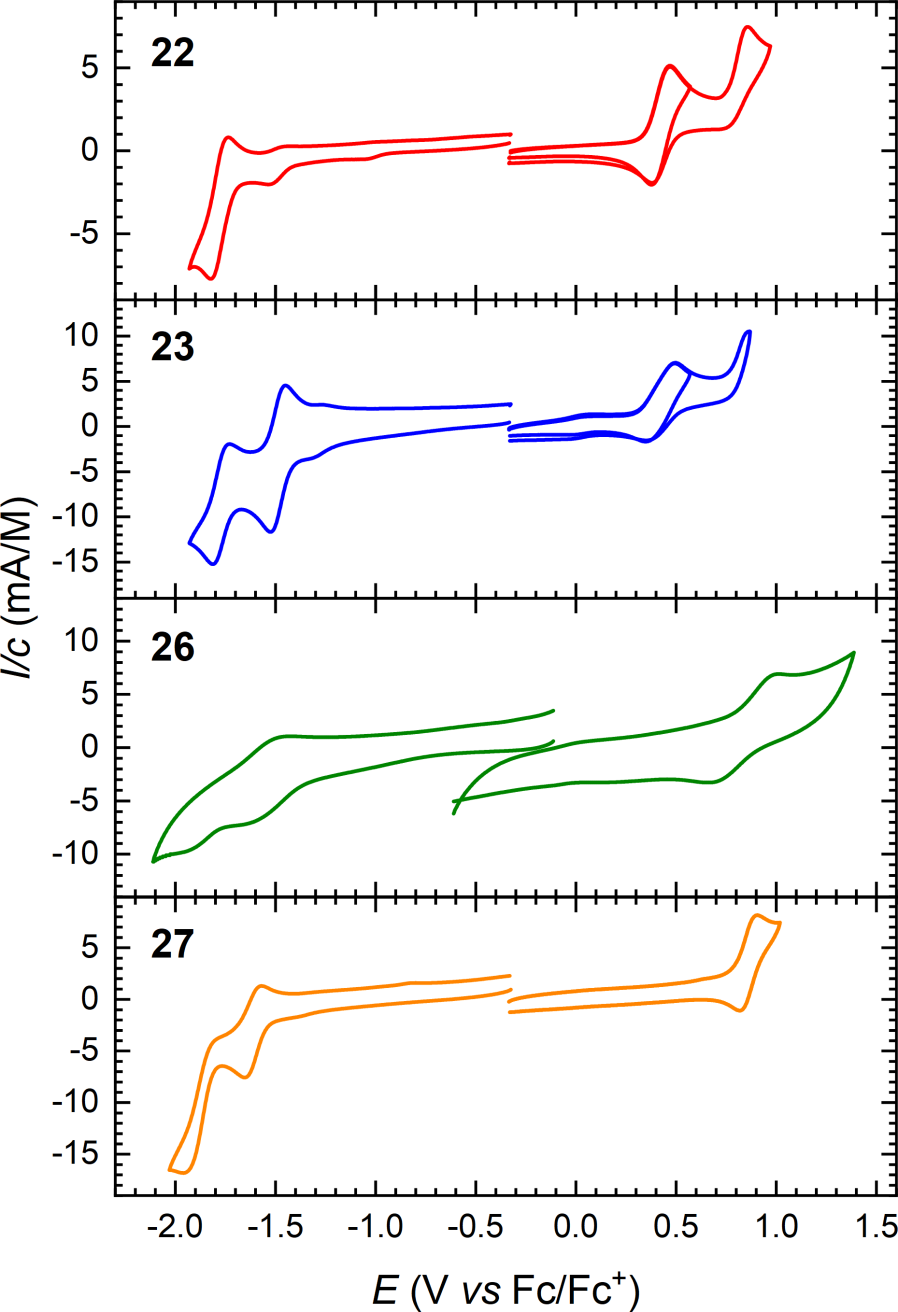
Cyclic voltammograms of compounds **22**, **23**, **26**, and **27** in CH_2_Cl_2_; supporting electrolyte: 0.1 M Bu_4_NPF_6_, scan rate: 0.1 V/s. All potentials are depicted against the Fc/Fc^+^ redox couple.

Cyclic voltammograms of the acetylenic scaffolds **22**, **23**, **26**, and **27** (in CH_2_Cl_2_ with 0.1 M Bu_4_NPF_6_ as supporting electrolyte) are shown in Figure 8. Quasi-reversible one-electron oxidations of the two DTF-functionalized compounds **22** and **23** are observed at +0.41 V followed by irreversible oxidations at +0.76 V (**22**) and +0.81 V (**23**), respectively. One reversible oxidation at +0.84 V and one reversible reduction at −1.64 V were observed for compound **26**, along with one irreversible reduction at −1.98 V. These oxidation and reduction potentials are not significantly different from the potentials observed for compound **27**, namely one quasi-reversible oxidation at +0.85 V and two one-electron reductions at −1.63 V and −1.89 V, indicating that the larger conjugated system of compound **27** does not significantly change the redox properties of the compound. Compounds **26** and **27** lack the DTF donor part and are hence oxidized at significantly higher potentials than the other compounds. On the other hand, they are stronger acceptors than the acetylenic scaffold **22** containing the DTF donor. We have previously [33] studied a related compound in which all four triisopropylsilylethynyl substituents of **26** are replaced by cyano groups; this compound showed superior acceptor properties, being reduced at −0.81 V and −1.09 V vs Fc/Fc^+^ (similar conditions), but no donor properties (thereby contrasting **26** and **27**).

**Figure 9:**
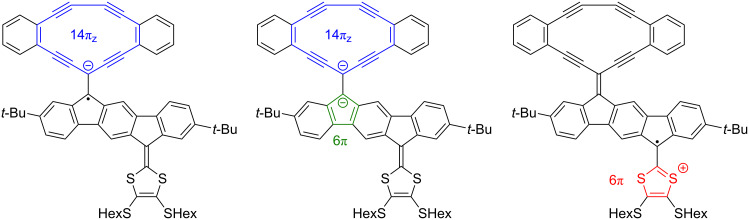
Radical anion (left), dianion (middle), and radical cation (right) of compound **23**; the radical anion has a 14π_z_-aromatic ring (highlighted in blue; only counting 2π-electrons of each triple bond, here defined as those in π_z_ orbitals), the dianion has an additional 6π-aromatic cyclopentadienyl anion (highlighted in green), while the cation has a 6π-aromatic 1,3-dithiolium ring (highlighted in red).

Of the acetylenic scaffolds studied, DTF-IF-RA **23** containing an RA moiety is the strongest acceptor, which we ascribe to gain of 14π_z_-aromaticity of the cyclic moiety of the reduced species (in line with previously studied RA scaffolds [17–18,34]). Indeed, it is reduced more easily by as much as 0.3 V than its corresponding acyclic counterpart, compound **22**, although it contains a π-system of the same size, and it is even reduced more easily by 0.13 V than the acetylenic scaffold **27** containing acetylenic acceptor motifs at both ends of the IF core and hence no DTF donor unit. Compound **23** also undergoes a reversible, second reduction to form the dianion. This compound should gain aromaticity upon either reduction or oxidation as illustrated in Figure 9.

### X-ray crystallographic analysis

**Figure 10:**
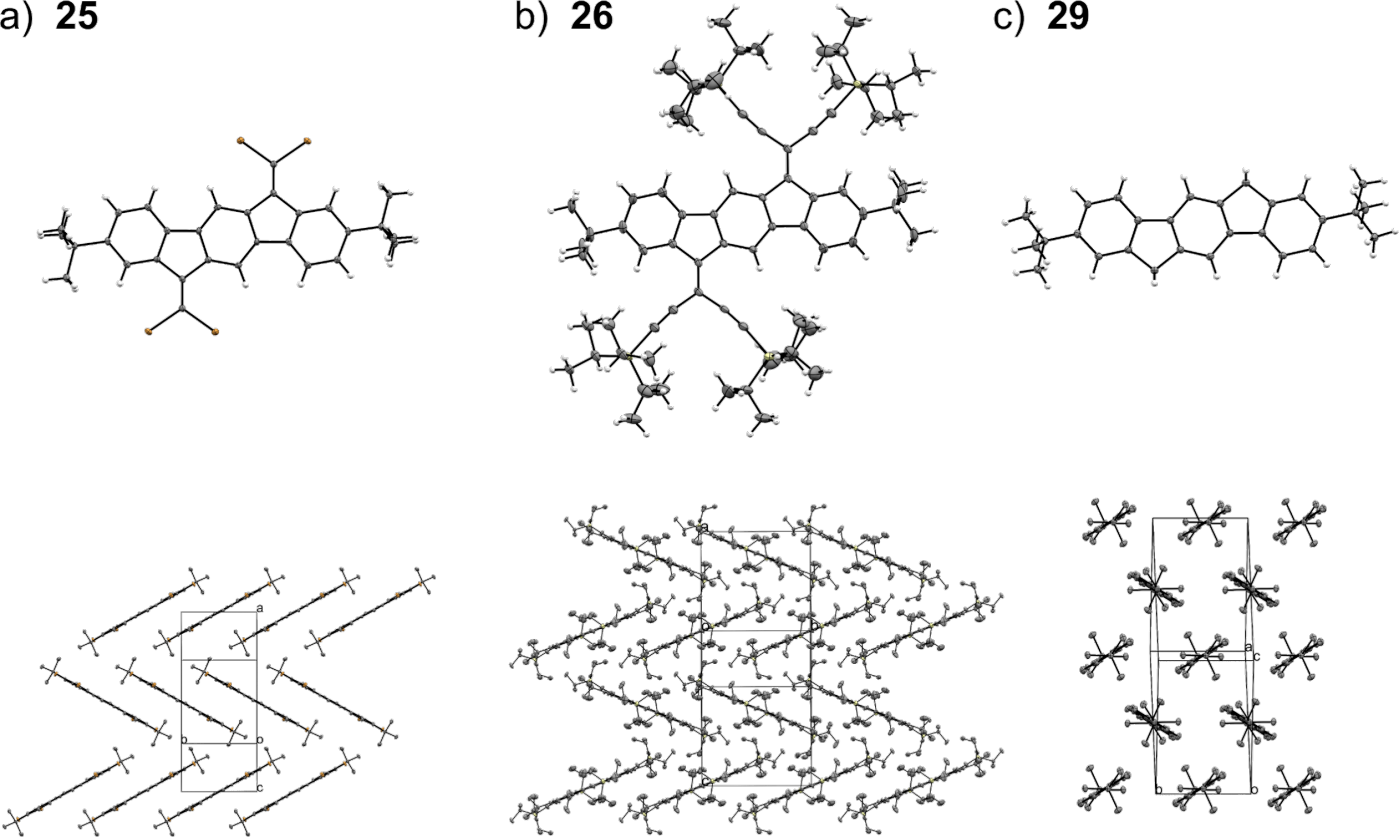
ORTEP plots (50% probability) and crystal packing of compounds a) **25**, b) **26**, and c) **29**. The respective crystal packing of each compound is shown below, in which the hydrogen atoms are omitted for clarity. Atoms are colored grey (carbon), white (hydrogen), brown (bromine), pale-yellow (silicon).

Crystals suitable for single-crystal X-ray diffraction studies were obtained for compounds **25**, **26**, and **29**. Their structures are shown in Figure 10, top, and their respective crystal packings below. All three compounds pack in a herringbone manner in the crystal structure, with the major difference that compound **29** is perpendicular with respect to the herringbone pattern and the related structures (see Figure 10, bottom). Compound **25** packs with an intramolecular distance of 3.41 Å between the planes of the π-systems. Neither compound **26** nor **29** shows π–π interactions in the crystal packing. The large bulkiness of the TIPS groups along with the *tert*-butyl groups in compound **26** prevent these interactions, while for compound **29**, the lack of π–π interactions can be ascribed to the methylene bridges as the hydrogens along with the *tert*-butyl groups prevent good overlap of the π-systems.

**Figure 11:**
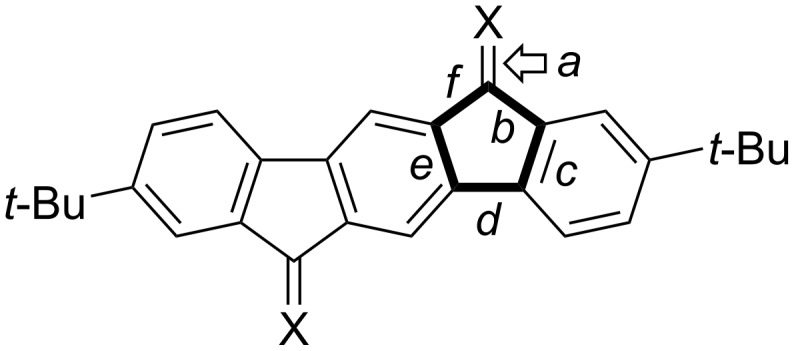
Labels of bonds within five-membered ring.

Table 3 lists the lengths of the bonds (*b*–*f*) within the five-membered rings of the cores as well as the exocyclic C=C double bond (*a*) that is present in compounds **25** and **26** (for bond labels, see Figure 11). A small difference in the exocyclic C=C bond length is observed between **25** and **26**, with the bond in **26** being slightly longer. Bonds *b* and *f* are affected by the moiety X, with the less π-delocalized structure **29** having the longest bonds of 1.51 Å, while only minor differences are observed for bonds *c*, *d*, and *e*.

**Table 3:** Bond lengths (Å) within five-membered rings and of exocyclic C=C double bond (for bond assignments, see Figure 11).

Bond	Compound **25**	Compound **26**	Compound **29**

*a*	1.343(3)	1.362(2)	–
*b*	1.495(3)	1.478(2)	1.5102(15)
*c*	1.408(3)	1.405(2)	1.4030(15)
*d*	1.463(3)	1.468(2)	1.4690(15)
*e*	1.412(3)	1.410(2)	1.4103(15)
*f*	1.495(2)	1.474(2)	1.5090(15)

## Conclusion

In summary, various redox-active chromophores based on the indenofluorene scaffold were synthesized, incorporating different dithiafulvenes and acetylenic scaffolds, such as acetylenic radiaannulenes. The compounds have strong absorptions in the visible region and undergo reversible (or quasi-reversible) oxidations and reductions. We have also presented two new fluorene-extended dithiafulvenes, which also absorb strongly in the visible region and undergo one reversible oxidation, while no reductions were observed for these compounds. Systematic studies show that by small structural modifications, the optical and electrochemical HOMO–LUMO gaps can be finely tuned – with first oxidations and reductions that can be adjusted by several hundreds of millivolts for donor–acceptor IF scaffolds. Introduction of both the dithiafulvene and radiaannulene units along the indenofluorene scaffold provided a donor–acceptor compound covering a particularly broad absorption profile and with a redshifted longest-wavelength absorption maximum relative to most of the compounds (529 nm in dichloromethane), which can be related to the fact that it is both a good donor and a good acceptor as shown electrochemically. This compound stands out as gaining aromaticity in one of its appendages along the IF core upon either reduction (generation of 14π_z_-aromatic ring) or oxidation (generation of 1,3-dithiolium ring).

Synthetically, the work relies on using indenofluorene diones as key building blocks for performing olefination reactions, such as phosphite- or Lawesson’s reagent-mediated couplings, Ramirez/Corey–Fuchs dibromo-olefinations, and Knoevenagel condensations. In particular, the acetylenic scaffolds presented in this work may be useful precursors for even more elaborate, conjugated and carbon-rich structures in future work.

## Experimental

Anhydrous MeOH was obtained by distillation from activated Mg and stored over 3 Å molecular sieves, or by drying over 3 Å molecular sieves. All remaining anhydrous solvents were obtained from a solvent drying tower (IT model PS-MD-05). HPLC grade solvents were used unless otherwise specified. Purification by chromatography was performed using silica gel (flash: 40–63 μm, Sepacore^®^ Flash Systems X10/X50: 40–63 μm). TLC was performed using aluminum sheets covered with silica gel coated with fluorescent indicator. NMR spectra were recorded on a Bruker instrument at 500 MHz and 126 MHz for ^1^H and ^13^C NMR, respectively. Deuterated chloroform (CDCl_3_, ^1^H = 7.26 ppm, ^13^C = 77.16 ppm), deuterated CH_2_Cl_2_ (CD_2_Cl_2_, ^1^H = 5.32 ppm, ^13^C = 54.00 ppm), deuterated DMSO ((CD_3_)_2_SO, ^1^H = 2.50 ppm, ^13^C = 39.53 ppm), deuterated acetone ((CD_3_)_2_CO, ^1^H = 2.05 ppm, ^13^C = 29.84 ppm), or deuterated benzene (C_6_D_6_, ^1^H = 7.16 ppm, ^13^C = 128.39 ppm) were used as solvents and internal references. Chemical shift values are referenced to the ppm scale and coupling constants are expressed in Hertz (Hz). HRMS analysis was performed on a Bruker SolariX XR MALDI-FT-ICR instrument with dithranol as matrix. Melting points are not corrected.

### UV–vis absorption spectroscopy

UV–vis absorption spectra were recorded on a Varian Cary 50 UV–vis spectrophotometer scanning between 800 and 200 nm. All spectra were recorded with baseline correction in CH_2_Cl_2_ or toluene (HPLC grades) at 25 °C in a quartz cuvette with a 10 mm path length.

### Electrochemistry

Cyclic voltammograms (CV) and differential pulse voltammograms (DPV) were obtained using an Autolab PGSTAT12 instrument and Nova 1.11 software with a scan rate of 0.1 V/s for the CVs. A silver wire immersed in a 0.1 M Bu_4_NPF_6_ solution in CH_2_Cl_2_ separated from the analyte solution by a frit was used as the reference electrode, a Pt wire was used as the counter electrode, and a platinum disk (diameter = 1.6 mm) or a glassy carbon disk (3 mm) was used as the working electrode. The reference electrode was separated from the solution containing the substrate by a ceramic frit. Measured potentials were referenced to ferrocene/ferrocenium (Fc/Fc^+^) redox couple, measured before and after the experiment. A 0.1 M solution of NBu_4_PF_6_ was used as electrolyte. All solutions were purged with Ar prior to measurements.

### Crystallography

All single crystal X-ray diffraction data for compounds **25**, **26**, and **29** were collected on a Bruker D8 VENTURE diffractometer equipped with a Mo Kα X-ray (λ = 0.71073 Å). The data collections were done at 100 K. All data were integrated with SAINT and a multi-scan absorption correction using SADABS was applied [35–36]. The structure was solved by direct methods using SHELXT and refined by full-matrix least-squares methods against *F*2 by SHELXL-2019/2 [37–38]. The data for the compounds have been deposited with the Cambridge Crystallographic Data Centre [39]. The CIF files (Supporting Information Files 2–4) and reports were generated using FinalCIF [40].

### Synthesis

Compounds **1** [19], **2** [21], **3** [21], **4** [14], and **6** [20] were synthesized according to literature procedures, and compounds **7** and **8** were synthesized according to modified literature procedures [21]. Representative synthetic protocols are provided below, while protocols for **7**, **8**, **10**–**12**, **14**, **15**, **17**, **20**–**22**, **24**–**27** are included in Supporting Information File 1.

#### Compound **9**

A solution of **1** (139 mg, 352 μmol) and **2** (176 mg, 534 μmol) in anhydrous toluene (5 mL) and P(OEt)_3_ (10 mL) was heated to reflux for 5 h, resulting in a color change from orange to dark red. The reaction mixture was then allowed to cool to rt before it was concentrated under reduced pressure. The resulting dark red solid was purified by flash column chromatography (SiO_2_, 20% EtOAc/heptane), and recrystallization from CH_2_Cl_2_/MeOH followed by centrifugation yielded **9** (136 mg, 57%) as an orange solid. *R*_f_ = 0.18 (70% CH_2_Cl_2_/heptane); mp 178–181 °C; ^1^H NMR (500 MHz, CDCl_3_) δ 7.99 (s, 1H), 7.80 (d, *J* = 8.4 Hz, 2H), 7.76 (s, 1H), 7.72–7.71 (m, 2H), 7.68 (d, *J* = 8.0 Hz, 1H), 7.58–7.44 (m, 2H), 7.44–7.35 (m, 3H), 4.50 (s, 4H), 2.44 (s, 3H), 1.43 (s, 9H), 1.36 (s, 9H) ppm; ^13^C NMR (126 MHz, CDCl_3_) δ 194.1, 152.7, 151.1, 148.7, 148.0, 144.7, 143.5, 142.3, 142.3, 138.8, 137.1, 135.5, 135.1, 133.6, 132.3, 131.4, 130.4, 129.1, 128.8, 127.7, 121.0, 123.4, 121.6, 120.0, 119.7, 119.6, 115.7, 114.5, 52.7, 52.6, 35.4, 35.2, 31.9, 31.4, 21.8 ppm; five sp^2^ signals missing, presumably due to overlap. HRMS (MALDI^+^, FT-ICR, dithranol, *m*/*z*) [M + H^+^] calcd for C_40_H_38_NO_3_S_3_^+^, 676.2008; found, 676.2019.

#### Compound **13**

A solution of **4** (62.0 mg, 92.0 μmol) and Lawesson’s reagent (23.1 mg, 57.0 μmol) in anhydrous, N_2_-degassed toluene (20 mL) was heated to reflux for 21 h. The reaction mixture was then allowed to cool to rt, diluted with toluene (50 mL), washed with 1 M NaOH (3 × 50 mL), and then with H_2_O (3 × 50 mL). The organic phase was dried over MgSO_4_ and concentrated under reduced pressure. The residue was purified by flash column chromatography (SiO_2_, 20% EtOAc/heptane), yielding **13** (15.5 mg, 26%) as a purple solid. *R*_f_ = 0.23 (20% EtOAc/heptane); ^1^H NMR (500 MHz, CDCl_3_) δ 8.64 (s, 2H), 8.50 (s, 2H), 8.12 (s, 2H), 7.96 (s, 2H), 7.83 (d, *J* = 8.4 Hz, 4H), 7.64 (d, *J* = 8.0 Hz, 2H), 7.50 (d, *J* = 8.0 Hz, 2H), 7.34 (m, 6H), 7.29 (d, *J* = 8.4 Hz, 2H), 7.23 (s, 2H), 7.20 (s, 2H), 2.42 (s, 6H), 1.44 (s, 18H), 1.27 (s, 18H) ppm (*E*:*Z* ratio 4:1; ^1^H NMR signals reported for the *E* isomer); ^13^C NMR (126 MHz, CDCl_3_) δ 150.2, 149.9, 145.8, 143.6, 140.7, 140.5, 139.1, 139.0, 138.6, 137.9, 137.4, 137.0, 136.4, 135.5, 130.4, 127.2, 126.7, 126.6, 126.1, 125.4, 124.1, 123.7, 120.8, 119.2, 119.1, 117.9, 115.0, 111.5, 111.4, 55.7, 35.3, 35.2, 35.2, 35.1, 32.0, 31.9, 31.8, 31.7, 31.6, 29.9, 29.5, 29.1, 22.8, 21.8, 14.3 ppm (*E*:*Z* ratio 4:1; sp^2^-C signals missing, presumably due to overlap): HRMS (MALDI^+^, FT-ICR, dithranol, *m*/*z*) [M^•+^] calcd for C_80_H_70_N_2_O_4_S_6_^•+^, 1314.3654; found, 1314.3631.

#### Compound **16**

To a flame-dried vial equipped with a magnetic stir bar were added **2** (69 mg, 209 μmol), **5** (28 mg, 153 μmol), and Lawesson’s reagent (63 mg, 155 μmol). Dry toluene (5 mL) degassed with N_2_ for 15 min was added, and the solution was heated to 105 °C for 18.5 h. The reaction mixture was then allowed to cool to rt, diluted with toluene (20 mL), and washed with 1 M NaOH (3 × 20 mL), and then with H_2_O (20 mL). The yellow precipitate in the aqueous phase was isolated by filtration and washed with H_2_O before it was purified by flash column chromatography (SiO_2_, 50%–100% CH_2_Cl_2_/heptane) yielding **16** (18 mg, 39 μmol, 25%) as a yellow solid. *R*_f_ = 0.18 (50% CH_2_Cl_2_/heptane); mp > 260 °C; ^1^H NMR (500 MHz, CD_2_Cl_2_) δ 7.84 (d, *J* = 7.5 Hz, 2H), 7.77 (d, *J* = 8.1 Hz, 2H), 7.75 (d, *J* = 7.5 Hz, 2H), 7.41 (td, *J* = 7.5, 1.1 Hz, 2H), 7.39 (d, *J* = 8.1 Hz, 2H), 7.34 (td, *J* = 7.5, 1.1 Hz, 2H), 4.46 (s, 4H), 2.42 (s, 3H) ppm; ^13^C NMR (126 MHz, CD_2_Cl_2_) δ 145.0, 138.5, 137.3, 134.0, 130.6, 128.8, 128.0, 127.5, 126.4, 123.4, 120.2, 21.7 ppm; two sp^2^-C carbon signals missing, presumably due to overlap; HRMS (MALDI^+^, FT-ICR, dithranol, *m*/*z*) [M^•+^] calcd for C_25_H_19_NO_2_S_3_^•+^, 461.0572; found, 461.0577.

#### Compound **18**

To an Ar-degassed solution of PPh_3_ (845 mg, 3.22 mmol) and CBr_4_ (560 mg, 1.69 mmol) in anhydrous toluene (20 mL) was added **6** (250 mg, 0.351 mmol). The reaction mixture was heated to reflux and stirred under a N_2_ atmosphere for 30 h before it was cooled to rt and filtered through a plug of SiO_2_ (CH_2_Cl_2_ as eluent) and concentrated in vacuum. Flash column chromatography (10% CH_2_Cl_2_/heptane) yielded **18** (246 mg, 81%) as an orange solid. *R*_f_ = 0.29 (10% CH_2_Cl_2_/heptane); ^1^H NMR (500 MHz, CDCl_3_) δ 8.99 (d, *J* = 0.7 Hz, 1H), 8.71 (d, *J* = 1.5 Hz, 1H), 7.94 (d, *J* = 0.7 Hz, 1H), 7.76–7.75 (m, 2H), 7.71 (d, *J* = 8.0 Hz, 1H), 7.48 (dd, *J* = 8.0, 1.5 Hz, 1H), 7.38 (dd, *J* = 8.0, 1.5 Hz, 1H), 3.01–2.96 (m, 4H), 1.85–1.68 (m, 4H), 1.51–1.47 (m, 4H), 1.45 (s, 9H), 1.40 (s, 9H), 1.37–1.30 (m, 8H), 0.92–0.88 (m, 6H) ppm; ^13^C NMR (126 MHz, CDCl_3_) δ 150.5, 150.4, 139.8, 139.6, 138.8, 138.5, 138.3, 138.1, 137.6, 137.3, 136.1, 135.8, 129.5, 128.4, 126.6, 123.3, 123.2, 121.4, 120.1, 119.2, 118.9, 117.3, 113.8, 89.1, 36.9, 36.8, 35.3, 35.3, 31.9, 31.7, 31.6, 31.5, 30.1, 30.0, 28.5, 22.7, 14.2, 14.2 ppm; two sp^3^-C signals missing, presumably due to overlap; HRMS (MALDI^+^, FT-ICR, dithranol, *m*/*z*) [M^•+^] calcd for C_44_H_52_Br_2_S_4_^•+^, 868.1293; found, 868.1287.

#### Compound **19**

To a N_2_-degassed solution of **18** (90 mg, 0.10 mmol) in anhydrous THF (5 mL) and Et_3_N (5 mL) were added N_2_-degassed trimethylsilylacetylene (0.20 mL, 1.4 mmol), Pd(PPh_3_)_2_Cl_2_ (15 mg, 0.021 mmol), and CuI (5.0 mg, 0.026 mmol). The reaction mixture was stirred at rt under a N_2_ atmosphere for 4 h before it was filtered through a plug of SiO_2_ (CH_2_Cl_2_ as eluent) and concentrated under reduced pressure. Purification by flash column chromatography (SiO_2_, 10–15% CH_2_Cl_2_/heptane) yielded **19** (62 mg, 66%) as a purple solid (red in solution). *R*_f_ = 0.31 (15% CH_2_Cl_2_/heptane); ^1^H NMR (500 MHz, CDCl_3_) δ 9.07 (s, 1H), 8.81 (d, *J* = 1.7 Hz, 1H), 7.91 (s, 1H), 7.76 (d, *J* = 1.7 Hz, 1H), 7.72 (d, *J* = 8.0 Hz, 1H), 7.65 (d, *J* = 8.0 Hz, 1H), 7.41 (dd, *J* = 8.0, 1.7 Hz, 1H), 7.37 (dd, *J* = 8.0, 1.7 Hz, 1H), 3.01–2.96 (m, 4H), 1.79–1.72 (m, 4H), 1.54–1.48 (m, 4H), 1.45 (s, 9H), 1.40 (s, 9H), 1.35–1.32 (m, 8H), 0.92–0.89 (m, 6H), 0.44 (s, 9H), 0.35 (s, 9H) ppm; ^13^C NMR (126 MHz, CDCl_3_) δ 150.6, 150.2, 146.7, 139.6, 138.8, 138.3, 138.0, 137.6, 137.5, 136.0, 135.5, 129.5, 128.5, 126.9, 123.2, 123.2, 121.8, 120.1, 119.0, 118.8, 117.3, 113.9, 104.9, 104.5, 104.5, 104.3, 99.5, 36.9, 36.8, 35.3, 35.3, 31.9, 31.8, 31.6, 31.5, 30.1, 30.0, 28.5, 22.7, 22.7, 14.2, 14.2, 0.3, 0.1 ppm; one sp^2^-C signal and one sp^3^-C signal missing, presumably due to overlap; HRMS (MALDI^+^, FT-ICR, dithranol, *m*/*z*) [M^•+^] calcd for C_54_H_70_S_4_Si_2_^•+^, 903.3972; found, 903.3985.

#### Compound **23**

In a manner similar to [41], TBAF (1 M in THF, 0.2 mL, 0.2 mmol) was added to a solution of **22** (93 mg, 0.073 mmol) in THF (10 mL), and the reaction mixture was stirred at rt for 45 min before it was filtered through a plug of SiO_2_ (CH_2_Cl_2_ as eluent) and concentrated under reduced pressure to a volume of approx. 2 mL. The resulting solution was diluted with CH_2_Cl_2_ (50 mL). A solution of CuCl (7.0 mg, 0.070 mmol) in CH_2_Cl_2_ (5 mL) and TMEDA (0.10 mL, 0.67 mmol) was added along with 4 Å molecular sieves, and the reaction mixture was stirred in an open flask at rt for 3 days before it was filtered through a plug of SiO_2_ (CH_2_Cl_2_ as eluent) and concentrated under reduced pressure. Flash column chromatography (30% CH_2_Cl_2_ (technical grade stabilized with 0.2% EtOH)/heptane) yielded **23** (33 mg, 47%) as a dark green solid. *R*_f_ = 0.20 (40% CH_2_Cl_2_/heptane); ^1^H NMR (500 MHz, CDCl_3_) δ 9.17 (s, 1H), 8.83 (d, *J* = 1.7 Hz, 1H), 7.89 (d, *J* = 7.8 Hz, 1H), 7.88 (s, 1H), 7.80 (d, *J* = 7.8 Hz, 1H), 7.76–7.71 (m, 2H), 7.60 (d, *J* = 8.0 Hz, 1H), 7.51–7.28 (m, 8H), 3.02–2.97 (m, 4H), 1.80–1.73 (m, 4H), 1.52–1.50 (m, 4H), 1.48 (s, 9H), 1.47 (s, 9H), 1.37–1.32 (m, 8H), 0.96–0.85 (m, 6H) ppm; ^13^C NMR (126 MHz, CDCl_3_) δ 150.5, 150.1, 148.3, 140.0, 139.0, 138.7, 138.7, 138.5, 137.8, 137.4, 136.0, 135.8, 131.4, 131.4, 130.6, 129.7, 129.5, 129.5, 129.0, 129.0, 128.8, 128.6, 128.6, 128.5, 127.0, 125.2, 125.0, 123.2, 123.1, 121.8, 120.2, 119.0, 117.9, 114.1, 99.6, 96.9, 95.9, 95.4, 94.5, 88.0, 87.8, 82.4, 81.2, 36.9, 36.8, 35.3, 35.2, 32.0, 31.9, 31.6, 31.5, 30.1, 30.0, 28.5, 22.7, 22.7, 14.2, 14 ppm; one signal missing in the aromatic region and one signal missing in the aliphatic region, presumably due to overlap; HRMS (MALDI^+^, FT-ICR, dithranol, *m*/*z*) [M^•+^] calcd for C_82_H_102_S_4_Si_2_^•+^, 956.3572; found, 956.3620.

#### Compound **29**

To a 250 mL round-bottomed flask equipped with a reflux condenser and containing a magnetic stir bar, diethylene glycol (125 mL) and KOH (2.67 g, 47.7 mmol) were added. The solution was degassed with Ar for 30 min after which **5** (461 mg, 1.17 mmol) was added. Then, N_2_H_4_·H_2_O (2.4 mL, 50.0 mmol) was added slowly, resulting in a color change to black within 30 min. The reaction was carried out under inert N_2_ atmosphere. The reaction mixture was then heated to 185–190 °C for 48 h after which it was cooled to 100 °C, poured onto ice (400 mL), and acidified with aq HCl (20 mL, 6 M), resulting in an orange precipitate. The ice was allowed to melt, and the precipitate was filtered, washed with H2O (100 mL), and dissolved in EtOAc (200 mL), after which the volatiles were removed under reduced pressure yielding compound **29** as a light orange crystalline solid (375 mg, 1.02 mmol, 88%). mp > 250 °C; ^1^H NMR (500 MHz, CDCl3) δ 7.89 (s, 2H), 7.71 (d, *J* = 8.0, 2H), 7.59 (s, 2H), 7.42 (d, *J* = 8.0, 2H), 3.95 (s, 4H), 1.39 (s, 18H) ppm; ^13^C NMR (126 MHz, CDCl_3_) δ 149.8, 143.8, 142.5, 140.6, 139.5, 124.1, 122.1, 119.2, 116.3, 37.0, 35.0, 31.8 ppm; HRMS (MALDI^+^, FT-ICR, dithranol, *m*/*z*) [M^•+^] calcd for C_28_H_30_^•+^, 366.2342; found, 366.2344.

## Supporting Information

File 1 Synthetic protocols, UV–vis and NMR spectra, differential pulse voltammograms, and X-ray crystallographic data.

File 2 Crystallographic information file of compound **25**.

File 3 Crystallographic information file of compound **26**.

File 4 Crystallographic information file of compound **29**.

## Data Availability

The data that supports the findings of this study is available from the corresponding author upon reasonable request.
