# Supplementary material for: Multi-redox indenofluorene chromophores incorporating dithiafulvene donor and ene/enediyne acceptor units
Source: Beilstein J Org Chem. 2024 Jan 15;20:59–73. doi: 10.3762/bjoc.20.8 (PMC10804541; doi:10.3762/bjoc.20.8)
Supplement: File 1 — Synthetic protocols, UV–vis and NMR spectra, differential pulse voltammograms, and X-ray crystallographic data. [file Beilstein_J_Org_Chem-20-59-s001.pdf]

BEILSTEIN JOURNAL OF ORGANIC CHEMISTRY

Supporting Information

for

Multi-redox indenofluorene chromophores incorporating dithiafulvene donor and ene/enediyne acceptor units

Christina Schøttler, Kasper Lund-Rasmussen, Line Broløs, Philip Vinterberg, Ema Bazikova, Viktor B. R. Pedersen and Mogens Brøndsted Nielsen

Beilstein J. Org. Chem. **2024,** *20,* 59–73. doi:10.3762/bjoc.20.8

Synthetic protocols, UV–vis and NMR spectra, differential pulse voltammograms, and X-ray crystallographic data

License and Terms: This is a supporting information file under the terms of the Creative Commons Attribution License (https://creativecommons.org/licenses/by/4.0). Please note that the reuse, redistribution and reproduction in particular requires that the author(s) and source are credited and that individual graphics may be subject to special legal provisions.
The license is subject to the *Beilstein Journal of Organic Chemistry* terms and conditions: (https://www.beilstein-journals.org/bjoc/terms)

Table of Contents

Synthetic protocols …… p. S2

UV–vis absorption studies of compounds 10 and 11 (different solvents) …… p. S16

UV–vis absorption studies of 20 (degradation studies) …… p. S16

^{1}H and ^{13}C NMR spectra …… p. S18

Electrochemistry …… p. S39

X-ray crystallographic data …… p. S42

References …… p. S84

Synthetic protocols

General methods. Anhydrous MeOH was obtained by distillation from activated Mg and stored over 3 Å molecular sieves, or by drying over 3 Å molecular sieves. All remaining anhydrous solvents were obtained from a solvent drying tower (IT model PS-MD-05). HPLC grade solvents were used unless otherwise specified. Purification by chromatography was performed using silica gel (flash: 40–63 μm, Sepacore® Flash Systems X10/X50: 40–63 μm). TLC was performed using aluminum sheets covered with silica gel coated with fluorescent indicator. NMR spectra were recorded on Bruker instrument at 500 MHz and 126 MHz for ^{1}H and ^{13}C NMR, respectively. Deuterated chloroform ($CDCl_3$, ^{1}H = 7.26 ppm, ^{13}C = 77.16 ppm), deuterated CH_2Cl_2 (CD_2Cl_2, ^{1}H = 5.32 ppm, ^{13}C = 54.00 ppm), deuterated DMSO ($(CD_3)_2SO$, ^{1}H = 2.50 ppm, ^{13}C = 39.53 ppm), deuterated acetone ($(CD_3)_2CO$, ^{1}H = 2.05 ppm, ^{13}C = 29.84 ppm), or deuterated benzene (C_6D_6, ^{1}H = 7.16 ppm, ^{13}C = 128.39 ppm) were used as solvents and internal references. Chemical shift values are referenced to the ppm scale and coupling constants are expressed in Hertz (Hz). HRMS analysis was performed on a Bruker SolariX XR MALDI-FT-ICR instrument with dithranol as matrix. Melting points are not corrected.

Synthetic protocols for **9**, **13**, **16**, **18**, **19**, **23**, and **29** are included in the main article.

Compound 7

4,5-Bis(bromomethyl)-1,3-dithiole-2-thione (1.31 g, 4.09 mmol) was dissolved in a mixture of anhydrous MeCN (100 mL) and anhydrous THF (50 mL). Hexylamine (0.850 mL, 6.22 mmol) and cesium carbonate (5.42 g, 16.6 mmol) were added to the stirring mixture, which was then heated to reflux for 1 h. The reaction mixture was then cooled to rt before it was filtered, and the filtrate was concentrated under reduced pressure. The residue was dissolved in CH_2Cl_2 (150 mL), washed with water (4 × 50 mL), dried over $MgSO_4$, and concentrated under reduced pressure resulting in a brown oil that was purified by flash column chromatography (SiO_2, 20% EtOAc/heptane), yielding compound **7** (811 mg, 77%) as a yellow oil, which solidified upon cooling. R_f = 0.32 (20% EtOAc/heptane). M.p.: 40-42 °C. ^{1}H NMR (500 MHz, $CDCl_3$) δ 3.84 (s, 4H), 2.74 (t, J = 7.4 Hz, 2H), 1.50 (p, 7.7 Hz, 2H), 1.42 – 1.17 (m, 6H), 0.89 (t, J = 6.9 Hz, 3H). ^{13}C NMR (126 MHz, $CDCl_3$) δ 217.7, 138.7, 57.6, 56.5, 31.8, 28.9, 27.0, 22.7, 14.2 ppm. HRMS (MALDI$^+$, FT-ICR, dithranol) m/z = 260.0595 [M + H^+], calcd for ($C_{11}H_{18}NS_3^+$) = 260.0596.

Compound 8

A mixture of compound **7** (311 mg, 1.20 mmol) and DDQ (599 mg, 2.64 mmol) in anhydrous PhMe (15 mL) was heated to reflux for 2 hours. The reaction mixture was then cooled to rt and filtered. The filtrate was washed with 10% aqueous NaOH (3 × 10 mL), dried over $MgSO_4$, and filtered. The organic phase was then filtered through a silica plug (SiO_2, PhMe) and concentrated under reduced pressure. The residue was purified by flash column chromatography (SiO_2, 10% EtOAc/heptane), yielding compound **8** (220 mg, 71%) as a brown oil. R_f = 0.33 (20% EtOAc/heptane). ^{1}H NMR (500 MHz, $(CD_3)_2SO$) δ 7.15 (s, 2H), 4.02 (t, J = 7.1 Hz, 2H), 1.71 (p, J = 7.1 Hz, 2H), 1.46 – 1.04 (m, 6H), 0.84 (t, J = 7.9 Hz, 3H) ppm. ^{13}C NMR (126 MHz, $(CD_3)_2SO$) δ 219.5, 121.0, 113.3, 50.3, 30.9, 30.7, 25.6, 22.0,

13.8 ppm. HRMS ($MALDI^+$, FT-ICR, dithranol) m/z = 258.0439 [M + H^+], calcd for ($C_{11}H_{16}NS_3^+$) = 258.0439.

Compound 10

A solution of **1** (85 mg, 223 μmol) and **7** (92 mg, 354 μmol) in anhydrous toluene (5 mL) and $P(OEt)_3$ (10 mL) was heated to reflux for 5 h, resulting in a color change from red to dark red. The reaction mixture was then allowed to cool to rt before it was concentrated under reduced pressure. The resulting dark red solid was purified by flash column chromatography using Sepacore® Flash Systems X10/X50 (SiO_2, 1%–10% EtOAc/heptane), and recrystallization from CH_2Cl_2/MeOH followed by centrifugation yielded **10** (54 mg, 40%) as a dark red solid. R_f = 0.32 (20% EtOAc/heptane). M.p.: 180-182 °C. ^{1}H NMR (500 MHz, CD_2Cl_2) δ 8.02 (d, J = 0.8 Hz, 1H), 7.94 (d, J = 0.8 Hz, 1H), 7.87 (d, J = 1.6 Hz, 1H), 7.78 (d, J = 8.0 Hz, 1H), 7.68 (dd, J = 1.7, 0.9 Hz, 1H), 7.59 – 7.51 (m, 2H), 7.42 (dd, J = 8.0, 1.6 Hz, 1H), 3.91 (s, 4H), 2.80 (t, J = 7.4 Hz, 2H), 1.53 (s, 9H), 1.45 (s, 9H), 1.59 – 1.54 (m, 2H), 1.42 – 1.33 (m, 6H), 0.92 (t, J = 7.1, 3H) ppm. ^{13}C NMR (126 MHz, CD_2Cl_2) δ 193.9, 152.8, 151.2, 143.5, 142.7, 142.6, 138.4, 137.7, 135.9, 134.9, 132.6, 132.3, 131.9, 131.7, 123.6, 121.8, 121.5, 120.2, 119.9, 119.7, 115.7, 114.7, 57.5, 57.4, 57.0, 35.5, 35.4, 32.2, 31.9, 31.4, 29.2, 27.3, 23.1, 14.3 ppm; one sp^3-C signal and four sp^2-C signals missing, presumably due to overlap. HRMS ($MALDI^+$, FT-ICR, dithranol) m/z = 606.2866 [M + H^+], calcd for ($C_{39}H_{44}NOS_2^+$) = 606.2859.

Compound 11

Method 1 – from IF dione ***1***

A solution of **1** (89 mg, 226 μmol) and **8** (95 mg, 350 μmol) in anhydrous toluene (5 mL) and $P(OEt)_3$ (10 mL) was heated to reflux for 5 h, resulting in a color change from orange to dark red. The reaction mixture was then allowed to cool to rt before it was concentrated under

reduced pressure. The resulting dark red solid was purified by flash column chromatography (SiO_2, 20% EtOAc/heptane), and recrystallization from CH_2Cl_2/MeOH followed by centrifugation yielded **11** (74 mg, 59%) as a red solid.

Method 2 – from ***10***

To a solution of **10** (50 mg, 83 μmol) in PhCl (10 mL) was added DDQ (49 mg, 216 μmol), before it was heated to reflux for 4 h. The reaction mixture was then allowed to cool to rt before it was filtered through a silica plug (SiO_2, CH_2Cl_2) and concentrated under reduced pressure. The residue was purified by flash column chromatography (SiO_2, 10% EtOAc/heptane), and recrystallization from CH_2Cl_2/MeOH followed by centrifugation yielded **11** (29 mg, 58%) as a red solid.

Method 3 – from ***12***

A solution of **12** (22.0 mg, 42.3 μmol) in anhydrous DMF (4 mL) was degassed with Ar for 15 min before NaH (60% in mineral oil suspension, 19.3 mg, 483 μmol) was added, and the reaction mixture was stirred at rt for 15 min resulting in a color change from dark red to dark blue. Then, 1-bromohexane (0.06 mL, 42 μmol) was added, and the reaction mixture was stirred at rt for 2 h, resulting in a color change to dark red. Brine (40 mL) was added dropwise under stirring, and the reaction mixture was extracted with CH_2Cl_2 (80 mL, then 2 × 50 mL). The combined organic phases were washed with brine (3 × 100 mL), dried over $MgSO_4$, filtered, and concentrated under reduced pressure. The residue was purified by flash column chromatography (SiO_2, 10% EtOAc/heptane), yielding **11** (20.6 mg, 91%) as a red solid. R_f = 0.28 (20% EtOAc/heptane). M.p.: 224-225 °C. ^{1}H NMR (500 MHz, $(CD_3)_2SO$) δ 8.21 (s, 1H), 8.20 (s, 1H), 8.03 (d, J = 8.0 Hz, 1H), 8.02 (d, J = 1.6 Hz, 1H), 7.83 (d, J = 8.0 Hz, 1H), 7.66 (dd, J = 8.0, 1.9 Hz, 1H), 7.61 (d, J = 1.9 Hz, 1H), 7.45 (dd, J = 8.0, 1.6 Hz, 1H), 7.26 (d, J = 2.1 Hz, 1H), 7.24 (d, J = 2.1, 1H), 4.04 (t, J = 7.1 Hz, 2H), 1.76 – 1.69 (m, 2H), 1.43 (s, 9H), 1.34 (s, 9H), 1.30 – 1.25 (m, 6H), 0.87 (t, J = 7.1 Hz, 3H) ppm. ^{13}C NMR

(126 MHz, $(CD_3)_2SO$) δ 192.7, 155.12, 152.1, 150.3, 142.5, 141.8, 141.8, 137.4, 136.9, 134.6, 133.8, 131.8, 130.6, 123.0, 120.5, 120.4, 120.0, 119.7, 119.3, 116.8, 116.6, 115.8, 114.4, 114.1, 113.8, 50.3, 39.5, 34.9, 34.8, 31.5, 31.0, 30.9, 30.8, 25.6, 22.0, 13.9 ppm; five sp^2-C signals missing, presumably due to overlap. HRMS ($MALDI^+$, FT-ICR, dithranol) *m/z* = 604.2723 [M + H^+], calcd for ($C_{35}H_{41}NOS_2^+$) = 604.2702.

Compound 12

A solution of NaOMe was prepared from Na (182 mg, 7.92 mmol) and MeOH (3 mL) and stirred for 0.5 h. It was then added dropwise to a solution of **4** (251 mg, 0.372 mmol) in anhydrous THF (35 mL) and anhydrous MeOH (35 mL), resulting in a color change from orange to dark red. The reaction mixture was stirred for 1.5 h at rt before H_2O (50 mL) followed by aqueous HCl (1 M, 8 mL) were added. The resulting suspension was extracted with CH_2Cl_2 (200 mL), and the organic phase was washed with H_2O (3 × 120 mL), dried over $MgSO_4$, filtered, and concentrated under reduced pressure. The residue was filtered through a silica plug (SiO_2, CH_2Cl_2) and concentrated under reduced pressure, yielding **12** (168 mg, 87%) as golden dark red crystals**.** R_f = 0.32 (30% EtOAc/heptane). M.p.: 240 °C (decomp.). The compound decomposes in $CDCl_3$. ^1H NMR (500 MHz, $(CD_3)_2SO$) δ 11.68 (t, *J* = 2.8 Hz, 1H), 8.22 (s, 1H), 8.20 (s, 1H), 8.05 – 8.02 (m, 2H), 7.83 (d, *J* = 7.8 Hz, 1H), 7.65 (dd, *J* = 7.8, 2.0 Hz, 1H), 7.61 (d, *J* = 2.0 Hz, 1H), 7.45 (dd, *J* = 8.2, 1.7 Hz, 1H), 7.25 (dd, *J* = 2.8, 1.9 Hz, 1H), 7.21 (dd, *J* = 2.8, 1.9 Hz, 1H), 1.43 (s, 9H), 1.34 (s, 9H) ppm. ^{13}C NMR (126 MHz, $(CD_3)_2SO$) δ 192.7, 155.9, 152.1, 150.3, 142.5, 141.9, 141.8, 137.4, 136.9, 134.7, 133.8, 131.8, 130.6, 123.0, 120.4, 120.0, 119.9, 119.5, 117.2, 117.3, 115.8, 114.5, 111.2, 111.0, 34.9, 34.8, 31.5, 30.9 ppm; five sp^2-C signals missing, presumably due to overlap. HRMS ($MALDI^+$, FT-ICR, dithranol) *m/z* = 520.1760 [M + H^+], calcd for ($C_{33}H_{30}NOS_2^+$) = 520.1763.

Compound 14

A solution of **11** (100 mg, 0.166 mmol) in anhydrous toluene (6 mL) was added dropwise to an Ar-degassed solution of CBr_4 (264 mg, 0.796 mmol) and PPh_3 (406 mg, 1.55 mmol) in anhydrous toluene (10 mL). The reaction mixture was degassed with Ar for another 10 min before it was heated to reflux for 5 h, resulting in a color change from dark red to orange. Additional CBr_4 (221 mg, 0.666 mmol) and PPh_3 (402 mg, 1.53 mmol) were added, and the reaction mixture was heated to reflux for another 19 h before it was allowed to cool to rt and filtered. The filtrate was concentrated under reduced pressure, and the resulting orange/yellow solid was purified by flash column chromatography (SiO_2, 15% EtOAc/heptane). The resulting solid was triturated with heptane (4 × 2 mL) yielding **14** (72 mg, 57%) as an orange solid. The combined supernatants were concentrated under reduced pressure and the obtained orange oil solidified upon cooling in the freezer overnight. The solid was triturated with heptane (3 × 2 mL), yielding additional **14** (9 mg) as an orange solid (total yield: 81 mg, 64%). R_f = 0.30 (15% EtOAc/heptane). M.p.: 158 °C (decomp.). ^{1}H NMR (500 MHz, $CDCl_3$) δ 9.01 (s, 1H), 8.71 (d, *J* = 1.7 Hz, 1H), 8.27 (d, *J* = 1.3 Hz, 1H), 8.06 (s, 1H), 7.78 (d, *J* = 8.0 Hz, 1H), 7.75 (d, J = 7.9 Hz, 1H), 7.48 (dd, J = 8.0, 1.7 Hz, 1H), 7.38 (dd, *J* = 7.9, 1.3 Hz, 1H), 6.75 (s, 2H), 3.96 (t, *J* = 7.3 Hz, 2H), 1.82 (q, *J* = 7.1 Hz, 2H), 1.47 (s, 9H), 1.40 (s, 9H), 1.36 – 1.31 (m, 6H), 0.90 (t, *J* = 7.1, 3H) ppm. ^{13}C NMR (126 MHz, $CDCl_3$) δ 150.3, 150.3, 149.2, 139.9, 139.5, 138.9, 138.8, 138.1, 137.3, 135.8, 126.6, 123.2, 122.9, 122.2, 120.6, 118.9, 118.9, 118.8, 118.6, 117.2 114.3, 112.4, 112.3, 88.7, 51.3, 35.3, 35.3, 31.9, 31.7, 31.5, 26.5, 22.7, 14.2, 1.2 ppm; six sp^2-C signals missing, presumably due to overlap. HRMS ($MALDI^+$, FT-ICR, dithranol) *m/z* = 759.1092 [$M^{\cdot+}$], calcd for ($C_{40}H_{41}Br_2NS_2^{\cdot+}$) = 759.1021.

Compound 15

To a solution of **11** (80 mg, 0.132 mmol) in anhydrous toluene (20 mL) was added $TiCl_4$ (0.2 mL, 1.82 mmol) dropwise, resulting in a color change from dark red to black. Dropwise addition of diethyl malonate (0.2 mL, 1.32 mmol) and pyridine (0.3 mL, 3.72 mmol) resulted in another color change to dark red. The reaction mixture was stirred at rt for 20 h before additional $TiCl_4$ (0.2 mL, 1.82 mmol) and diethyl malonate (0.2 mL, 1.32 mmol) were added dropwise, and the reaction mixture was stirred for another 16 h and then filtered. The filtrate was diluted with toluene (150 mL), washed with brine (3 × 100 mL), dried over $MgSO_4$, filtered, and concentrated under reduced pressure. The resulting dark red oil was purified by flash column chromatography (SiO_2 neutralized with Et_3N, 35% EtOAc/heptane), yielding **15** (22 mg, 22%) as a deep red thin film after freeze-drying for five days. Minor impure fractions were combined and concentrated under reduced pressure. The obtained film was triturated with pentane (4 × 1 mL) to yield additional **15** (10 mg) as a deep red thin film (total yield: 32 mg, 32%). R_f = 0.30 (20% EtOAc/heptane). ^{1}H NMR (500 MHz, $(CD_3)_2CO$) δ 8.34 (s, 1H), 8.31 (s, 1H), 8.10 (d, *J* = 1.6 Hz, 1H), 7.95 (d, *J* = 1.7 Hz, 1H), 7.82 (d, *J* = 8.0 Hz, 1H), 7.79 (d, *J* = 8.0 Hz, 1H), 7.57 (dd, *J* = 8.0, 1.7 Hz, 1H), 7.46 (dd, *J* = 8.0, 1.6 Hz, 1H), 7.14 (s, 2H), 4.55 (q, *J* = 7.2 Hz, 2H), 4.48 (q, *J* = 7.2 Hz, 2H), 4.10 (t, *J* = 7.1 Hz, 2H), 1.85 (quin, *J* = 7.1 Hz, 2H), 1.46 (s, 9H), 1.44 – 1.38 (m, 6H), 1.37 (s, 9H), 1.36 – 1.25 (m, 6H), 0.88 (t, *J* = 5.0 Hz, 3H) ppm. ^{13}C NMR (126 MHz, $(CD_3)_2CO$) δ 166.4, 166.2, 152.8, 151.5, 151.2, 144.5, 141.4, 140.8, 140.5, 138.7, 138.0, 137.2, 135.8, 133.5, 129.2, 123.9, 123.2, 121.8, 121.1, 120.1, 119.7, 118.6, 118.4, 118.1, 115.0, 114.3, 114.1, 62.8, 62.7, 51.6, 35.8, 35.7, 32.3, 32.1, 32.1, 31.8, 29.8, 31.7, 27.0, 23.2, 14.4, 14.3 ppm; 5 sp^2 carbon signals missing, presumably due to overlap. HRMS (MALDI$^+$, FT-ICR, dithranol) *m/z* = 745.3493 [$M^{\cdot+}$], calcd for ($C_{46}H_{51}NO_4S_2^{\cdot+}$) = 745.3254.

Compound 17

To a flame-dried vial equipped with a magnetic stirrer bar were added **3** (70 mg, 212 μmol), **5** (24 mg, 135 μmol), and Lawesson's reagent (63 mg, 155 μmol). Dry toluene (5 mL) degassed with N_2 for 15 min was added, and the solution was heated to 105 °C for 18.5 h. The reaction mixture was then allowed to cool to rt, diluted with toluene (10 mL), and washed with 1 M NaOH (3 × 20 mL), and then with H_2O (20 mL). The organic phase was dried over $MgSO_4$ and concentrated under reduced pressure. The residue was purified by flash column chromatography twice (SiO_2, 1) 1% EtOAc/heptane, 2) 50% CH_2Cl_2/heptane), yielding **17** (8.6 mg, 18 μmol, 14%) as a yellow solid. $R_{f =}$ 0.18 (50% CH_2Cl_2/heptane). M.p.: 255 °C (decomp.). ^{1}H NMR (500 MHz, $CDCl_3$) δ 7.95 (d, *J* = 8.0 Hz, 2H), 7.80 (d, *J* = 7.2 Hz, 2H), 7.80 (d, *J* = 8.0 Hz, 2H), 7.40 (t, *J* = 7.7 Hz, 2H), 7.34 (t, *J* = 7.2, 2H), 7.33 (d, *J* = 7.7 Hz, 2H), 7.15 (s, 2H), 2.42 (s, 2H) ppm. ^{13}C NMR (126 MHz, $CDCl_3$) δ 148.2, 146.0 138.8, 137.5, 136.8, 135.6, 130.6, 127.4, 127.4, 126.5, 126.4, 123.9, 120.0, 111.5, 22.0 ppm. HRMS (MALDI$^+$, FT-ICR, dithranol) *m/z* = 459.0421 [$M^{\cdot+}$], calcd for ($C_{25}H_{17}NO_2S_3^{\cdot+}$) = 459.0416.

Compound 20

To a solution of 1-phenyl-2-trimethylsilylacetylene (0.10 mL, 0.517 mmol) in anhydrous THF (25 mL) and MeOH (25 mL) was added K_2CO_3 (0.286 g, 2.07 mmol). The reaction mixture was stirred at rt for 1 h until TLC analysis showed full conversion. It was then filtered through a plug of SiO_2 (CH_2Cl_2 as eluent) and concentrated under reduced pressure until the total volume was approx. 5 mL. Et_3N (10 mL) was added to the solution, and it was concentrated under reduced pressure until the total volume was approx. 5 mL (Et_3N). The freshly prepared phenylacetylene in Et_3N (approx. 5 mL) was then added to a flask along with **18** (108 mg, 0.124 mmol), anhydrous THF (18 mL), and Et_3N (7 mL), and the solution was degassed with Ar. P(*t*-Bu)$_3$ (0.14 mL, 1.0 M in toluene), Pd_2dba_3 (17 mg, 19 µmol), and CuI (4 mg, 19 µmol) were added, and the reaction mixture was stirred at rt overnight under an

Ar atmosphere. The dark brown/red reaction mixture was filtered through a plug of SiO_2 (CH_2Cl_2 as eluent) and purified by flash column chromatography (SiO_2 deactivated by 1% Et_3N, 10% CH_2Cl_2/heptane), yielding **20** as a red solid (44 mg, 0.048 mmol, 39%). R_f = 0.55 (50% CH_2Cl_2/heptane). ^{1}H NMR (500 MHz, CD_2Cl_2) δ 9.10 (d, *J* = 0.8 Hz, 1H), 8.82 (d, *J* = 1.7 Hz, 1H), 7.96 (d, *J* = 0.8 Hz, 1H), 7.81 – 7.78 (m, 2H), 7.77 (d, *J* = 1.5 Hz, 1H), 7.73 – 7.69 (m, 3H), 7.60 (d, *J* = 7.9 Hz, 1H), 7.56 – 7.51 (m, 3H), 7.47 – 7.44 (m, 4H), 7.33 (dd, *J* = 8.0, 1.6 Hz, 1H), 3.05 – 2.97 (m, 4H), 1.79 – 1.72 (m, 4H), 1.50 – 1.46 (m, 4H), 1.44 (s, 9H), 1.36 (s, 9H), 1.35 – 1.31 (m, 8H), 0.92 – 0.88 (m, 6H) ppm. *Another ^{1}H NMR spectrum measured in C_6D_6 to disrupt π-stacking:* ^{1}H NMR (500 MHz, C_6D_6) δ 9.59 (s, 1H), 9.20 (d, *J* = 1.7 Hz, 1H), 8.32 (s, 1H), 8.06 (d, *J* = 1.6 Hz, 1H), 7.82 (d, *J* = 7.9 Hz, 1H), 7.65 – 7.59 (m, 4H), 7.34 (dd, *J* = 7.9, 1.8 Hz, 1H), 7.28 (dd, *J* = 8.0, 1.6 Hz, 1H), 7.09 – 6.99 (m, 7H), 2.75 – 2.69 (m, 4H), 1.61 – 1.54 (m, 4H), 1.42 (s, 9H), 1.32 (s, 9H), 1.29 – 1.19 (m, 8H), 1.17 – 1.11 (m, 4H), 0.88 – 0.82 (m, 6H) ppm. ^{13}C NMR (126 MHz, CD_2Cl_2) δ 151.2, 150.6, 146.2, 139.8, 139.2, 138.9, 138.5, 138.5, 137.8, 137.6, 135.9, 135.6, 132.2, 132.2, 129.8, 129.6, 129.5, 129.2, 129.0, 128.8, 127.3, 123.6, 123.4, 123.1, 122.9, 121.6, 120.3, 119.2, 117.3, 114.3, 99.9, 98.6, 98.2, 90.0, 89.2, 37.1, 37.0, 35.4, 35.4, 31.9, 31.8, 31.8, 31.8, 30.4, 30.3, 28.7, 23.0, 23.0, 14.2, 14.2 ppm; one sp^2-C signal and one sp^3-C signal missing, presumably due to overlap. *Another ^{13}C NMR spectrum measured in C_6D_6 to disrupt π-stacking could not be obtained due to low concentration of the measured sample.* HRMS (MALDI$^+$, FT-ICR, dithranol) *m/z* = 910.3749 [$M^{\cdot+}$], calcd for ($C_{60}H_{62}S_4^{\cdot+}$) = 910.3729.

Compound 21

To a solution of 4-[(trimethylsilyl)ethynyl]benzonitrile (0.319 g, 1.6 mmol) in anhydrous THF (25 mL) and MeOH (25 ml) was added K_2CO_3 (0.885 g, 6.4 mmol). The reaction mixture was stirred at rt for 2 h until TLC analysis showed full conversion. It was then filtered through a plug of SiO_2 (CH_2Cl_2 as eluent) and concentrated under reduced pressure until the total

volume was approx. 5 mL. Et_3N (10 mL) was added to the solution, and it was concentrated under reduced pressure until the total volume was approx. 5 mL (Et_3N). The freshly prepared 4-ethynylbenzonitrile in Et_3N (approx. 5 mL) was then added to a flask along with **18** (185 mg, 0.21 mmol) and anhydrous THF (15 mL), and the solution was degassed vigorously with Ar. $Pd(PPh_3)_2Cl_2$ (7 mg, 0.01 mmol) and CuI (2 mg, 0.01 mmol) were added, and the reaction mixture was stirred at 45–50 °C overnight under a N_2 atmosphere. The dark brown/red reaction mixture was filtered through a plug of SiO_2 (CH_2Cl_2 as eluent) and purified by flash column chromatography (SiO_2, 50% CH_2Cl_2/heptane), yielding **21** as a dark red solid (45 mg, 0.05 mmol, 22%). R_f = 0.29 (100% toluene). ^{1}H NMR (500 MHz, $CDCl_3$) δ 8.87 (s, 1H), 8.60 (d, J = 1.7 Hz, 1H), 7.87 (s, 1H), 7.78 – 7.63 (m, 9H), 7.61 (d, J = 8.0 Hz, 1H), 7.43 (d, J = 8.0 Hz, 1H), 7.38 (dd, J = 7.9, 1.8 Hz, 1H), 7.27 (dd, J = 8.0, 1.6 Hz, 1H), 2.96 – 2.89 (m, 4H), 1.72 – 1.65 (m, 4H), 1.44 – 1.41 (m, 3H), 1.38 (s, 9H), 1.35 – 1.27 (m, 9H), 1.27 (s, 9H), 0.86 – 0.81 (m, 6H) ppm. A ^{13}C NMR spectrum could not be obtained due to low concentration of the measured sample. HRMS (MALDI$^+$, FT-ICR, dithranol) m/z = 960.3652 [$M^{\cdot+}$], calcd for ($C_{62}H_{60}N_2S_4^{\cdot+}$) = 960.3634.

Compound 22

In a manner similar to [1], K_2CO_3 (180 mg, 1.30 mmol) was added to a solution of triisopropyl((2-((trimethylsilyl)ethynyl)phenyl)ethynyl)silane (220 mg, 0.620 mmol) in THF (10 mL) and MeOH (10 mL), and the suspension was stirred at rt for 1 h before it was filtered through a plug of SiO_2 (CH_2Cl_2 as eluent) and concentrated in vacuum to a volume of approx. 10 mL. Et_3N (10 mL) was added, and the solution was further concentrated to a volume of approx. 2 mL. Additional Et_3N (10 mL), anhydrous THF (10 mL), and **18** (102 mg, 0.144 mmol) were added, and the combined solution was thoroughly degassed with Ar prior to addition of $Pd(PPh_3)_2Cl_2$ (20 mg, 0.028 mmol) and CuI (5.0 mg, 0.026 mmol). The resulting reaction mixture was stirred at rt under an Ar atmosphere for 14 h before it was

filtered through a plug of SiO_2 (CH_2Cl_2 as eluent) and concentrated under reduced pressure. Flash column chromatography (SiO_2,10% CH_2Cl_2/heptane) yielded **22** (65 mg, 44%) as a red oil. R_f = 0.35 (20% CH_2Cl_2/heptane). ^{1}H NMR (500 MHz, $CDCl_3$) δ 9.12 (s, 1H), 8.80 (d, J = 1.7 Hz, 1H), 7.95 (s, 1H), 7.75 – 7.71 (m, 2H), 7.68 (d, J = 8.0 Hz, 1H), 7.66 – 7.60 (m, 2H), 7.58 – 7.54 (m, 1H), 7.53 (d, J = 8.0 Hz, 1H), 7.43 – 7.38 (m, 3H), 7.35 – 7.30 (m, 2H), 7.26 – 7.21 (m, 1H), 3.02 – 2.97 (m, 4H), 1.86 – 1.68 (m, 4H), 1.58 – 1.44 (m, 4H), 1.43 (s, 9H), 1.37 – 1.31 (m, 8H), 1.24 (s, 9H), 0.98 (s, 18H), 0.95 (s, 18H), 0.93 – 0.87 (m, 6H) ppm. ^{13}C NMR (126 MHz, $CDCl_3$) δ 150.6, 150.0, 147.2, 139.6, 138.5, 138.5, 138.3, 137.6, 137.5, 137.3, 136.1, 135.8, 133.3, 133.0, 132.8, 132.3, 129.3, 128.6, 128.4, 128.2, 127.9, 126.8, 126.7, 126.3, 125.9, 123.3, 123.1, 122.0, 120.0, 119.4, 118.7, 117.5, 113.9, 105.1, 105.0, 100.1, 96.5, 96.5, 96.3, 96.1, 92.9, 92.2, 77.4, 36.9, 36.8, 35.2, 35.1, 31.9, 31.6, 31.5, 31.5, 30.1, 30.0, 28.5, 28.5, 22.7, 22.7, 18.7, 18.7, 14.2, 14.2, 11.4 ppm; one signal missing in the aromatic region and one signal missing in the aliphatic region, presumably due to overlap. HRMS (MALDI$^+$, FT-ICR, dithranol) m/z = 1270.6417 [$M^{\cdot+}$], calcd for ($C_{82}H_{102}S_4Si_2^{\cdot+}$) = 1270.6397.

Compound 24

To a N_2-degassed solution of **1** (56 mg, 0.14 mmol) in anhydrous toluene (20 mL) was added CBr_4 (191 mg, 0.576 mmol) and PPh_3 (300 mg, 1.14 mmol). The suspension was heated to reflux and stirred under a N_2 atmosphere for 4 h before it was cooled to rt, filtered through a plug of SiO_2 (CH_2Cl_2 as eluent), and concentrated under reduced pressure. Flash column chromatography (10% CH_2Cl_2/heptane) yielded **24** (29 mg, 37%) as an orange solid. R_f = 0.29 (20% CH_2Cl_2/heptane). ^{1}H NMR (500 MHz, $CDCl_3$) δ 8.72 (d, J = 0.7 Hz, 1H), 8.69 (d, J = 1.7 Hz, 1H), 7.88 (d, J = 0.7 Hz, 1H), 7.72 (d, J = 1.7 Hz, 1H), 7.61 (d, J = 8.0 Hz, 1H), 7.53 – 7.49 (m, 2H), 7.46 (d, J = 8.0 Hz, 1H), 1.39 (s, 9H), 1.36 (s, 9H) ppm. ^{13}C NMR (126

MHz, $CDCl_3$) δ 194.0, 152.9, 151.4, 144.0, 143.6, 142.2, 141.6, 139.6, 138.2, 137.1, 135.5, 134.9, 131.9, 127.2, 123.2, 121.8, 119.9, 119.4, 117.7, 115.2, 93.3, 35.4, 35.2, 31.7, 31.4 ppm. HRMS (MALDI$^+$, FT-ICR, dithranol) *m/z* = 550.0371 [$M^{\cdot+}$], calcd for ($C_{29}H_{26}Br_2O^{\cdot+}$) = 550.0325.

Compound 25

To a N_2-degassed solution of **1** (250 mg, 0.633 mmol) in anhydrous toluene (50 mL) were added CBr_4 (900 mg, 2.71 mmol) and PPh_3 (1.40 mg, 5.34 mmol). The suspension was heated to reflux and stirred under a N_2 atmosphere for 2 h before it was cooled to rt, filtered through a plug of SiO_2 (CH_2Cl_2 as eluent) and concentrated under reduced pressure. The crude material was re-dissolved in a minimum of CH_2Cl_2 (approx. 5 mL) before addition of MeOH (20 mL) led to precipitation of a yellow solid. Trituration of the solids with MeOH (3 × 10 mL) yielded **25** (314 mg, 70%) as a yellow solid. R_f = 0.21 (10% CH_2Cl_2/heptane). ^{1}H NMR (500 MHz, $CDCl_3$) δ 8.83 (s, 2H), 8.69 (s, 2H), 7.61 (d, *J* = 7.9 Hz, 2H), 7.47 (d, *J* = 7.9 Hz, 2H), 1.39 (s, 18H) ppm. ^{13}C NMR (126 MHz, $CDCl_3$) δ 150.8, 139.8, 139.6, 139.4, 138.4, 138.1, 126.8, 123.2, 119.0, 117.0, 91.0, 35.3, 31.7 ppm. HRMS (MALDI$^+$, FT-ICR, dithranol) *m/z* = 705.8756 [$M^{\cdot+}$], calcd for ($C_{30}H_{26}Br_4^{\cdot+}$) = 705.8722.

Compound 26

To a N_2-degassed solution of **25** (208 mg, 0.295 mmol) in THF (13 mL) and Et_3N (13 mL) were added Ar-degassed triisopropylsilylacetylene (1.85 mL, 1.50 g, 8.26 mmol), $Pd(PPh_3)Cl_2$ (0.0586 g, 0.0835 mmol), and CuI (0.0161 g, 0.0845 mmol). The reaction mixture was stirred for 25 h at rt under a N_2 atmosphere before it was filtered through a plug of SiO_2 (CH_2Cl_2 as eluent) and concentrated under reduced pressure. The orange residue was purified by flash column chromatography (SiO_2, 10% CH_2Cl_2/heptane), yielding **26** as red crystals (229 mg, 0.206 mmol, 70%). R_f = 0.58 (10% CH_2Cl_2/heptane). ^{1}H NMR (500

MHz, $CDCl_3$) δ 8.91 (m, 2H), 8.77 (m, 2H), 7.55 – 7.53 (d, *J* = 8 Hz, 2H), 7.34 – 7.32 (d, *J* = 8 Hz, 2H), 1.38 (s, 18H) ppm. ^{13}C NMR (126 MHz, $CDCl_3$) δ 150.7, 145.6, 139.9, 139.5, 138.2, 138.1, 126.5, 123.1, 118.9, 116.9, 106.6, 106.5, 103.5, 102.7, 101.4, 35.2, 31.8, 19.0, 11.7 ppm. HRMS (MALDI$^+$, FT-ICR, dithranol) *m/z* = 1111.7786 [M + H$^+$], calcd for ($C_{74}H_{111}Si_4^+$) = 1111.7757. Elemental analysis: C: 79.90%, H: 10:30%; calcd for $C_{74}H_{110}Si_4$: C: 79.93%, H: 9.97%.

Compound 27

In a manner similar to [1], K_2CO_3 (300 mg, 2.17 mmol) was added to a solution of triisopropyl((2-((trimethylsilyl)ethynyl)phenyl)ethynyl)silane (376 mg, 1.06 mmol) in THF (10 mL) and MeOH (10 mL). The suspension was stirred at rt for 45 min before it was filtered through a plug of SiO_2 (CH_2Cl_2 as eluent) and concentrated under reduced pressure to a volume of approx. 10 mL. Et_3N (10 mL) was added, and the solution was further concentrated to a volume of approx. 2 mL. Additional Et_3N (10 mL), anhydrous THF (10 mL), and **25** (150 mg, 0.212 mmol) were added, and the combined solution was thoroughly degassed with Ar before addition of $Pd(PPh_3)_2Cl_2$ (30 mg, 0.043 mmol) and CuI (8.0 mg, 0.042 mmol). The resulting reaction mixture was stirred at rt under an Ar atmosphere for 22 h before it was filtered through a plug of SiO_2 (CH_2Cl_2 as eluent) and concentrated under reduced pressure. Flash column chromatography (10% CH_2Cl_2/heptane) yielded **27** (75 mg, 23%) as an orange solid. R_f = 0.31 (20% CH_2Cl_2/heptane). ^1H NMR (500 MHz, $CDCl_3$) δ 8.96 (s, 2H), 8.74 (d, *J* = 1.7 Hz, 2H), 7.74 – 7.67 (m, 2H), 7.65 – 7.58 (m, 4H), 7.57 – 7.52 (m, 2H), 7.43 – 7.38 (m, 4H), 7.36 (d, *J* = 8.0 Hz, 2H), 7.34 – 7.30 (m, 4H), 7.24 (dd, *J* = 8.0, 1.7 Hz, 2H), 1.20 (s, 18H), 0.98 (s, 36H), 0.97 (s, 36H) ppm. ^{13}C NMR (126 MHz, $CDCl_3$) δ 150.5, 147.0, 139.9, 139.2, 138.1, 138.1, 133.3, 132.9, 132.8, 132.3, 128.7, 128.5, 128.2, 127.9, 127.0, 126.6, 126.3, 125.8, 125.8, 122.9, 119.0, 117.0, 105.0, 105.0, 101.1, 96.9,

96.9, 96.4, 96.2, 92.6, 92.0, 35.1, 31.5, 18.7, 18.7, 11.4, 11.4 ppm. HRMS (MALDI$^+$, FT-ICR, dithranol) *m/z* = 1512.9086 [$M^{\cdot+}$], calcd for ($C_{106}H_{127}Si_4^{\cdot+}$) = 1512.9043.

UV–vis absorption studies of compounds 10 and 11 (different solvents)

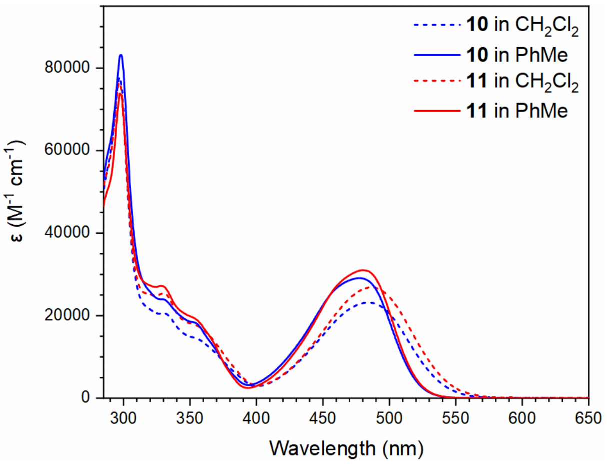

Figure S1: UV–vis absorption spectra of compounds **10** and **11** in PhMe and CH_2Cl_2 at 25 °C. The redshift of the longest-wavelength absorption when changing the solvent to CH_2Cl_2 indicates some charge-transfer character of this absorption.

UV–vis absorption studies of 20 (degradation studies)

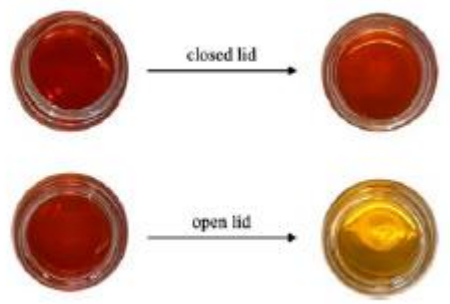

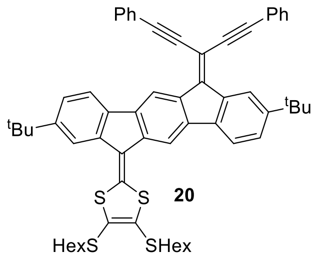

20

Figure S2: Visual experiment of compound **20** dissolved in CH_2Cl_2 in two vials, one with closed lid (top) and one with open lid (bottom), to observe the impact of the presence of oxygen. The solutions were not shielded from light.

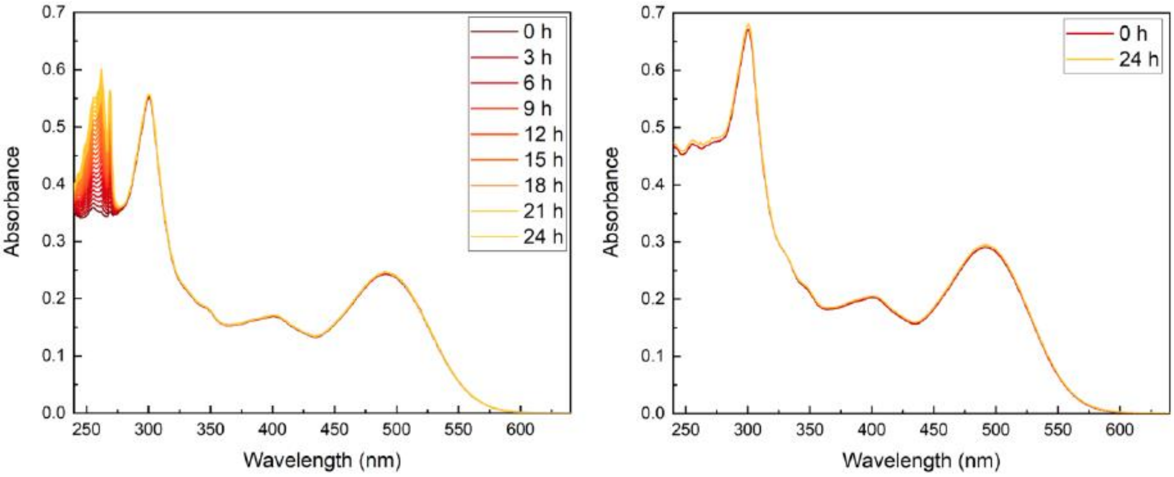

Figure S3: UV–vis absorption spectra of **20** in CH_2Cl_2 at 25 °C. Left: Recorded for 0–24 h in the presence of oxygen and absence of light; the absorption from 280 nm to 600 nm remains unchanged; however, an increase was observed in the absorption between 240 nm and 280 nm. Right: Recorded at 0 h and 24 h in the absence of both oxygen (sample degassed with argon) and light; no changes in the absorption were observed, indicating that the sample was stable in the absence of oxygen and light.

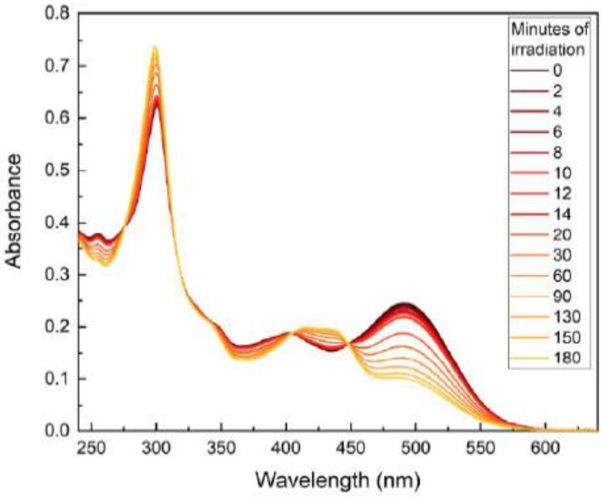

Figure S4: UV–vis absorption spectra of **20** in CH_2Cl_2 at 25 °C irradiated at 565 nm for 0–180 min in the presence of oxygen; a decrease in the absorption was observed between 475 nm and 575 nm, while an increase in the absorption was observed between 400 nm and 450 nm. These changes in absorption might explain the change in color observed for the samples in Figure S2.

^{1}H and ^{13}C NMR spectra

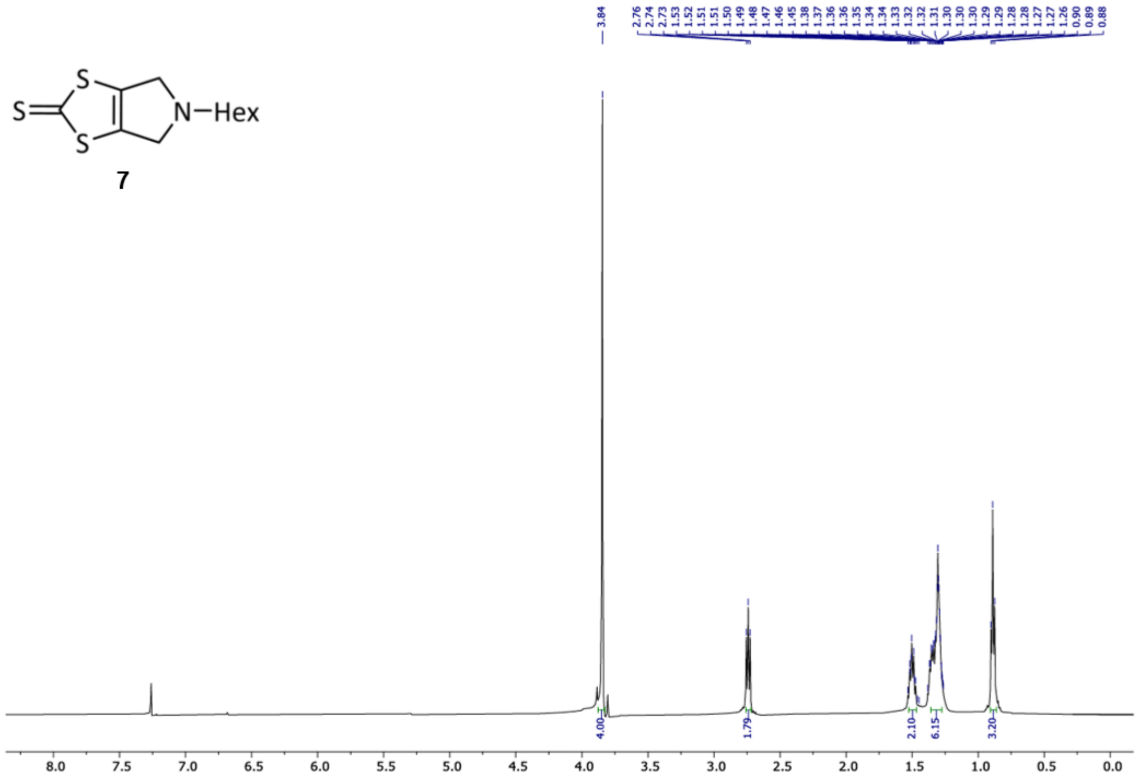

^{1}H NMR spectrum (500 MHz, $CDCl_3$) of **7**.

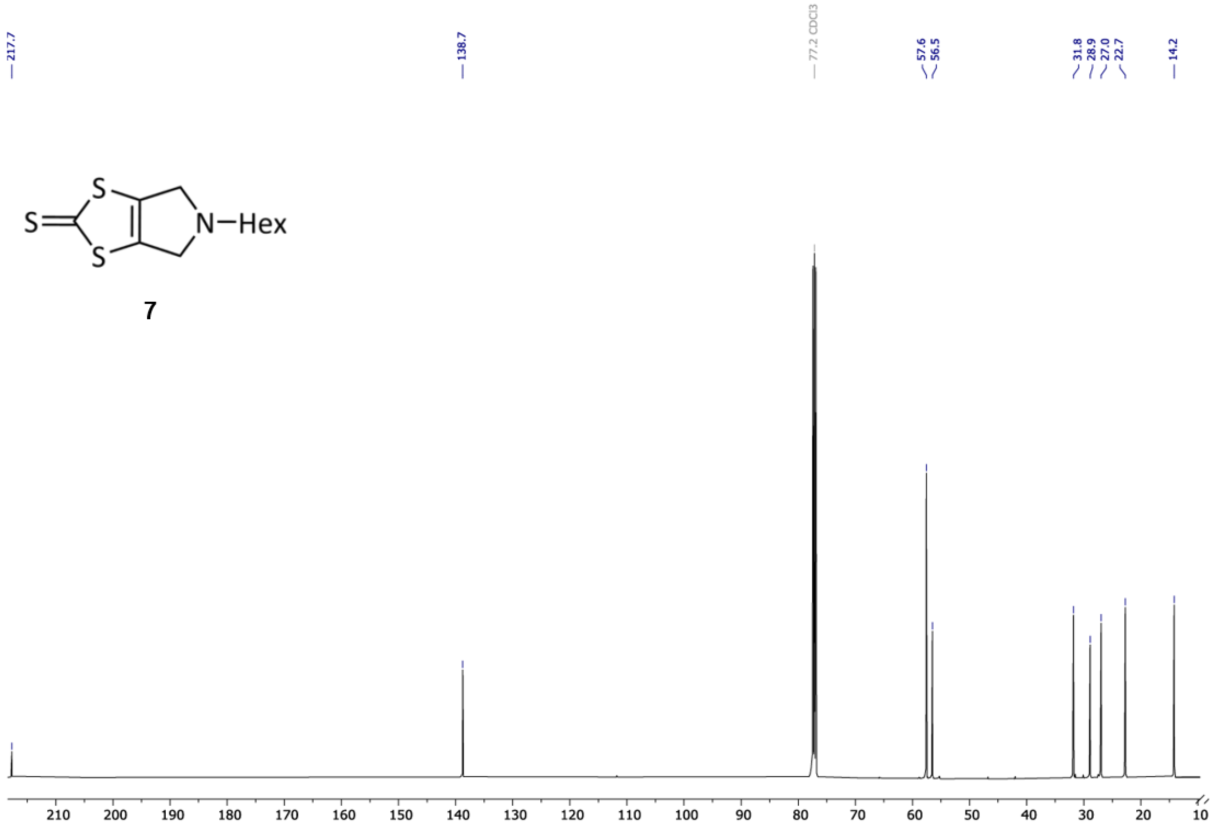

^{13}C NMR spectrum (126 MHz, $CDCl_3$) of **7**.

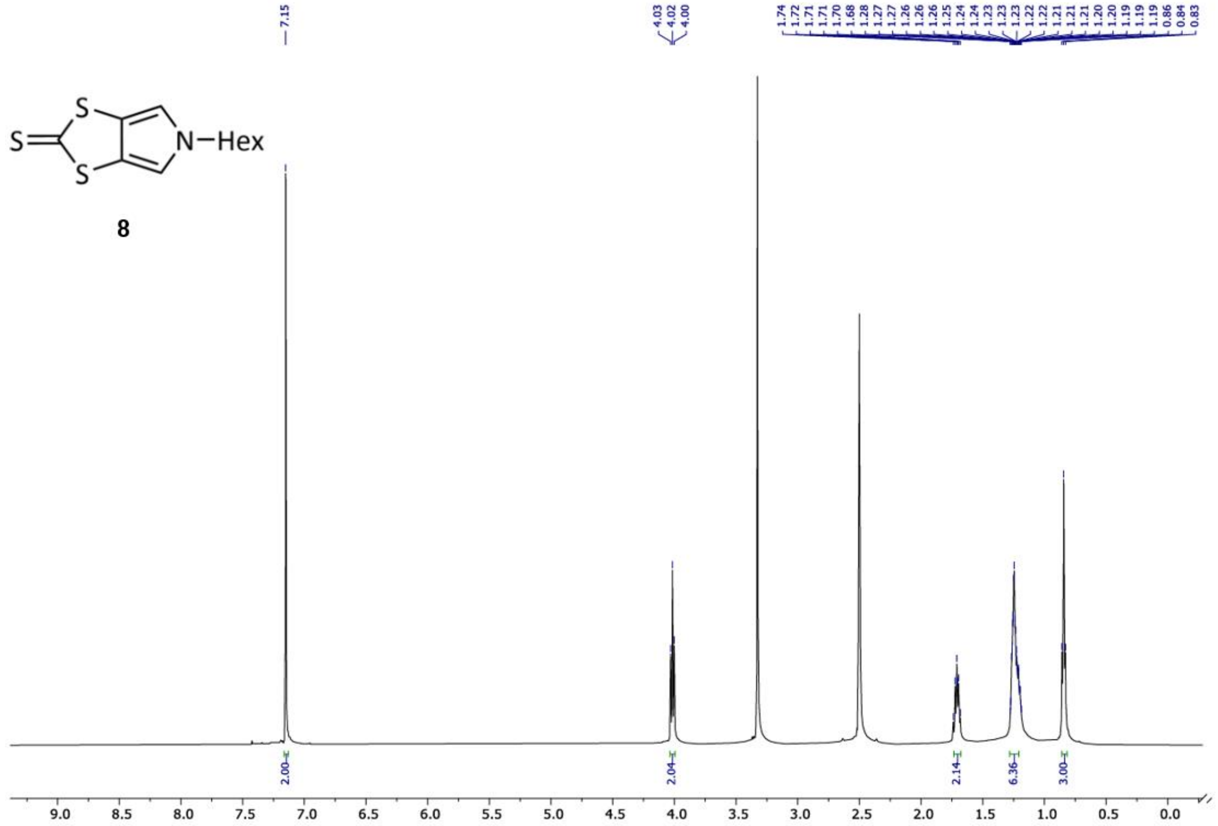

^1H NMR spectrum (500 MHz, $(CD_3)_2SO$) of **8**.

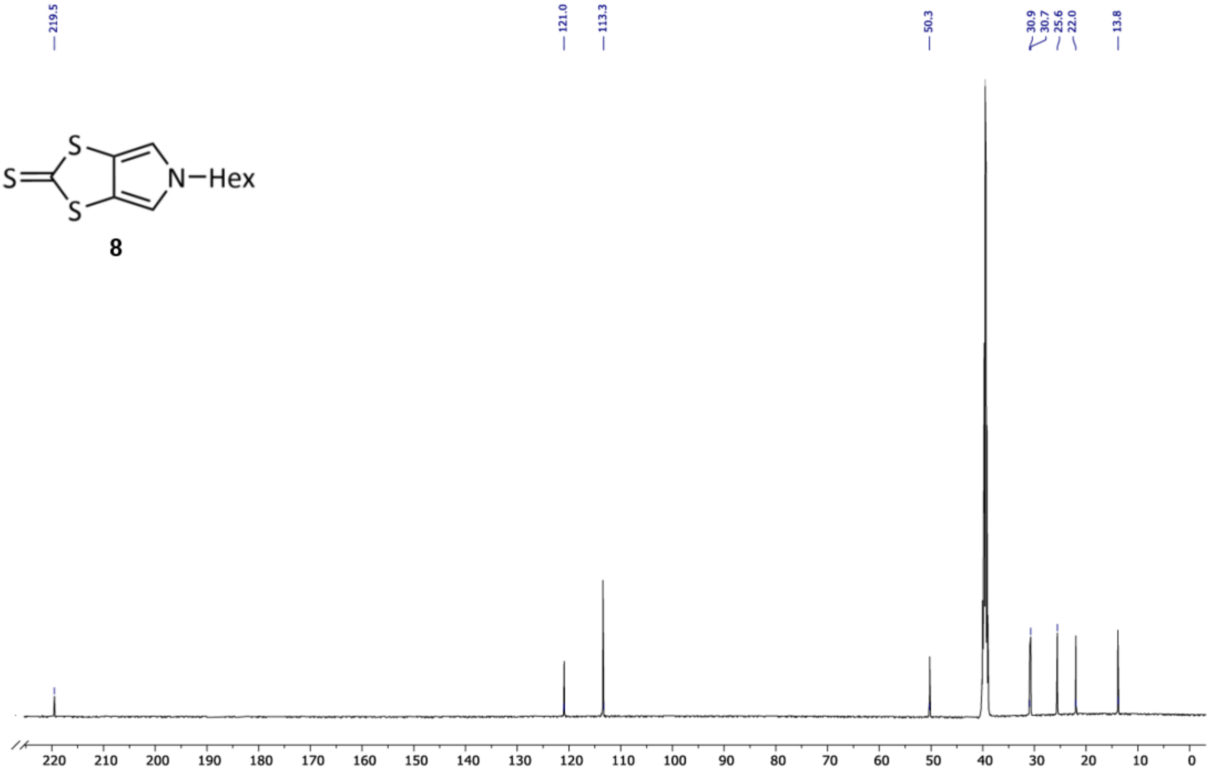

^{13}C NMR spectrum (126 MHz, $(CD_3)_2SO$) of **8**.

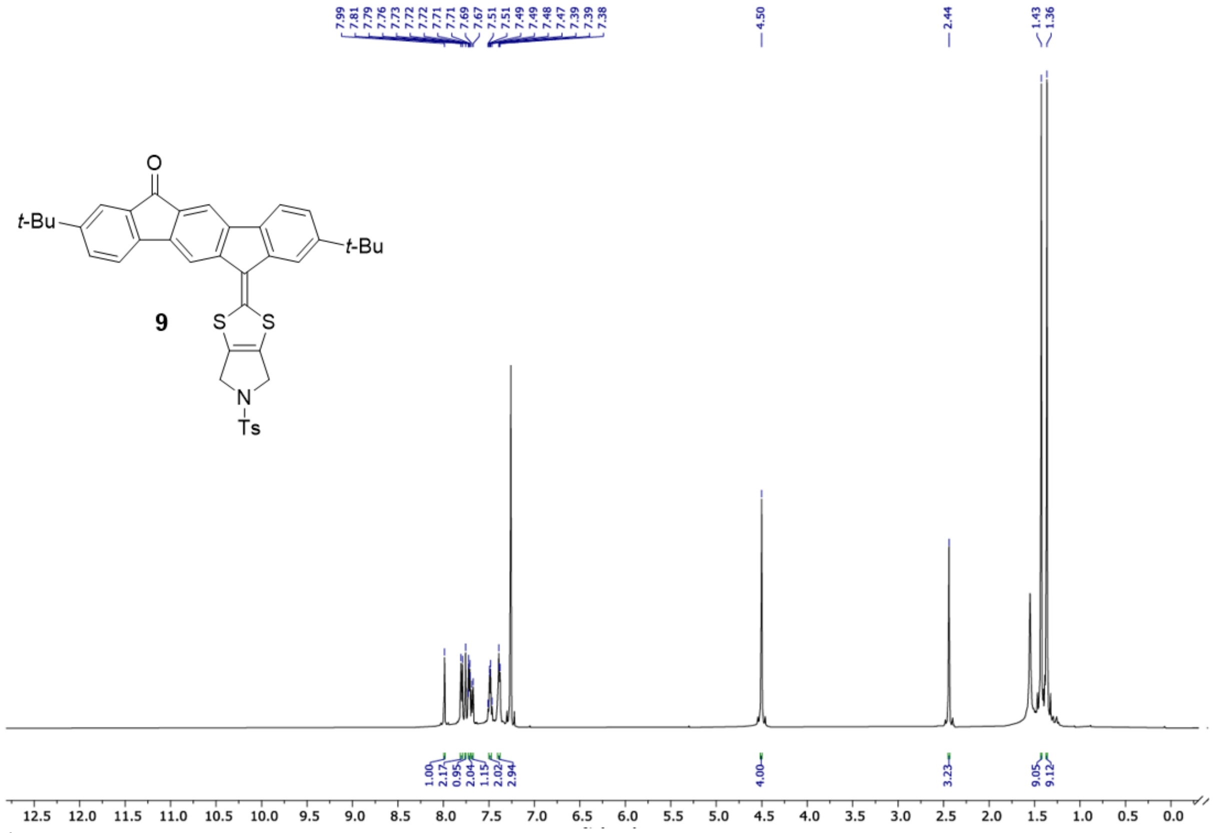

^{1}H NMR spectrum (500 MHz, $CDCl_3$) of **9**.

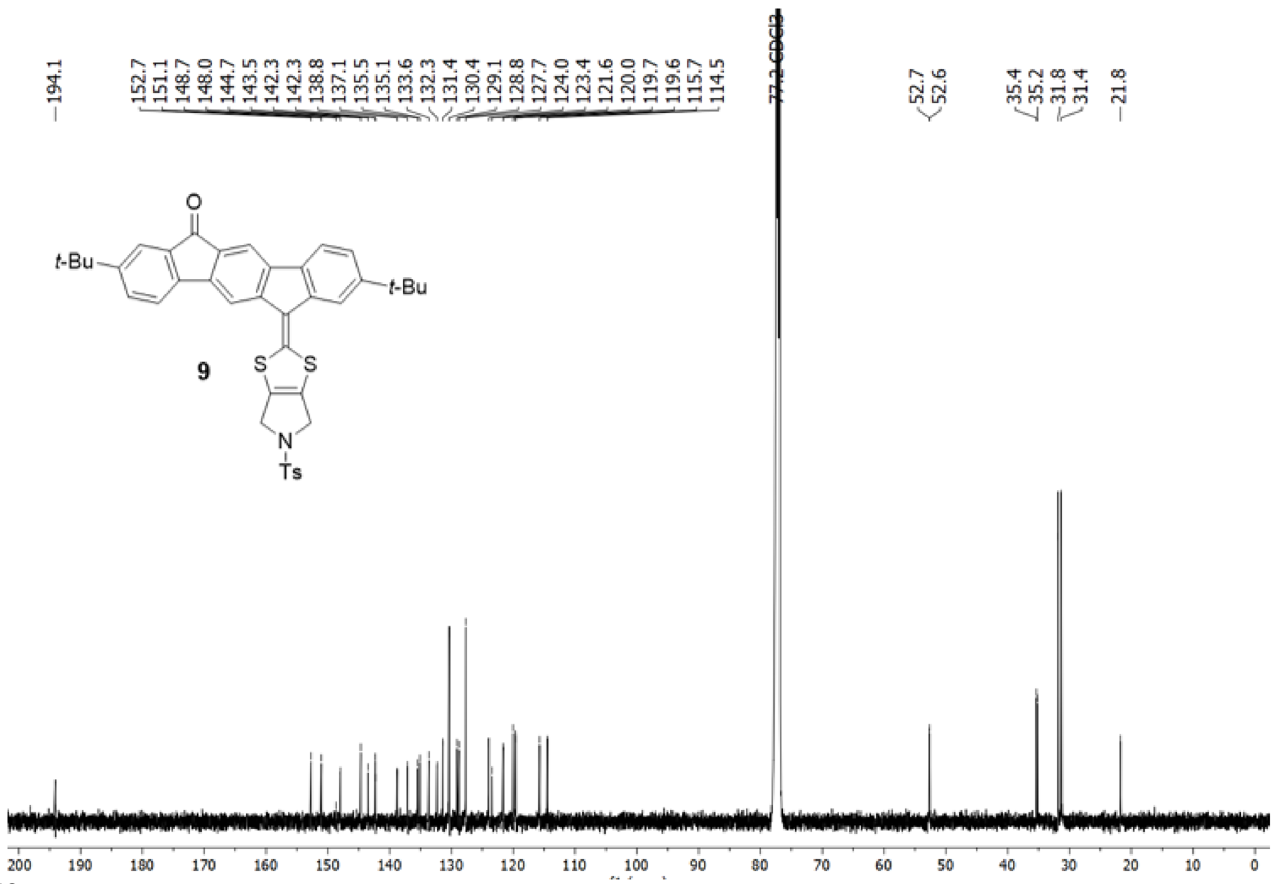

^{13}C NMR spectrum (126 MHz, $CDCl_3$) of **9**.

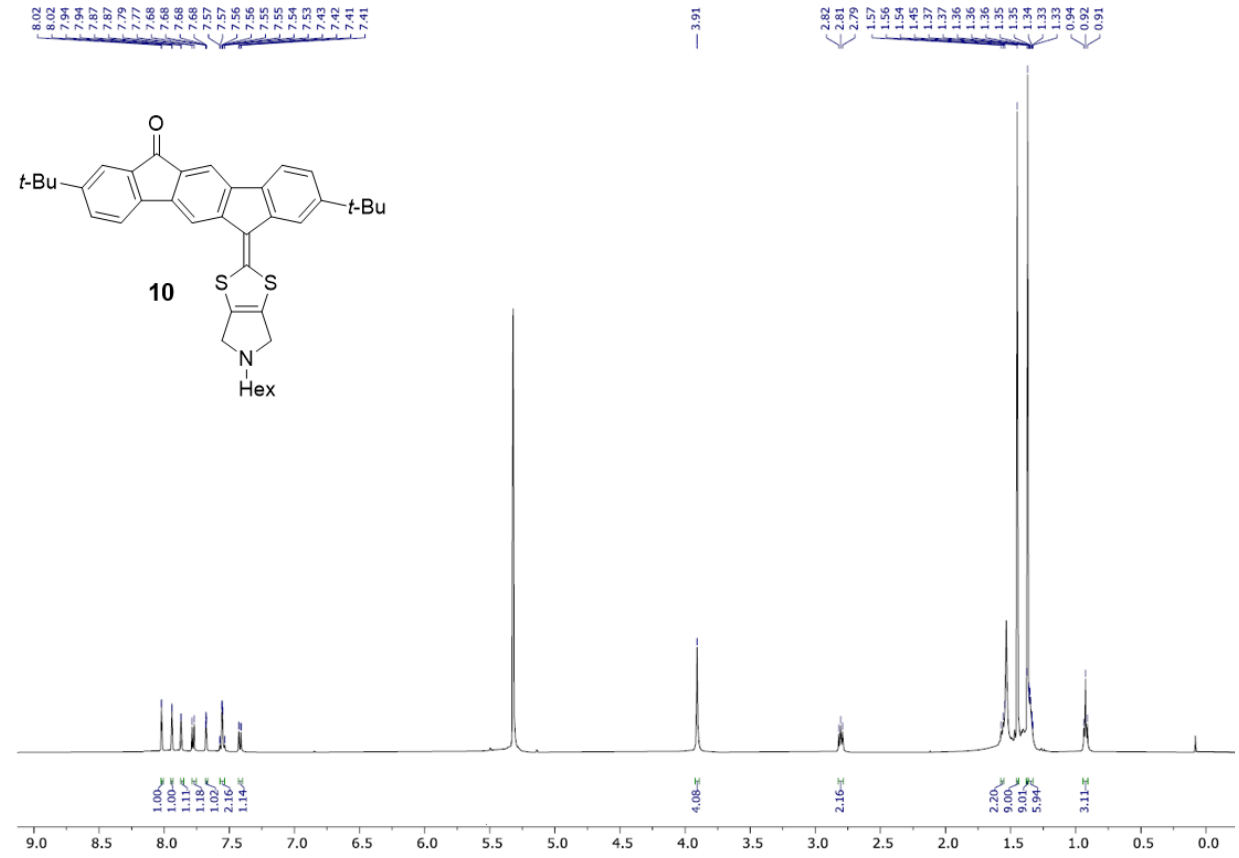

^{1}H NMR spectrum (500 MHz, CD_2Cl_2) of **10**.

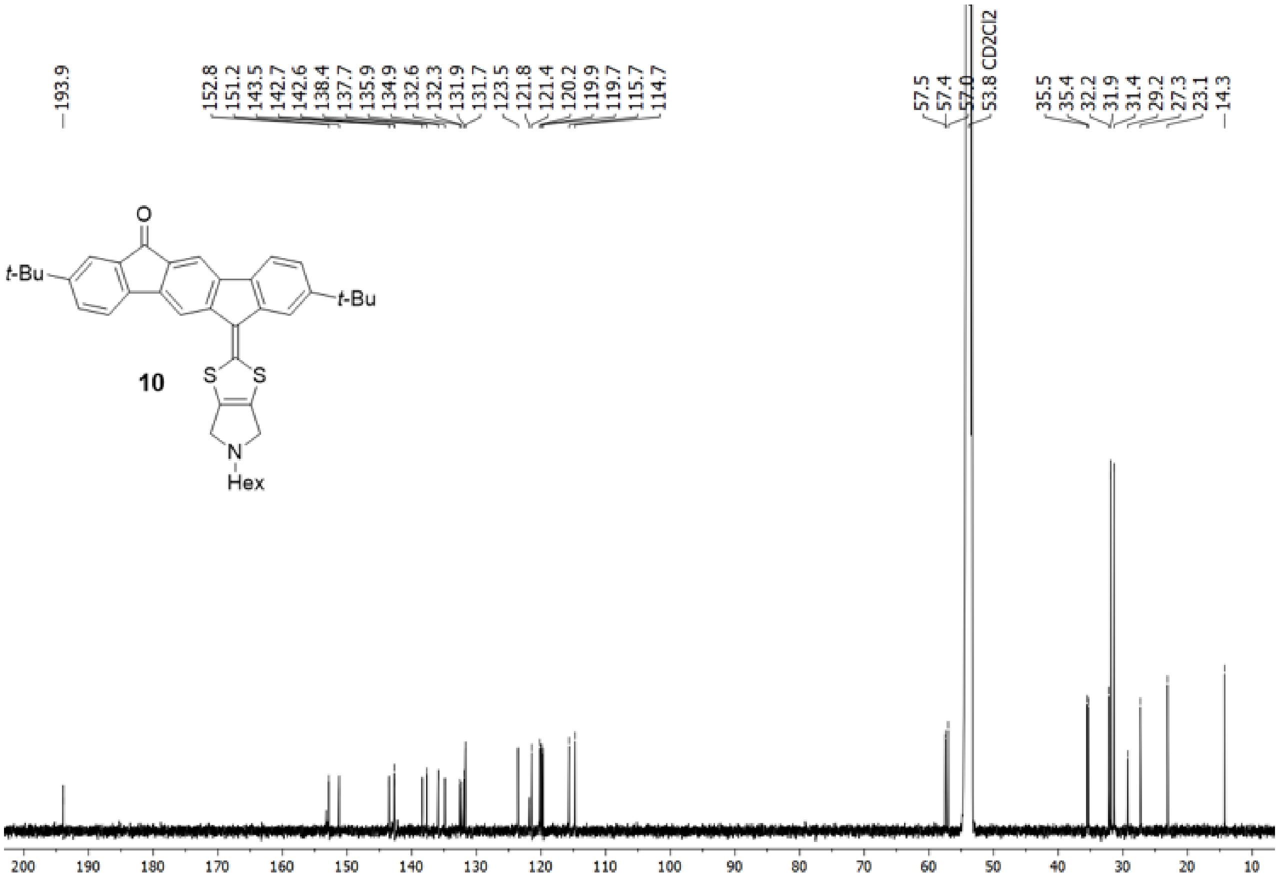

^{13}C NMR spectrum (126 MHz, CD_2Cl_2) of **10**.

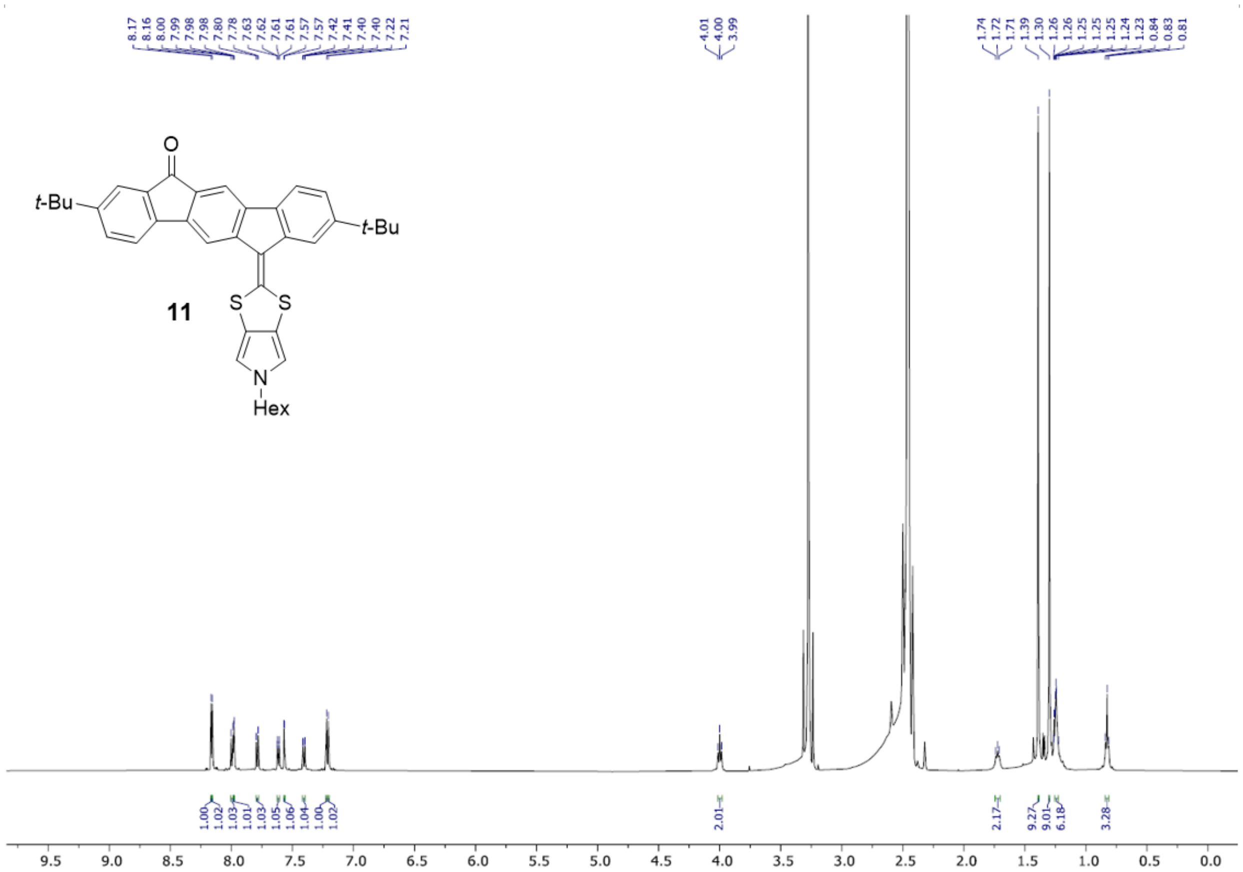

^{1}H NMR spectrum (500 MHz, $(CD_3)_2SO$) of **11**.

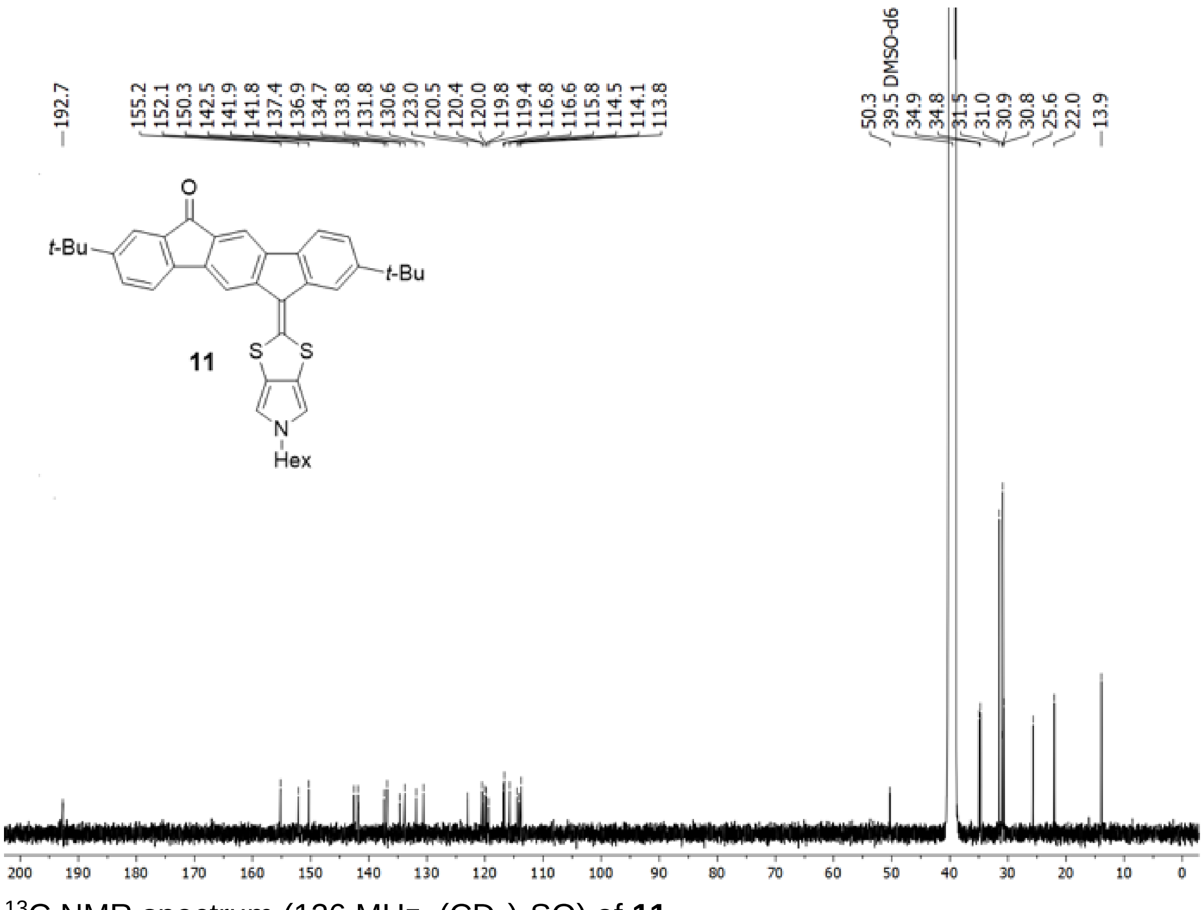

^{13}C NMR spectrum (126 MHz, $(CD_3)_2SO$) of **11**.

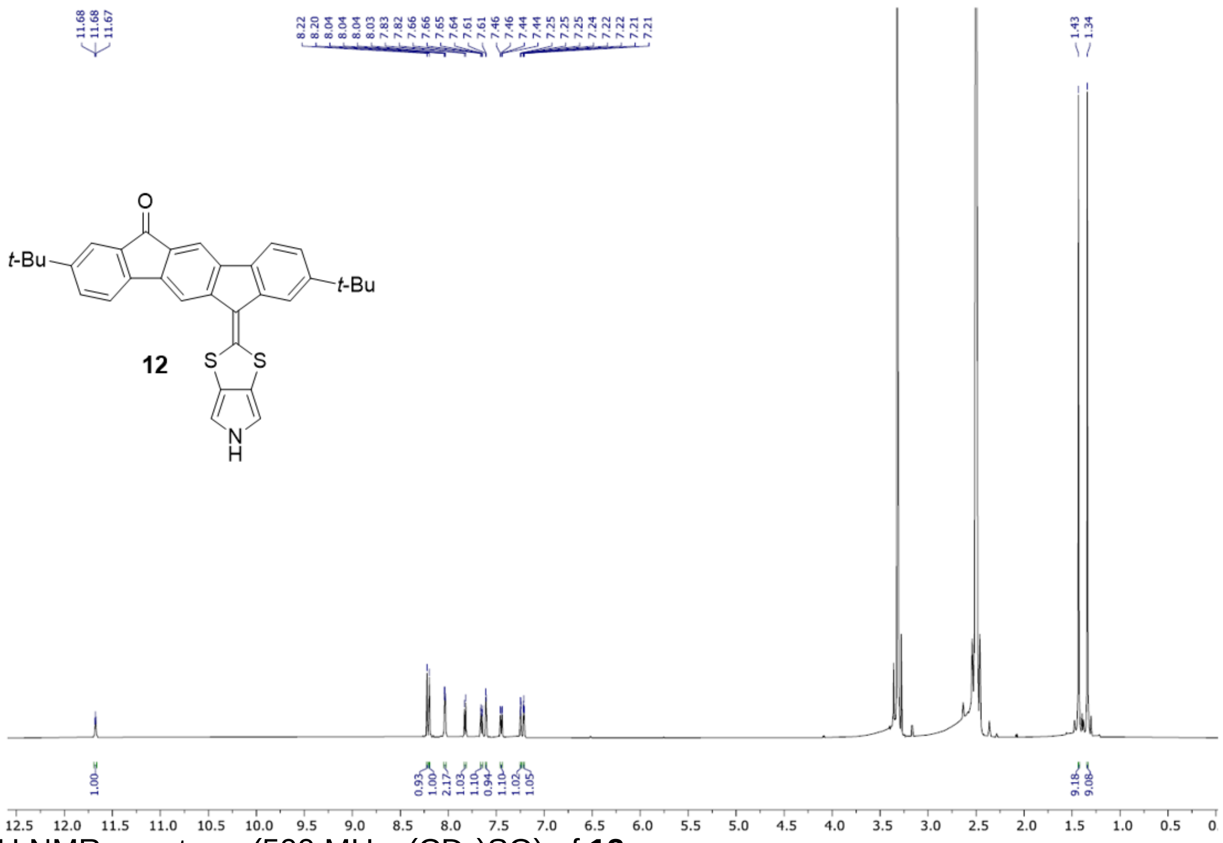

^{1}H NMR spectrum (500 MHz, $(CD_3)SO$) of **12**.

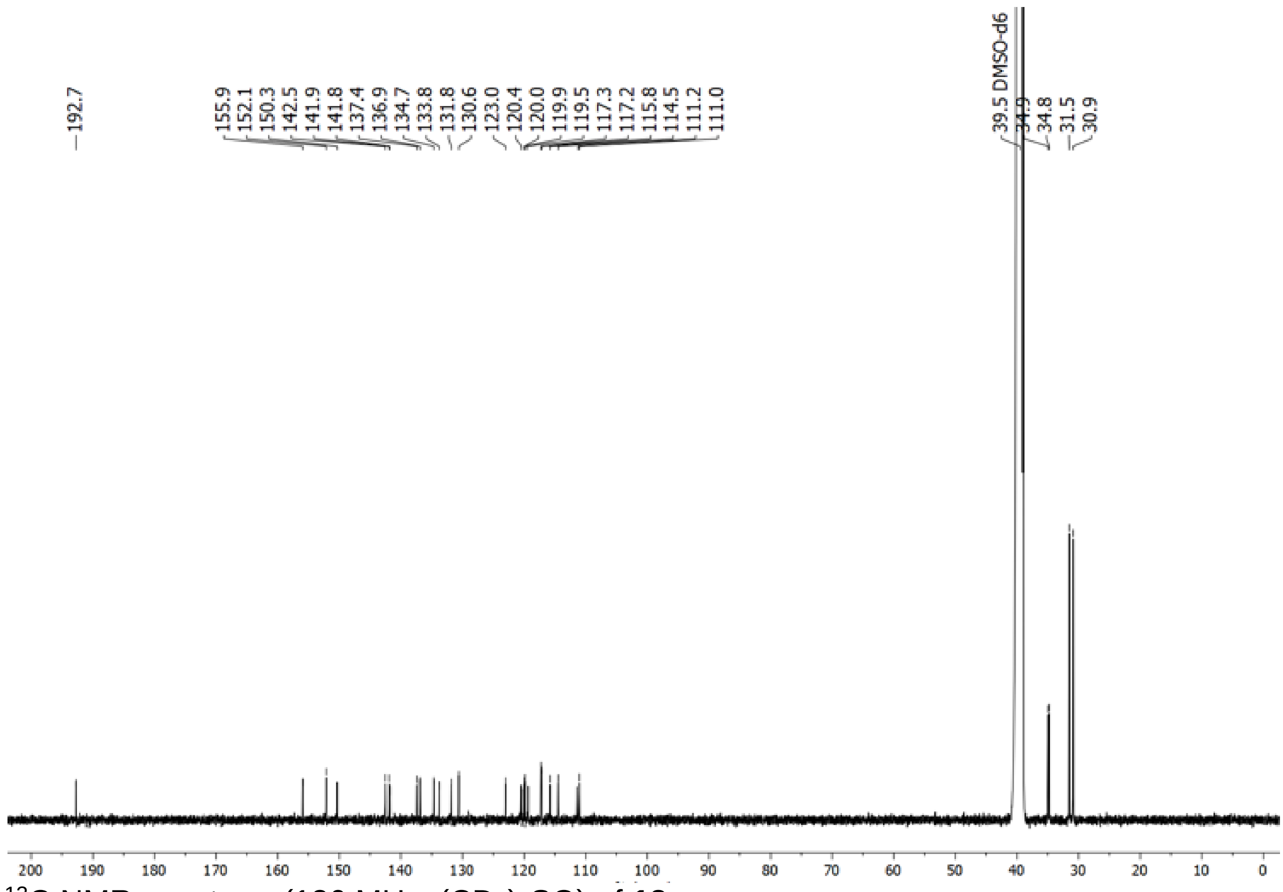

^{13}C NMR spectrum (126 MHz, $(CD_3)_2SO$) of **12**.

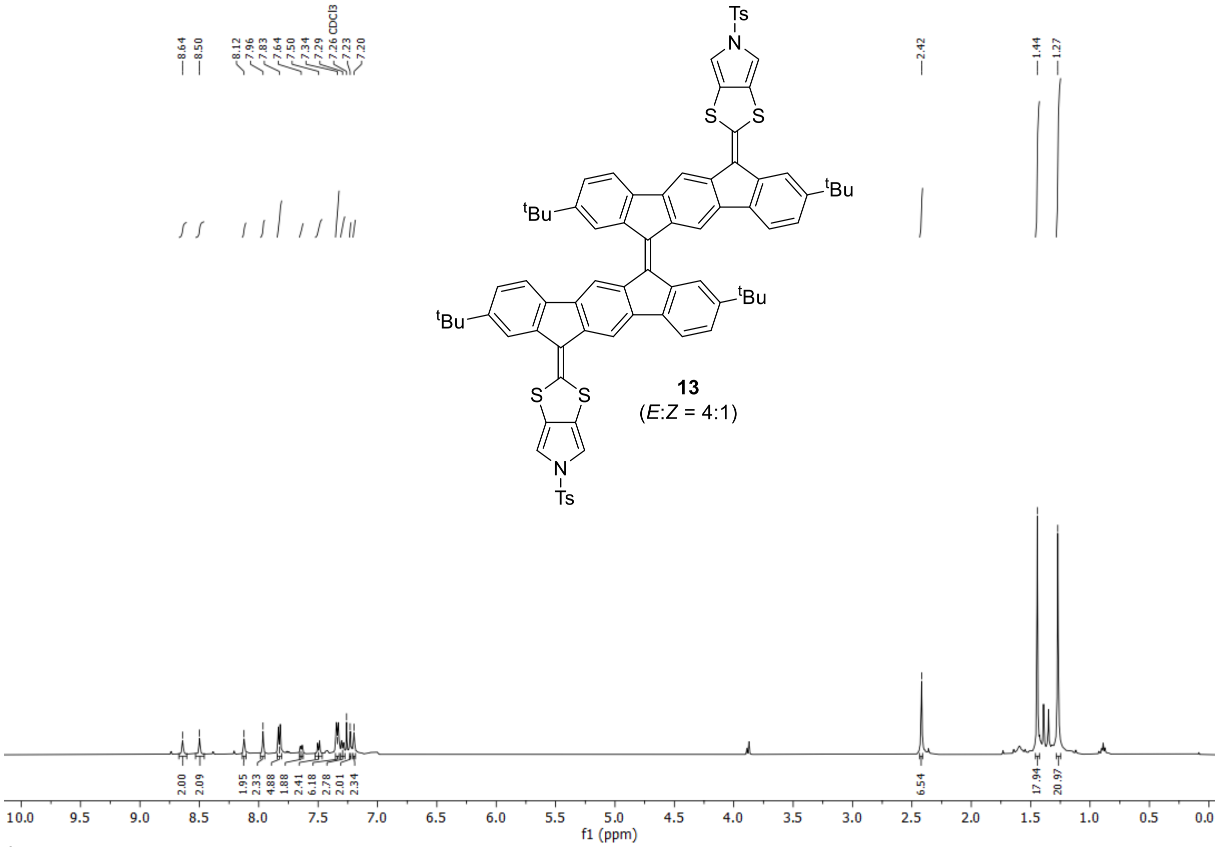

^{1}H NMR spectrum (500 MHz, $CDCl_3$) of **13**.

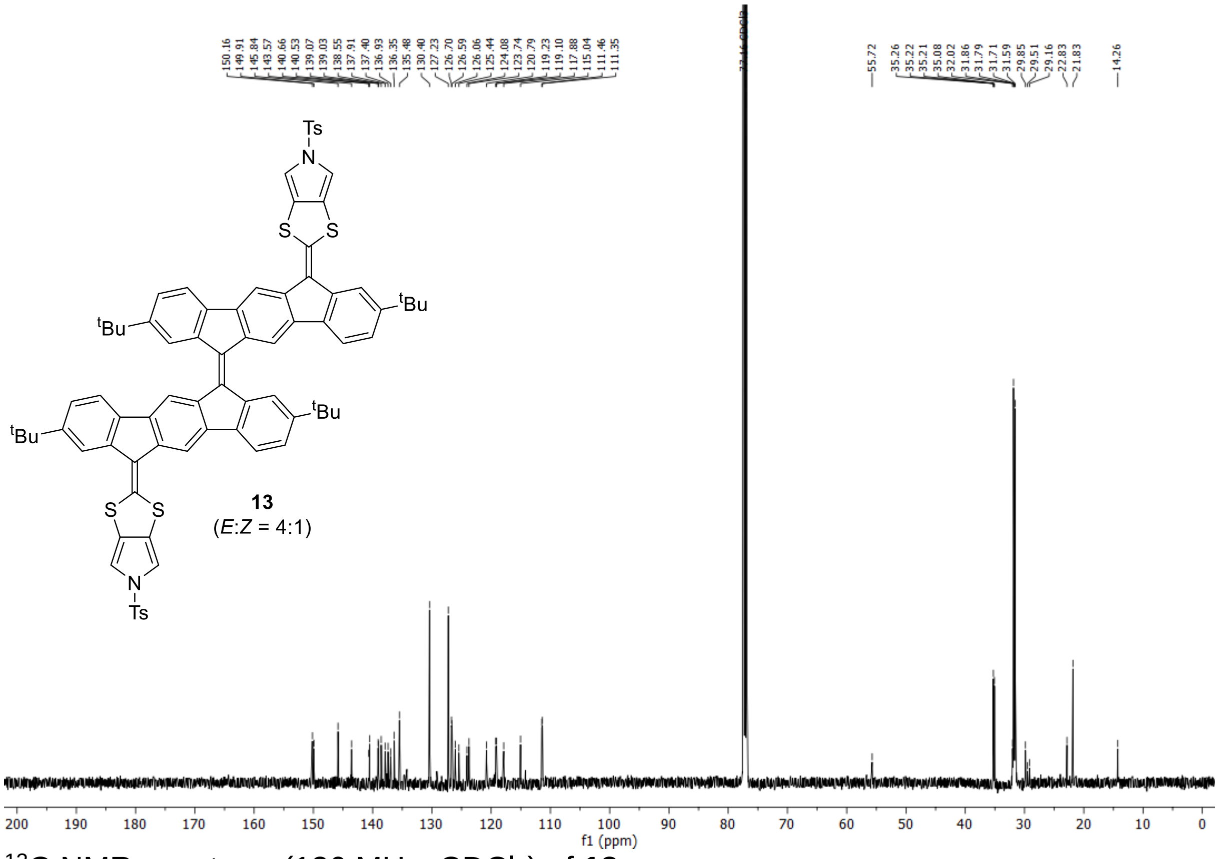

^{13}C NMR spectrum (126 MHz, $CDCl_3$) of **13**.

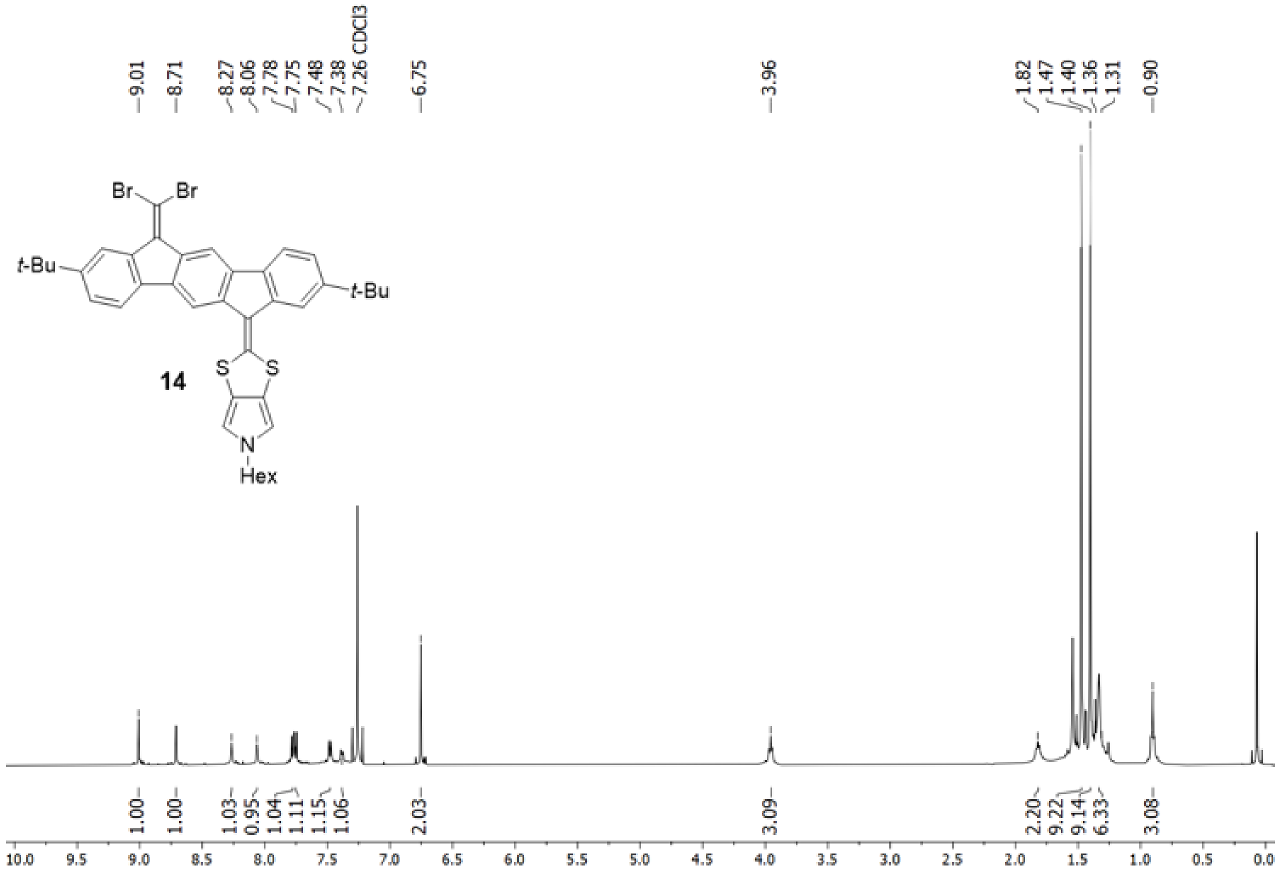

^{1}H NMR spectrum (500 MHz, $CDCl_3$) of **14**.

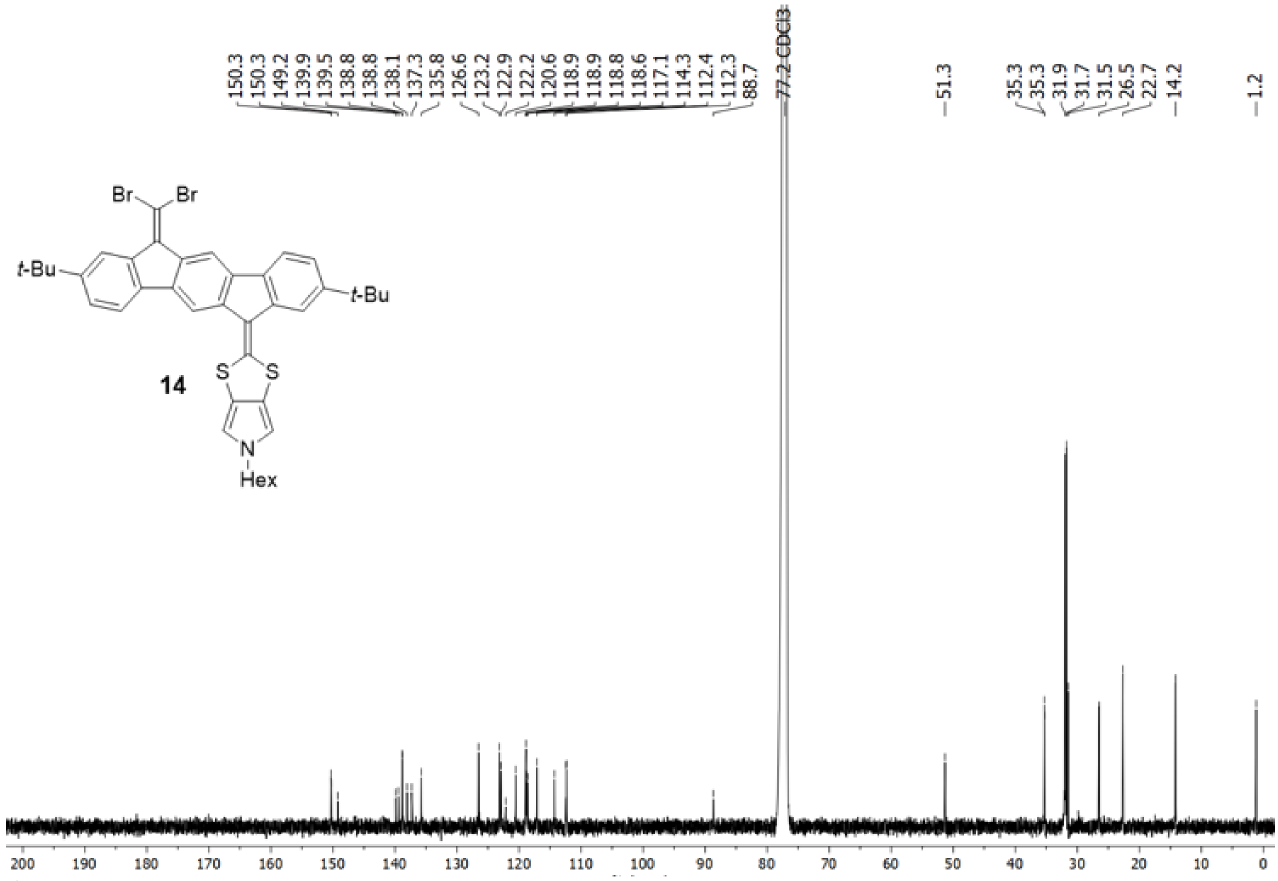

^{13}C NMR spectrum (126 MHz, $CDCl_3$) of **14**.

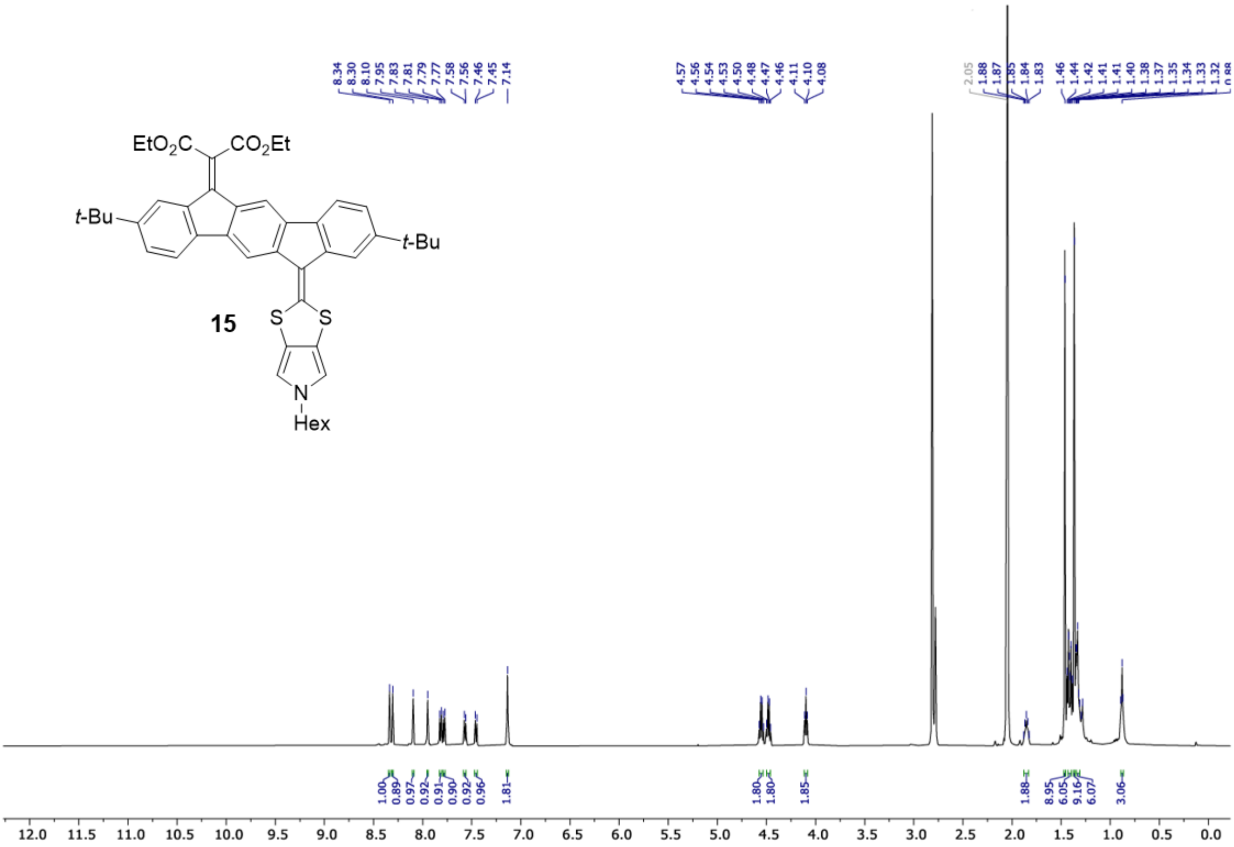

^{1}H NMR spectrum (500 MHz, $(CD_3)_2CO$) of **15**.

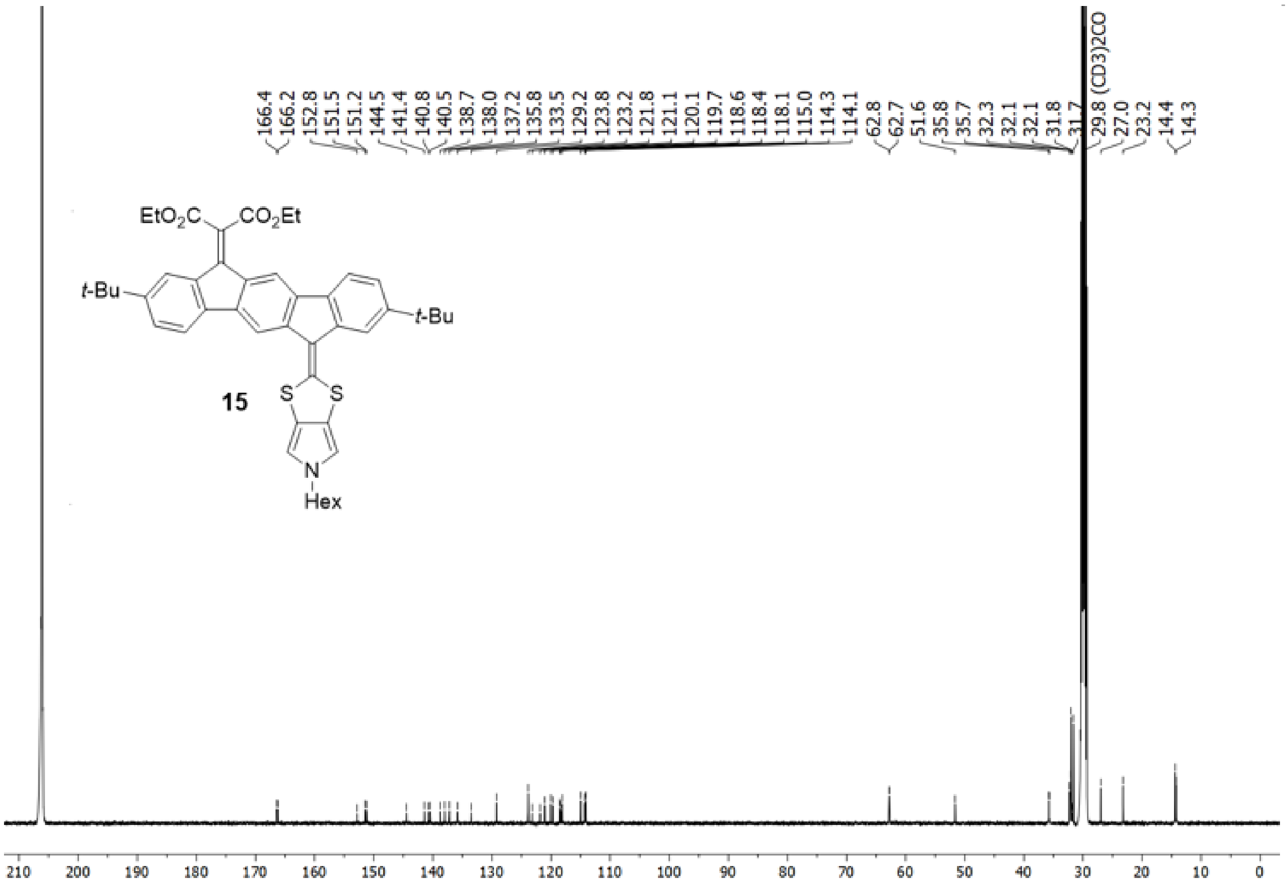

^{13}C NMR spectrum (126 MHz, $(CD_3)_2CO$) of **15**.

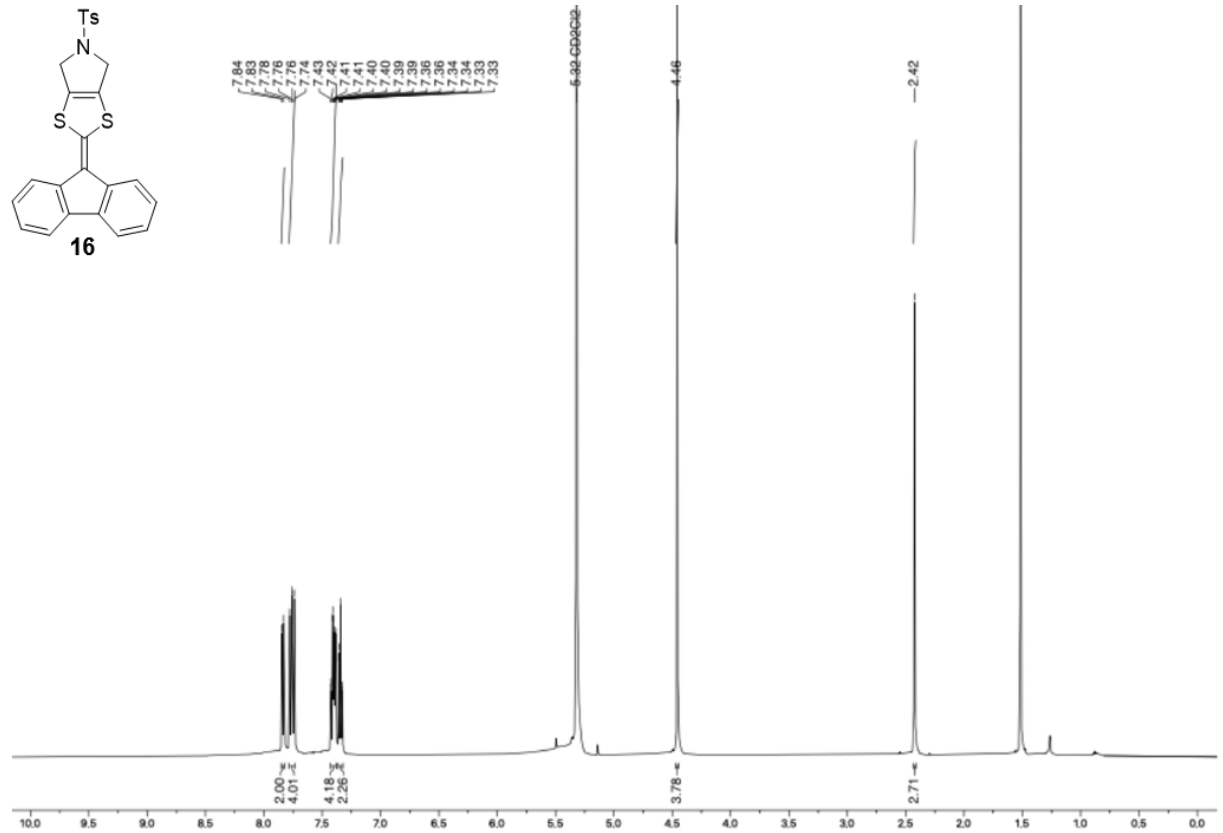

^{1}H NMR spectrum (500 MHz, CD_2Cl_2) of **16**.

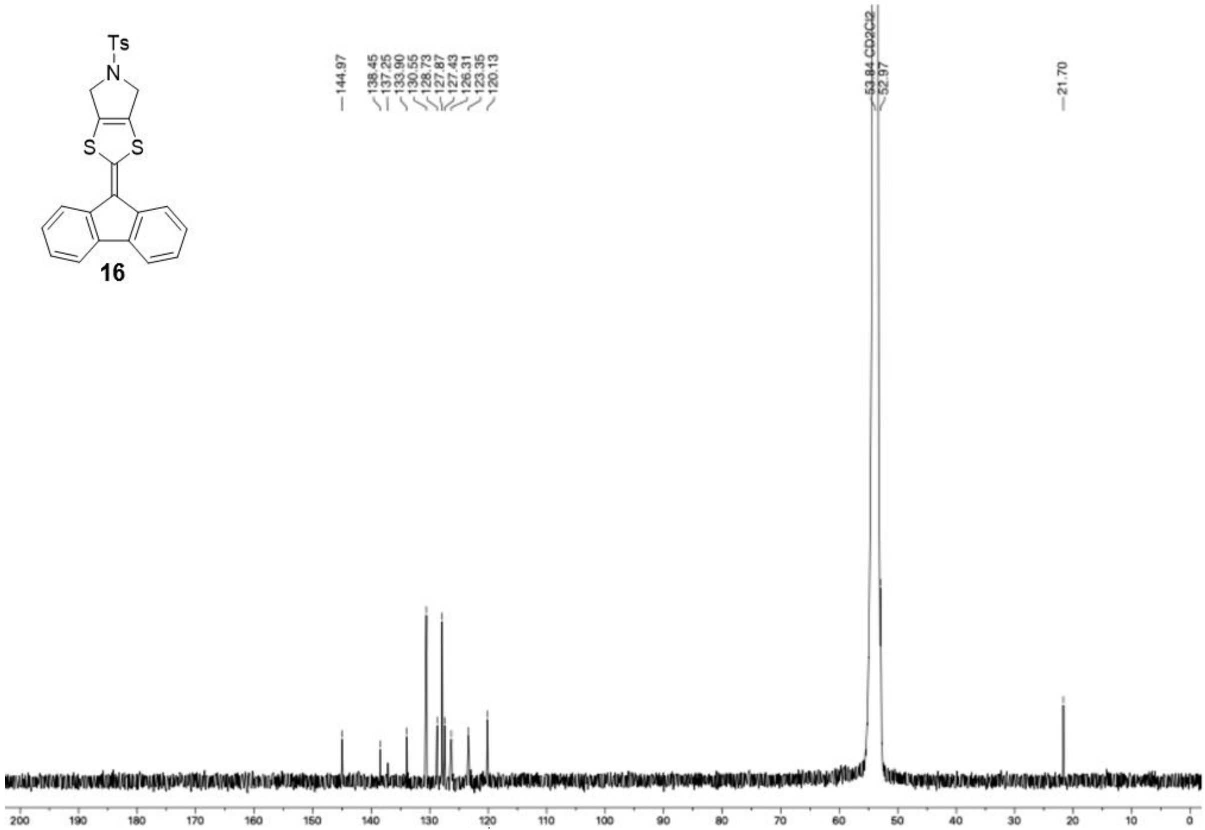

^{13}C NMR spectrum (126 MHz, CD_2Cl_2) of **16**.

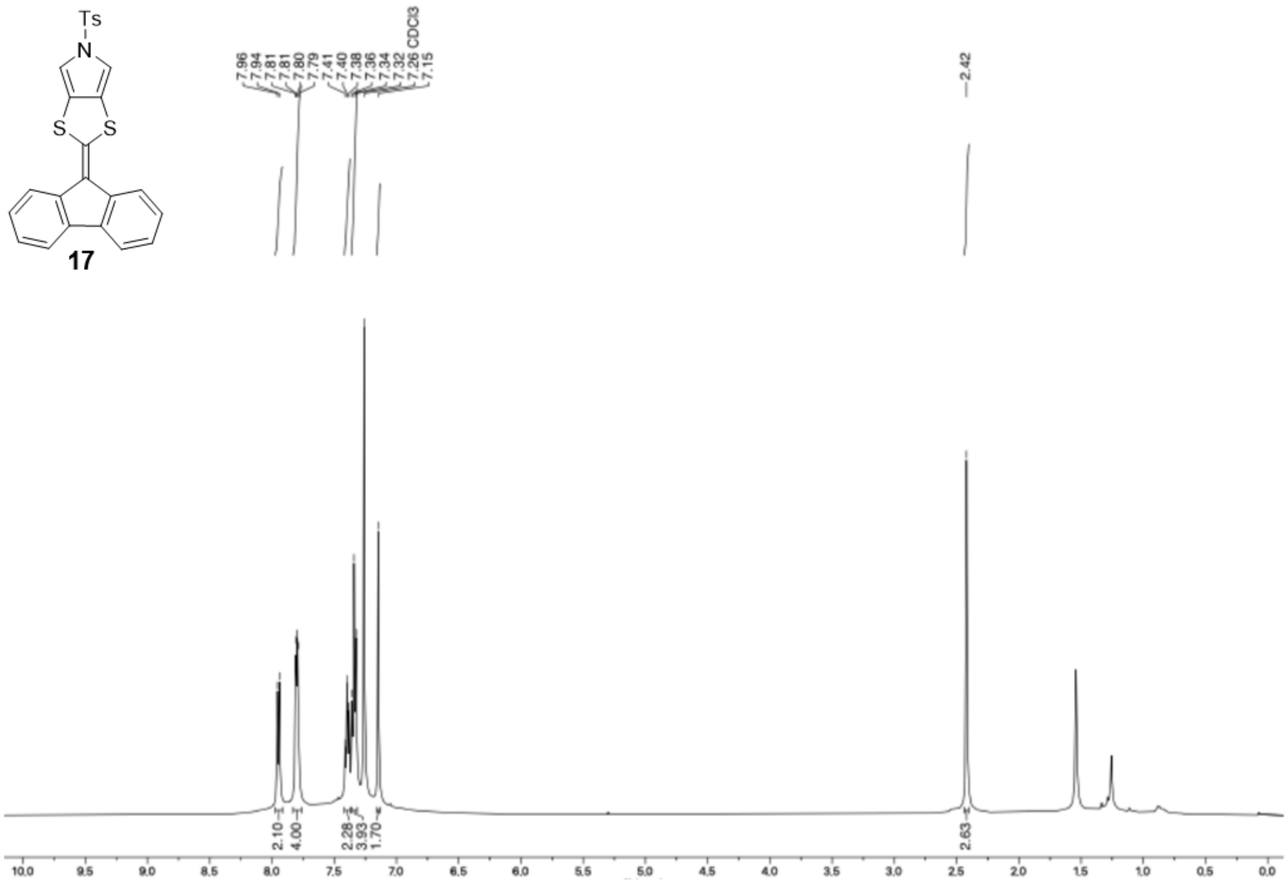

17

^1H NMR spectrum (500 MHz, $CDCl_3$) of **17**.

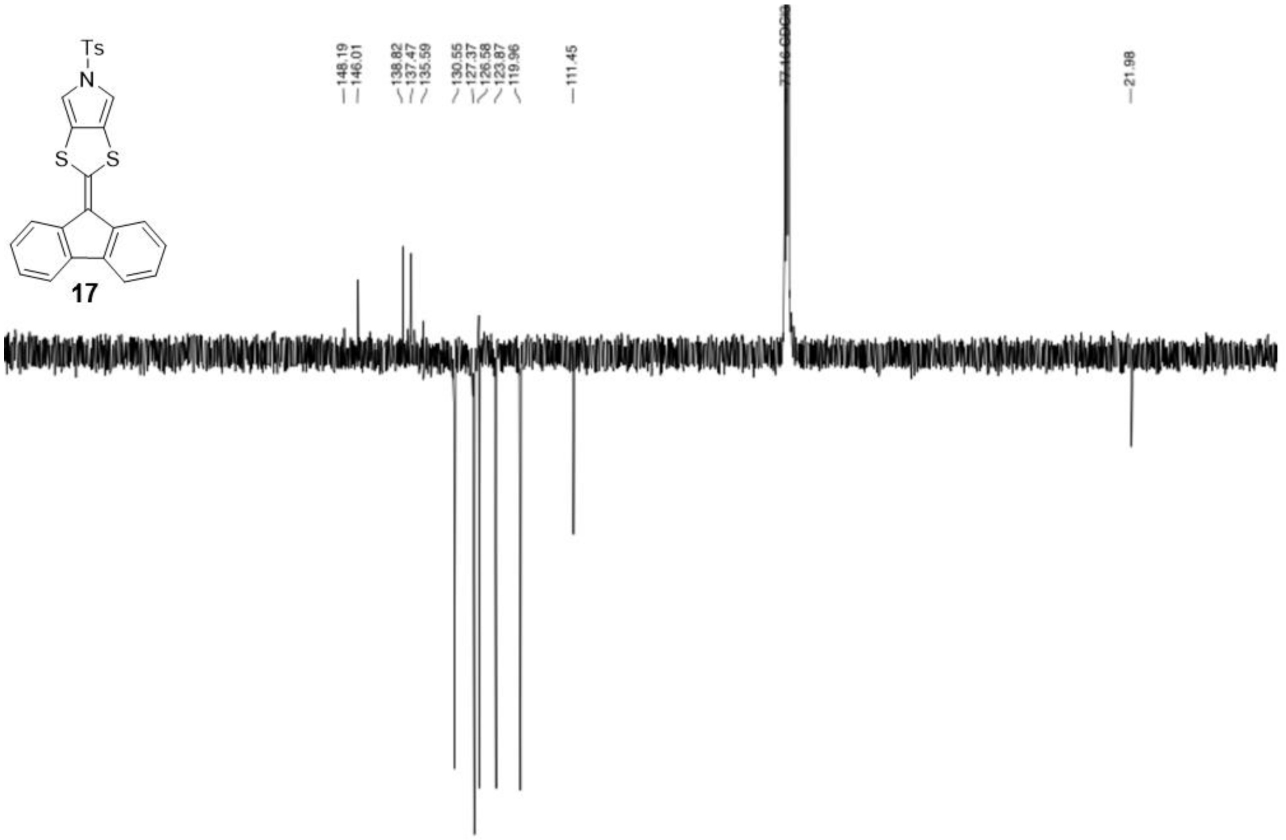

17

^{13}C NMR spectrum (126 MHz, $CDCl_3$) of **17**.

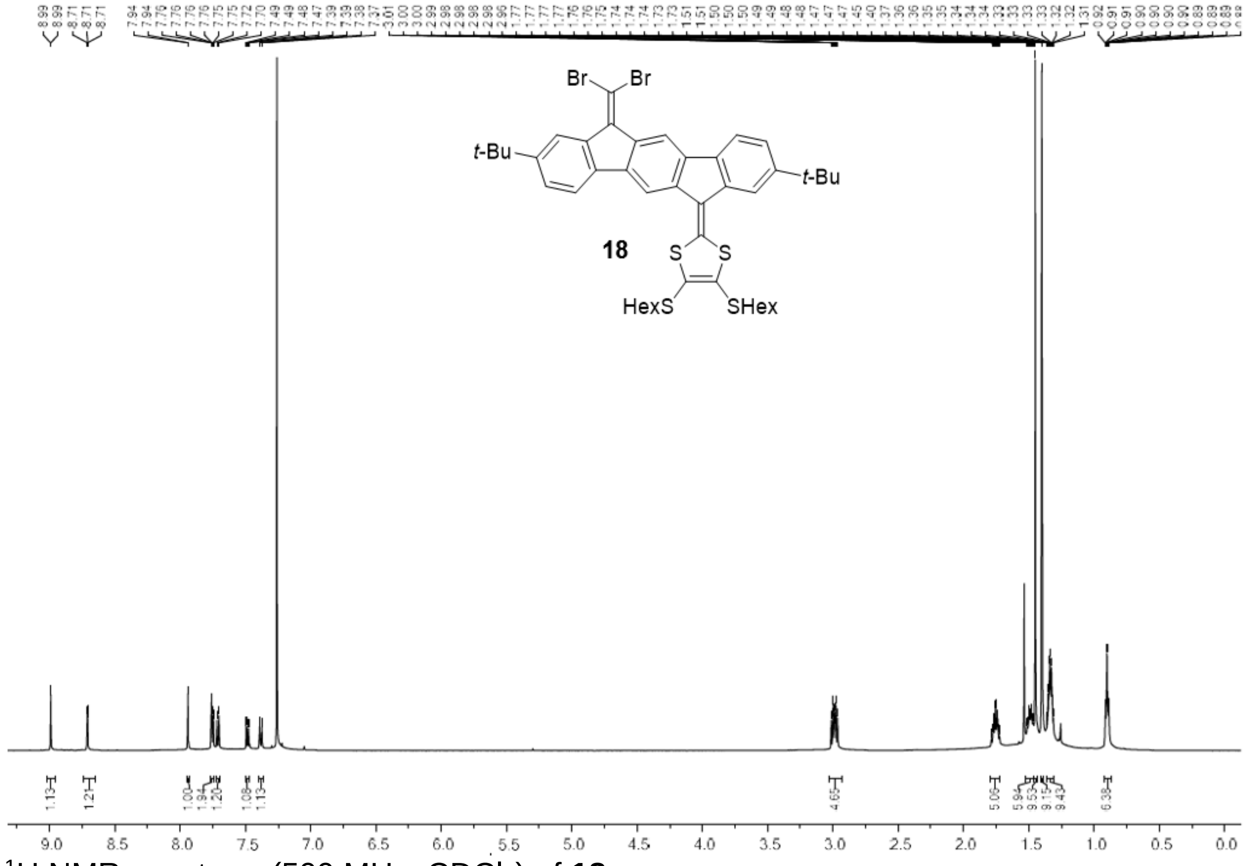

^{1}H NMR spectrum (500 MHz, $CDCl_3$) of **18**.

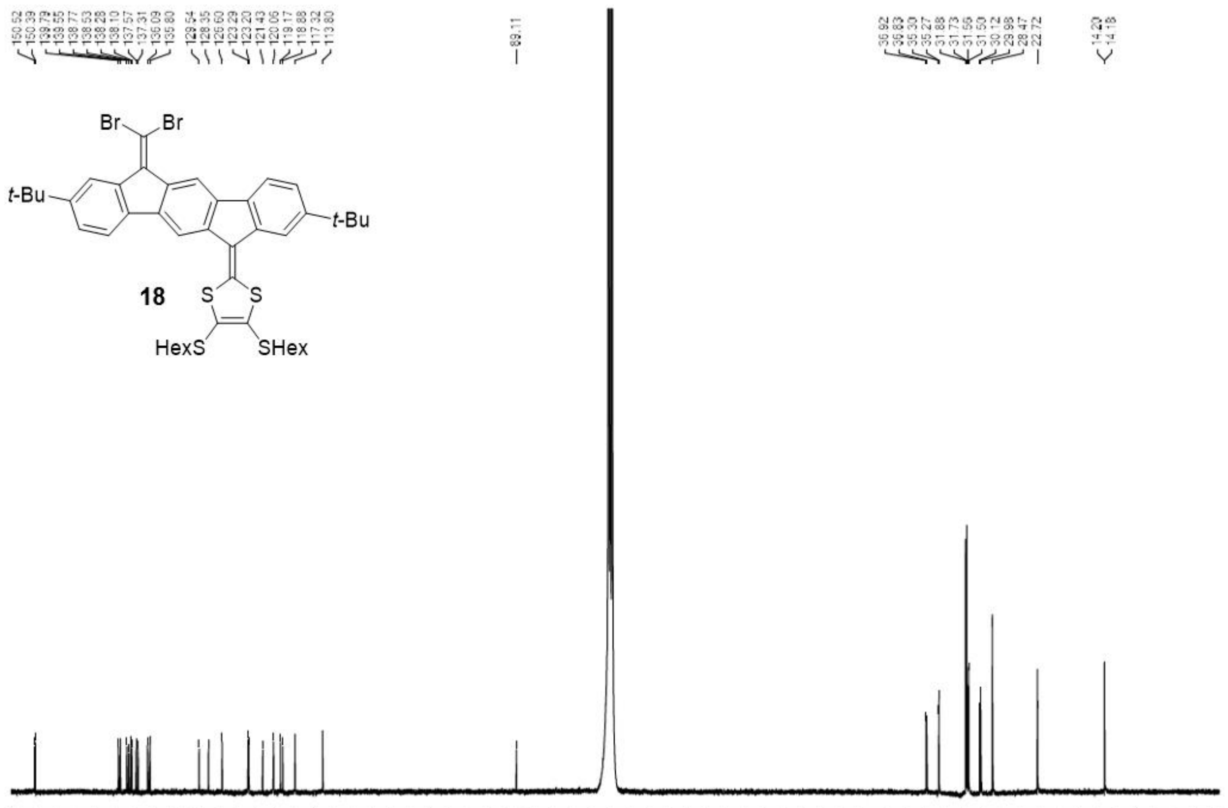

^{13}C NMR spectrum (126 MHz, $CDCl_3$) of **18**.

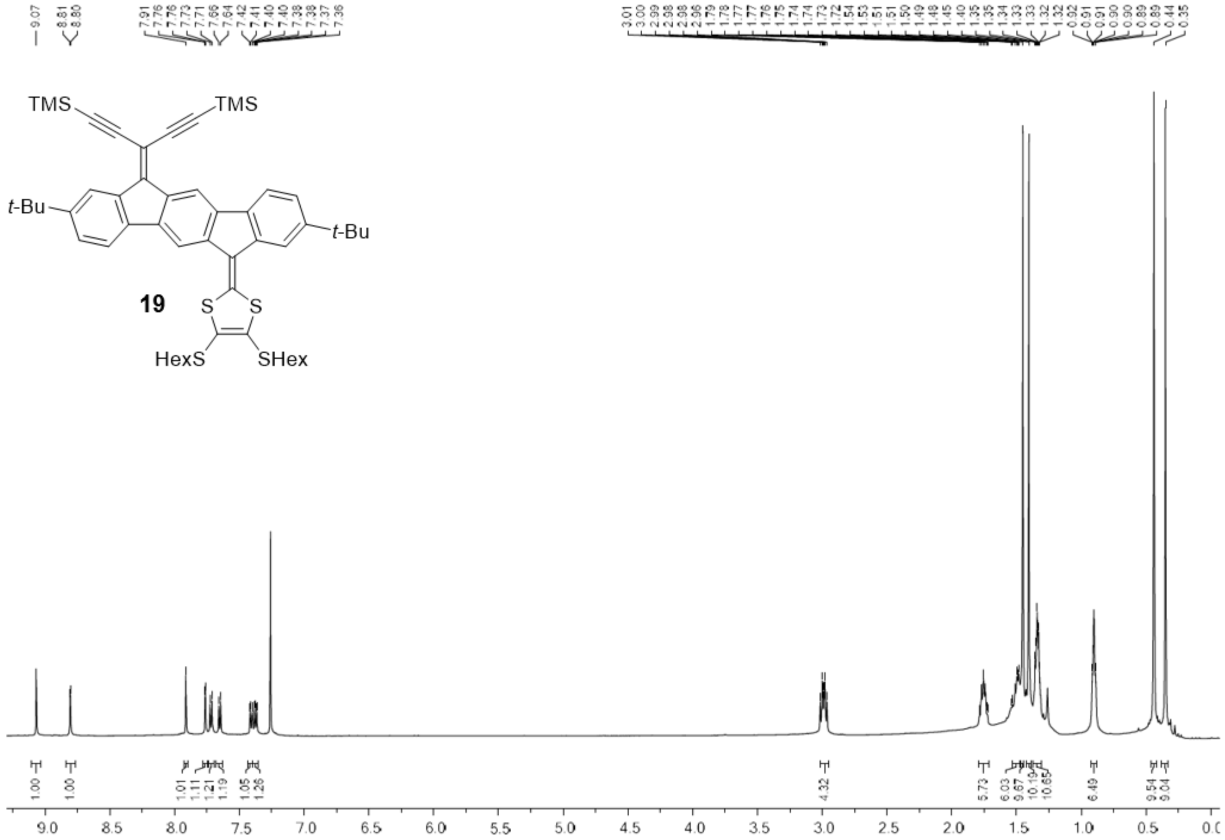

^{1}H NMR spectrum (500 MHz, $CDCl_3$) of **19**.

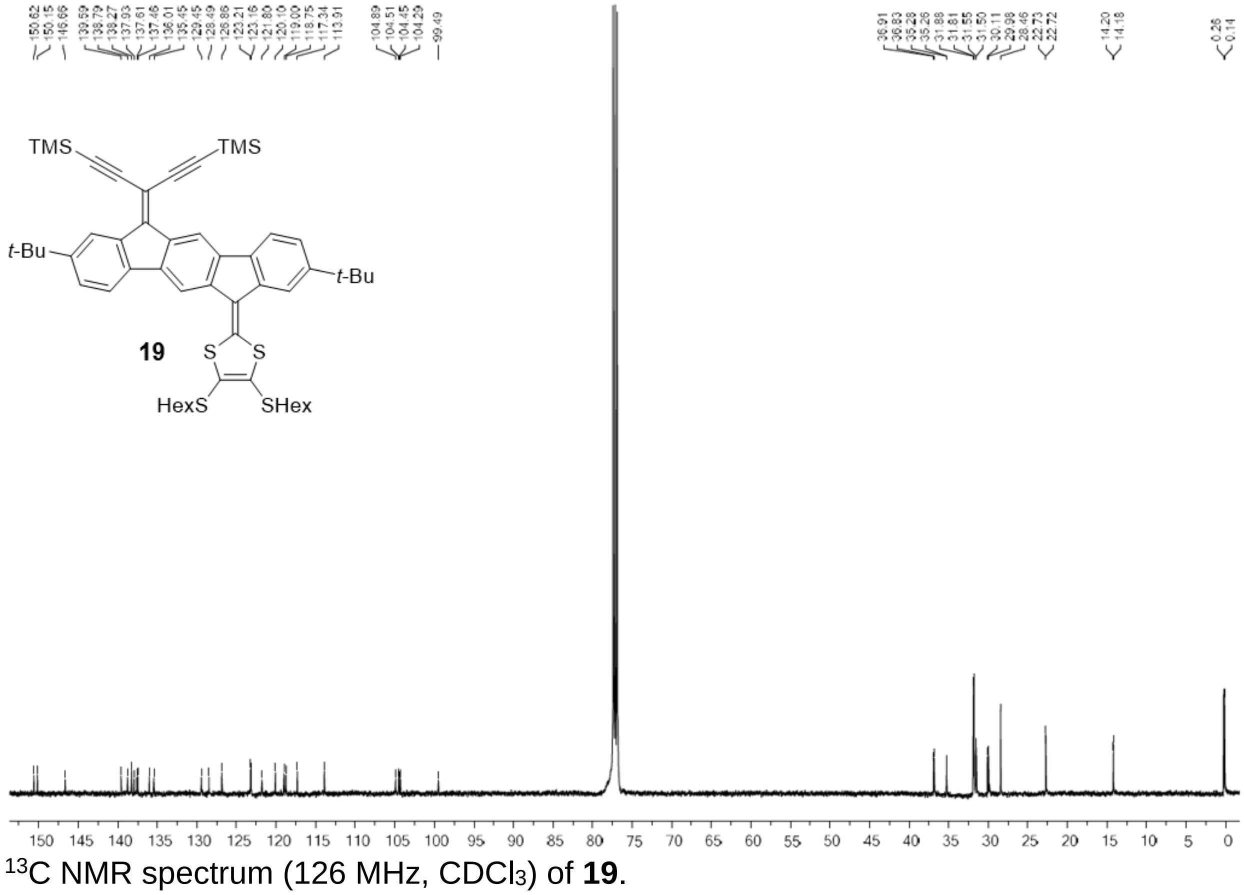

^{13}C NMR spectrum (126 MHz, $CDCl_3$) of **19**.

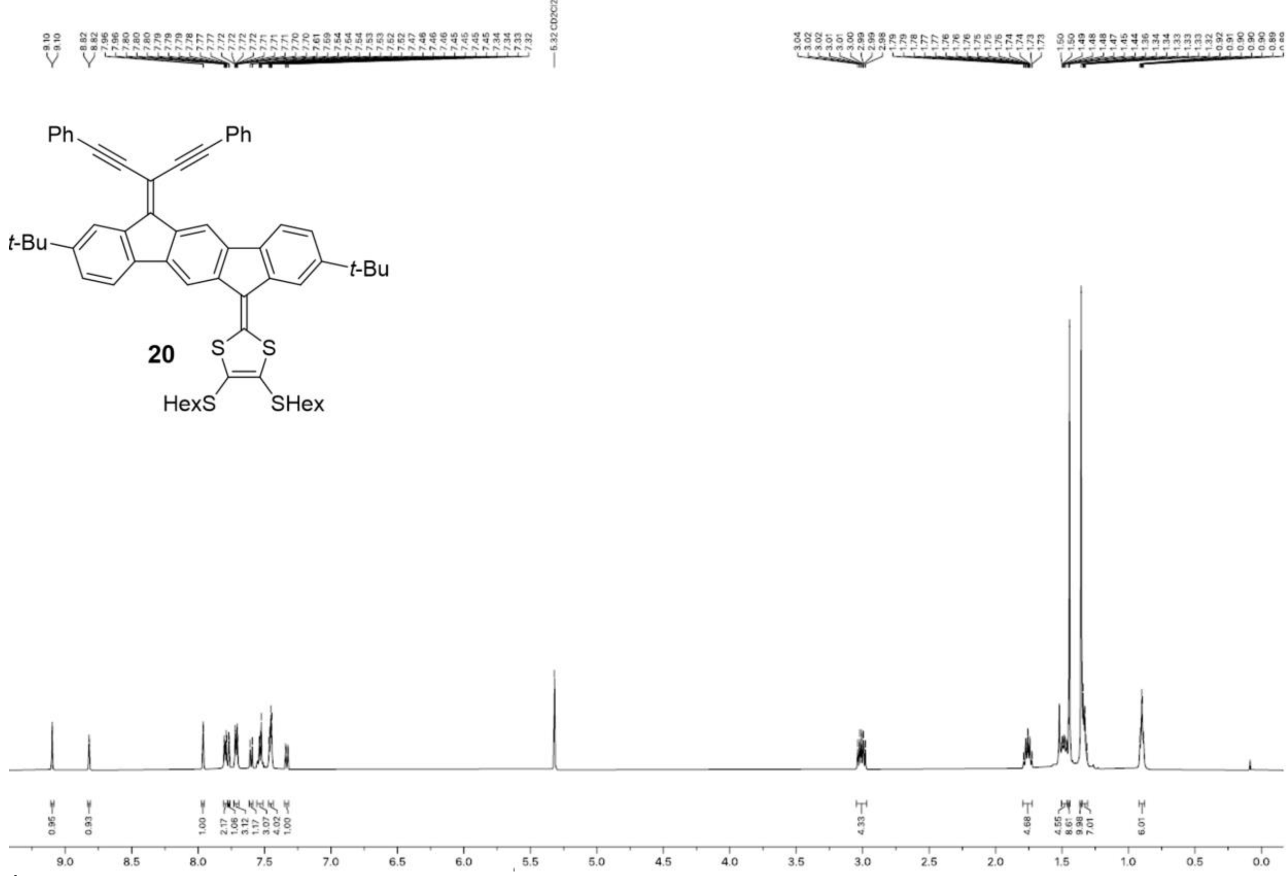

^{1}H NMR spectrum (500 MHz, CD_2Cl_2) of **20**.

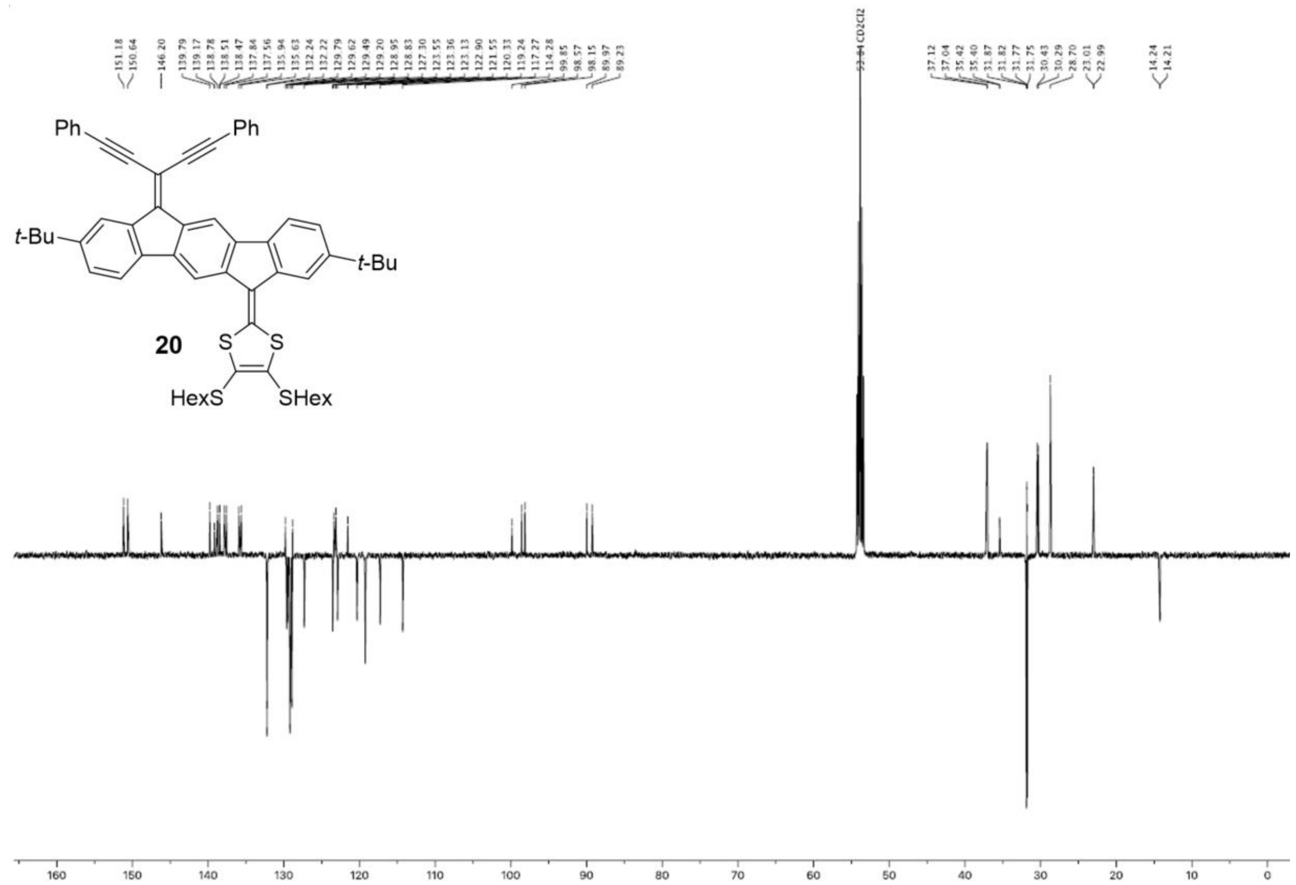

^{13}C NMR spectrum (126 MHz, CD_2Cl_2) of **20**.

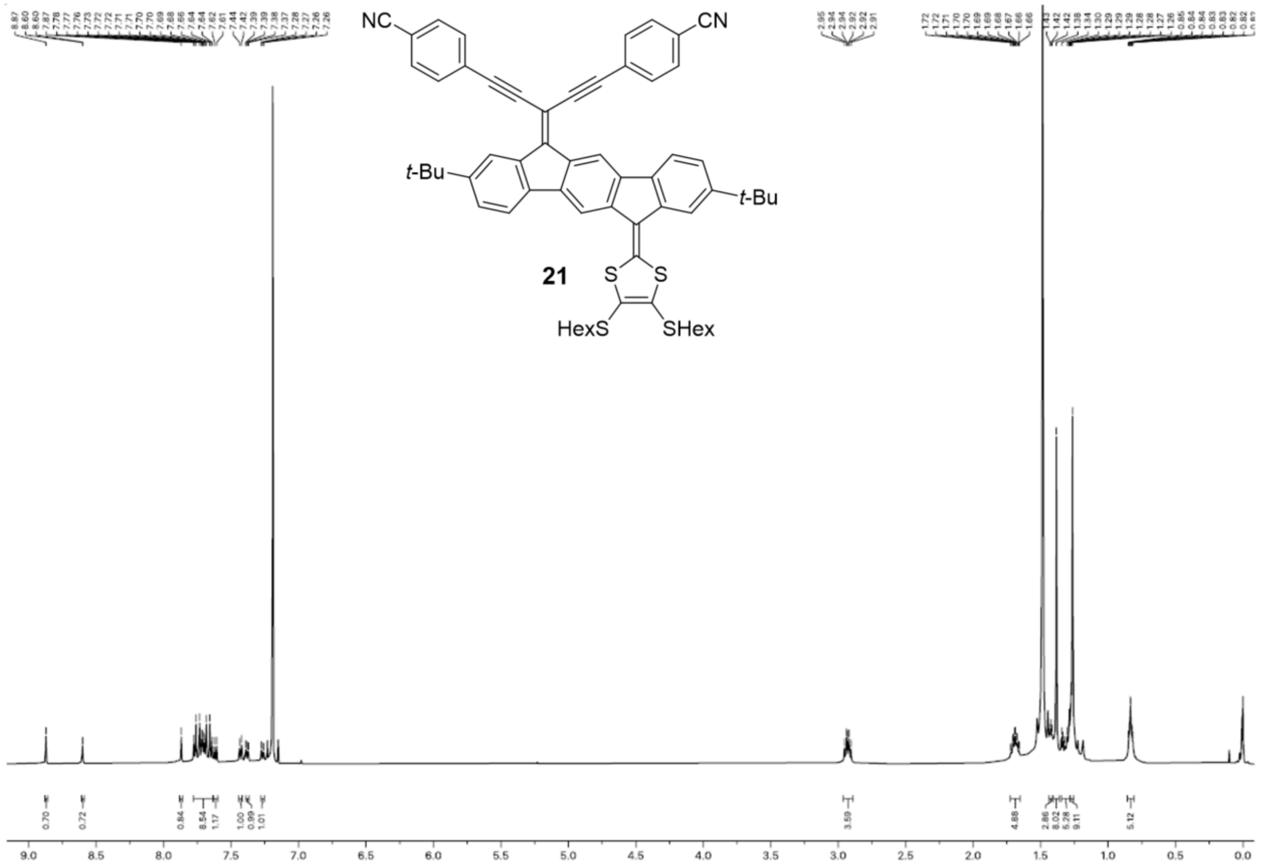

^{1}H NMR spectrum (500 MHz, $CDCl_3$) of **21**.

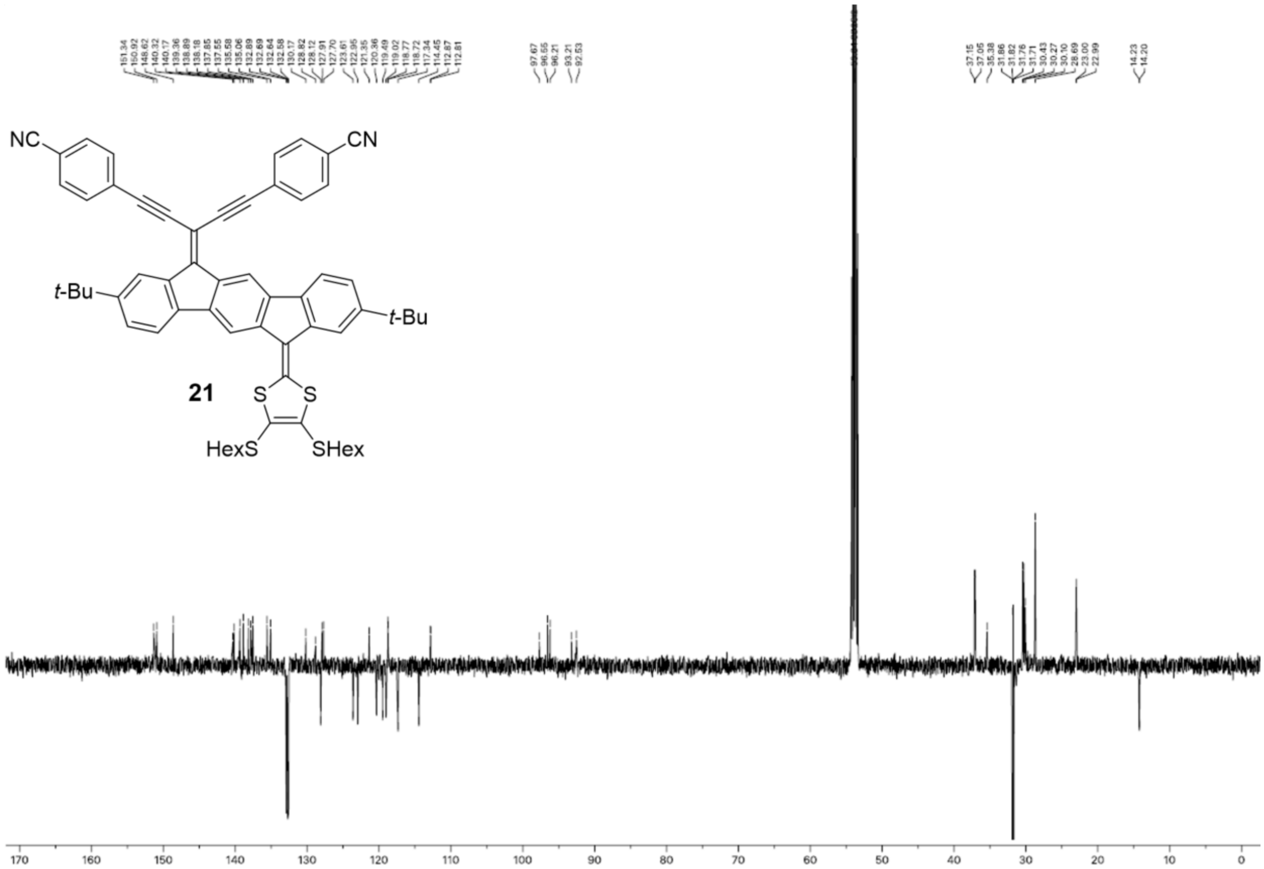

^{13}C NMR spectrum (126 MHz, $CDCl_3$) of **21**.

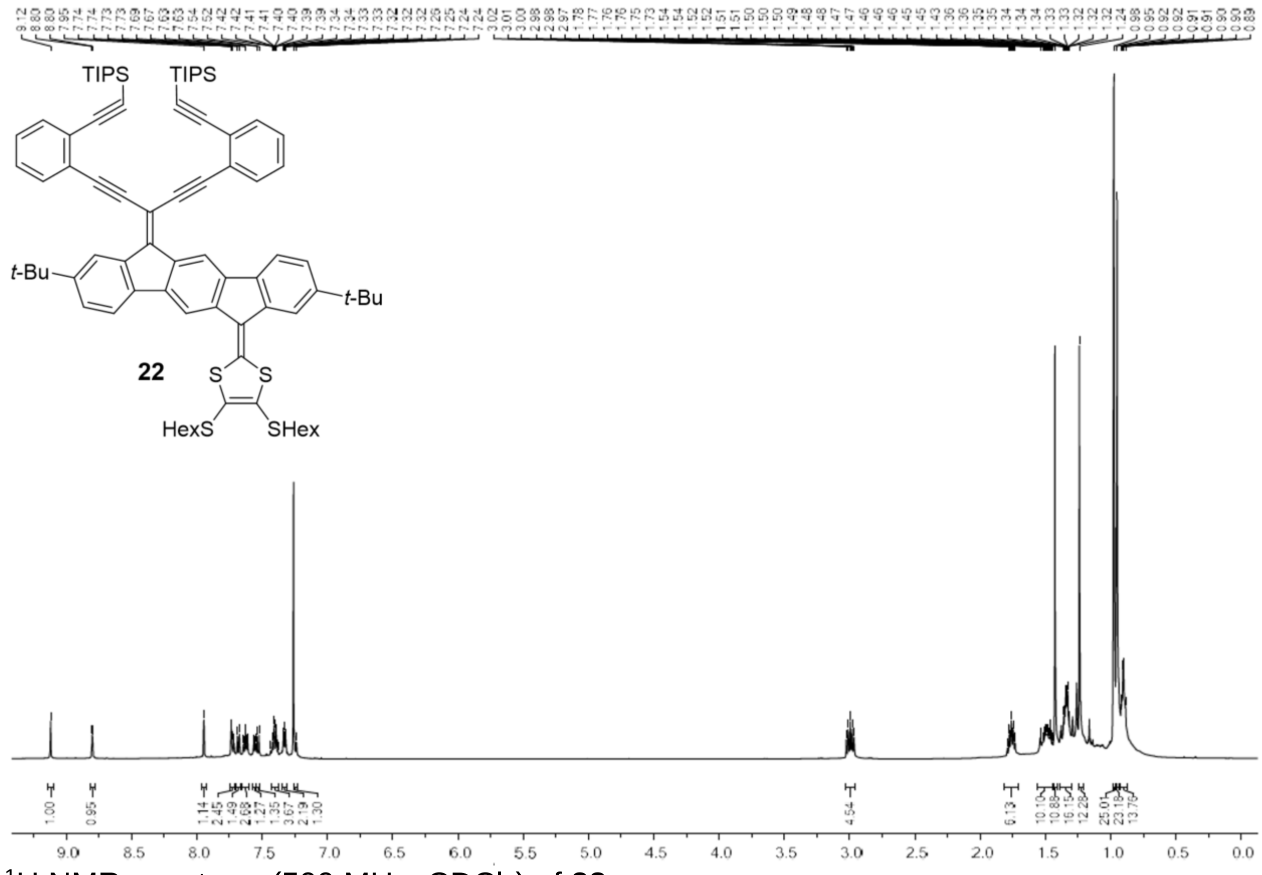

^{1}H NMR spectrum (500 MHz, $CDCl_3$) of **22**.

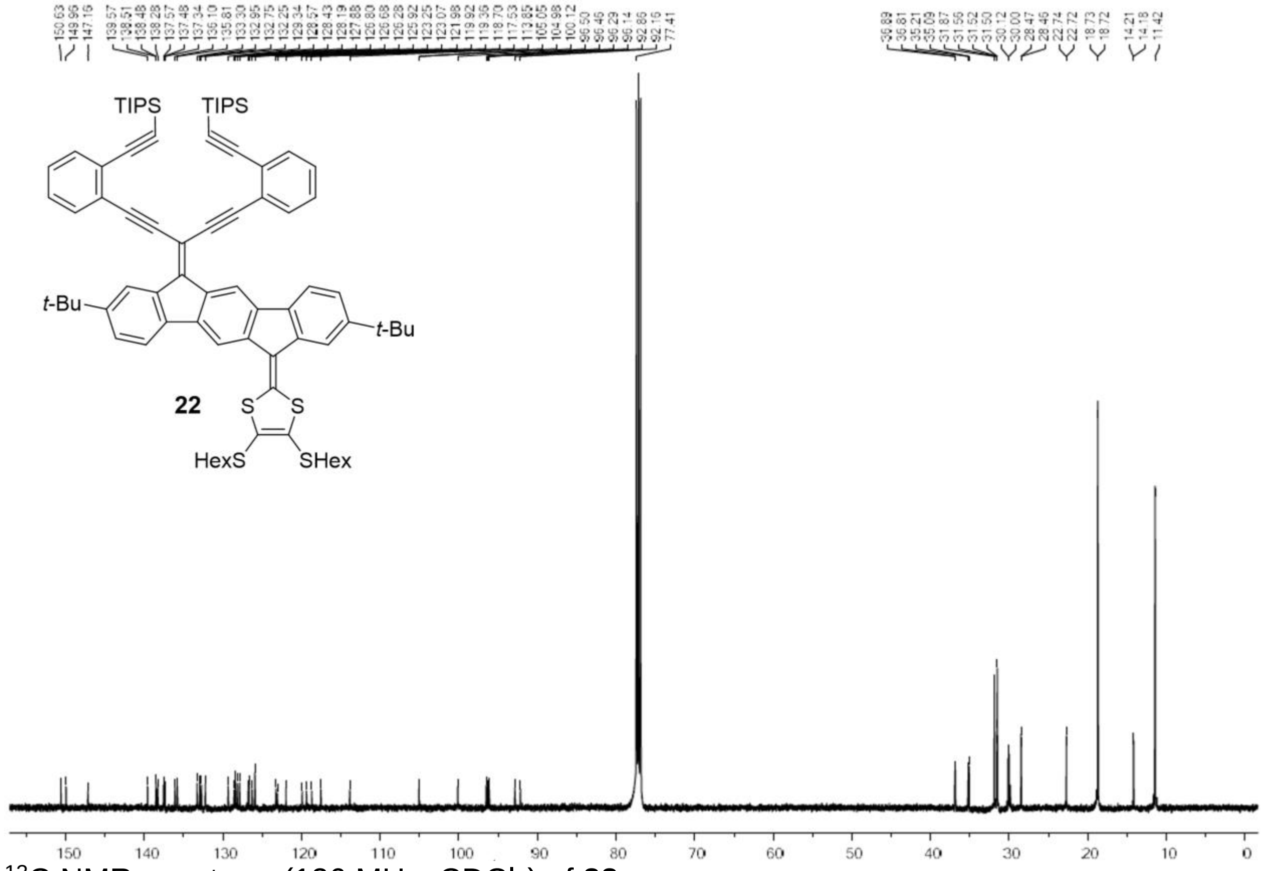

^{13}C NMR spectrum (126 MHz, $CDCl_3$) of **22**.

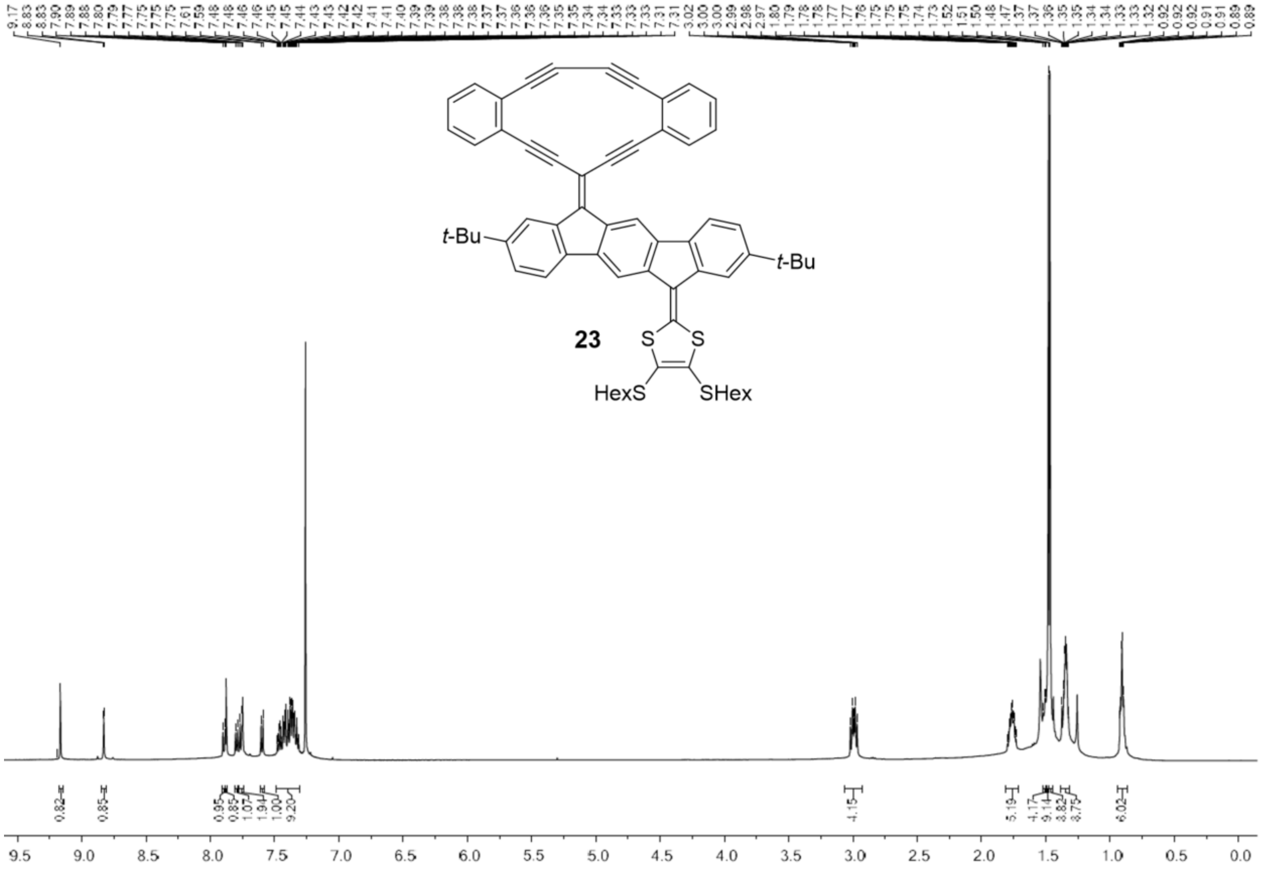

^{1}H NMR spectrum (500 MHz, $CDCl_3$) of **23**.

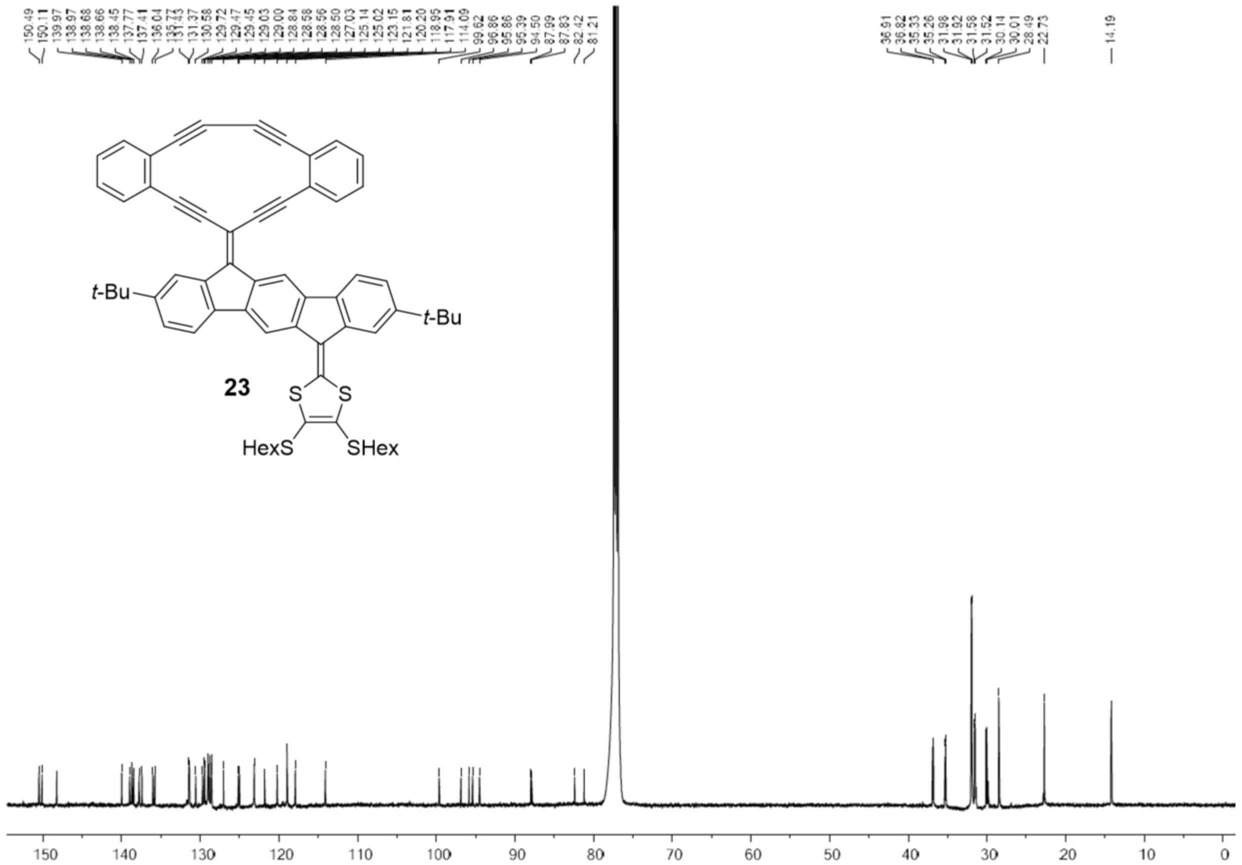

^{13}C NMR spectrum (126 MHz, $CDCl_3$) of **23**.

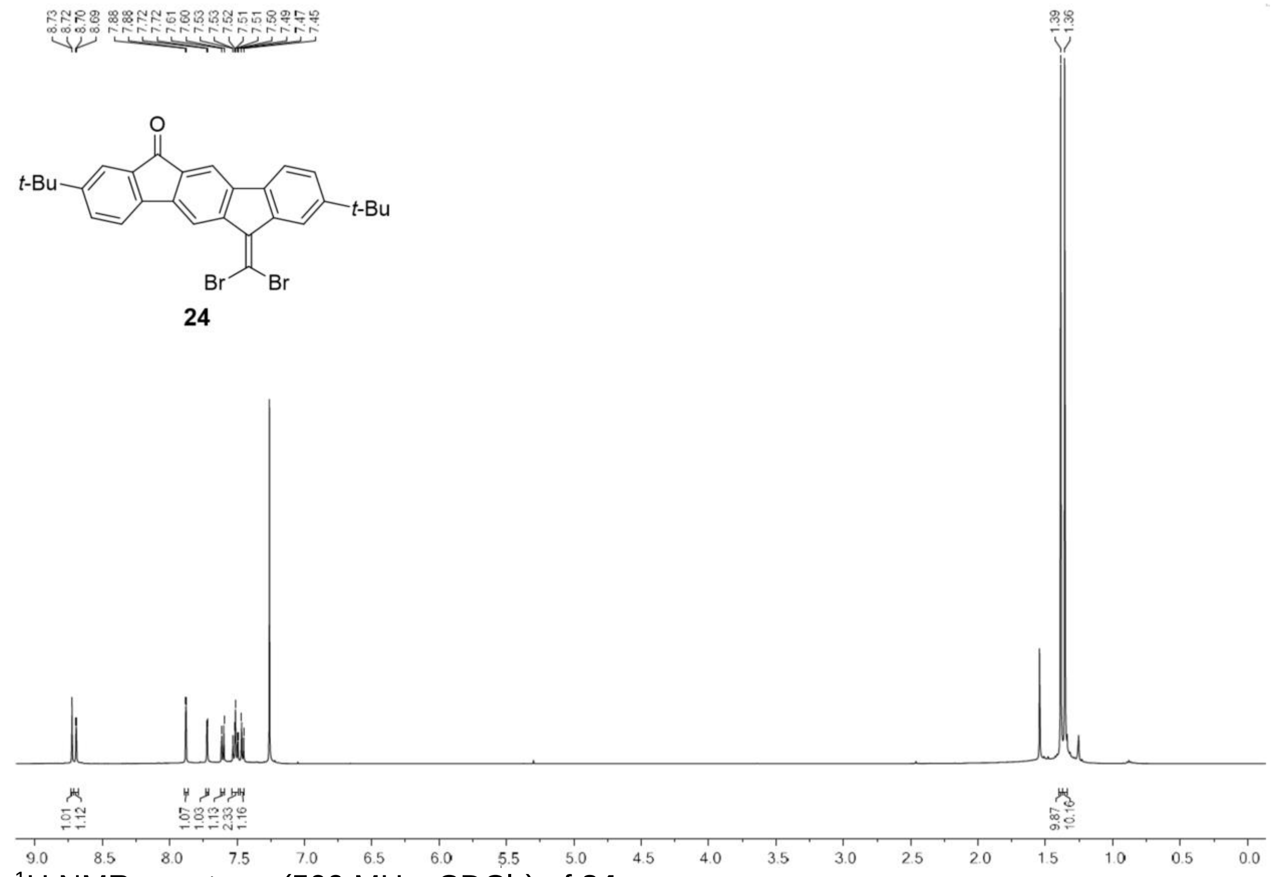

^{1}H NMR spectrum (500 MHz, $CDCl_3$) of **24**.

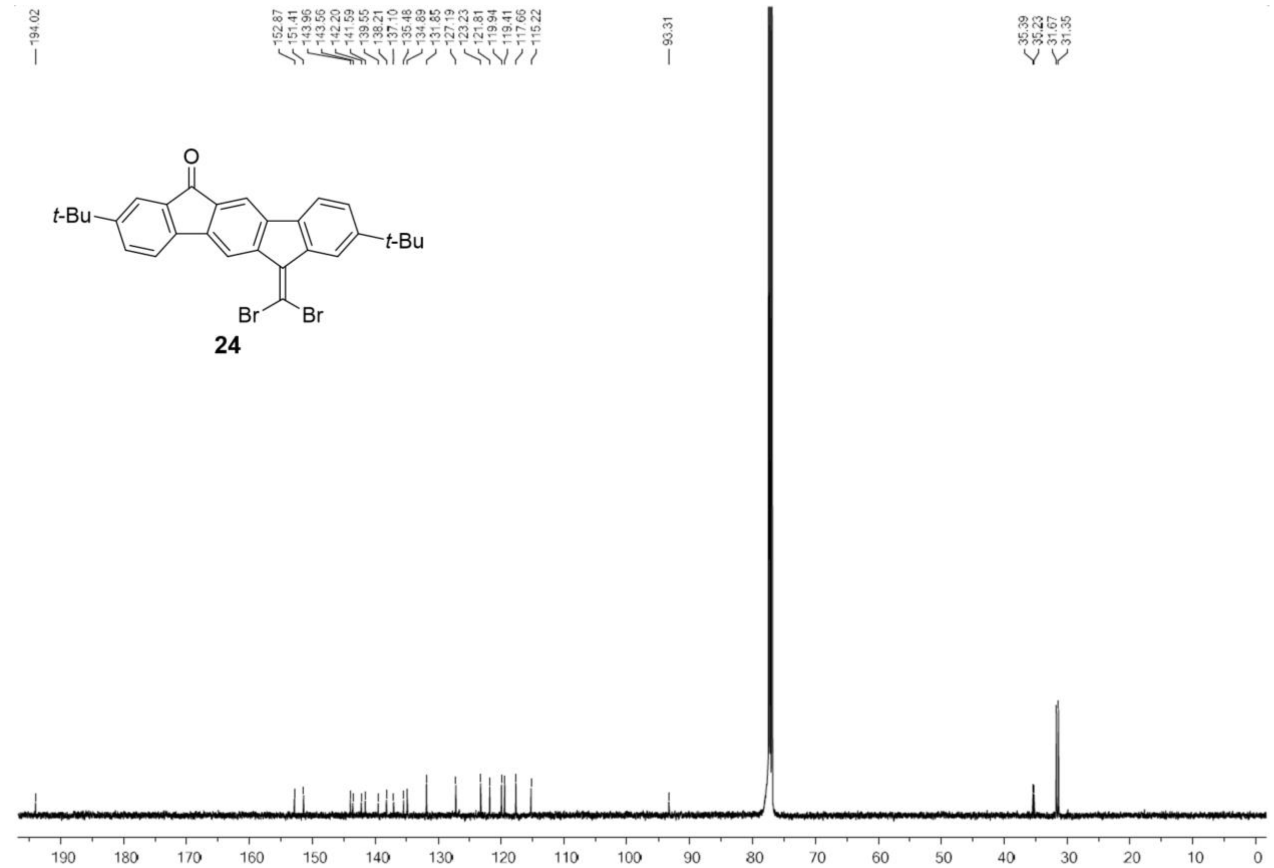

^{13}C NMR spectrum (126 MHz, $CDCl_3$) of **24**.

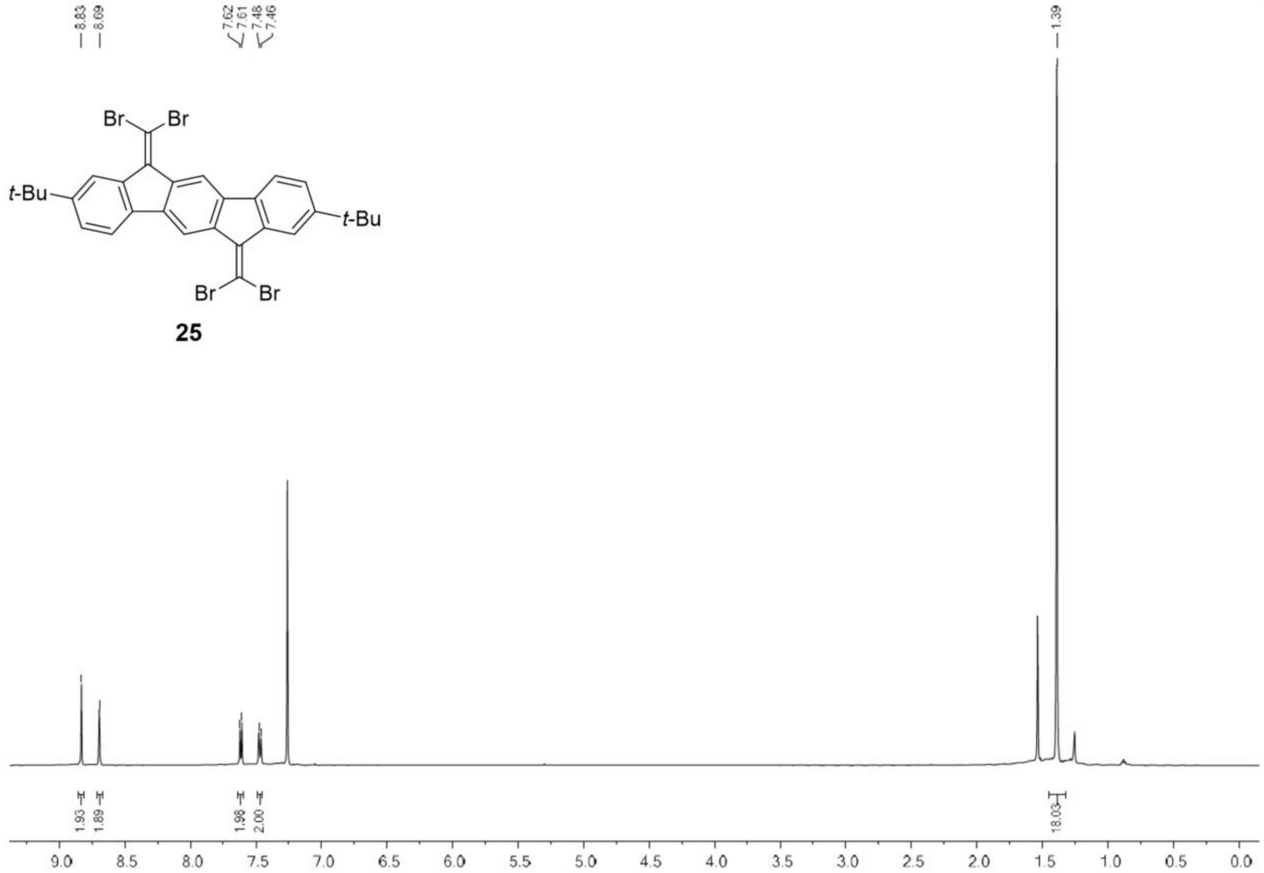

^{1}H NMR spectrum (500 MHz, $CDCl_3$) of **25**.

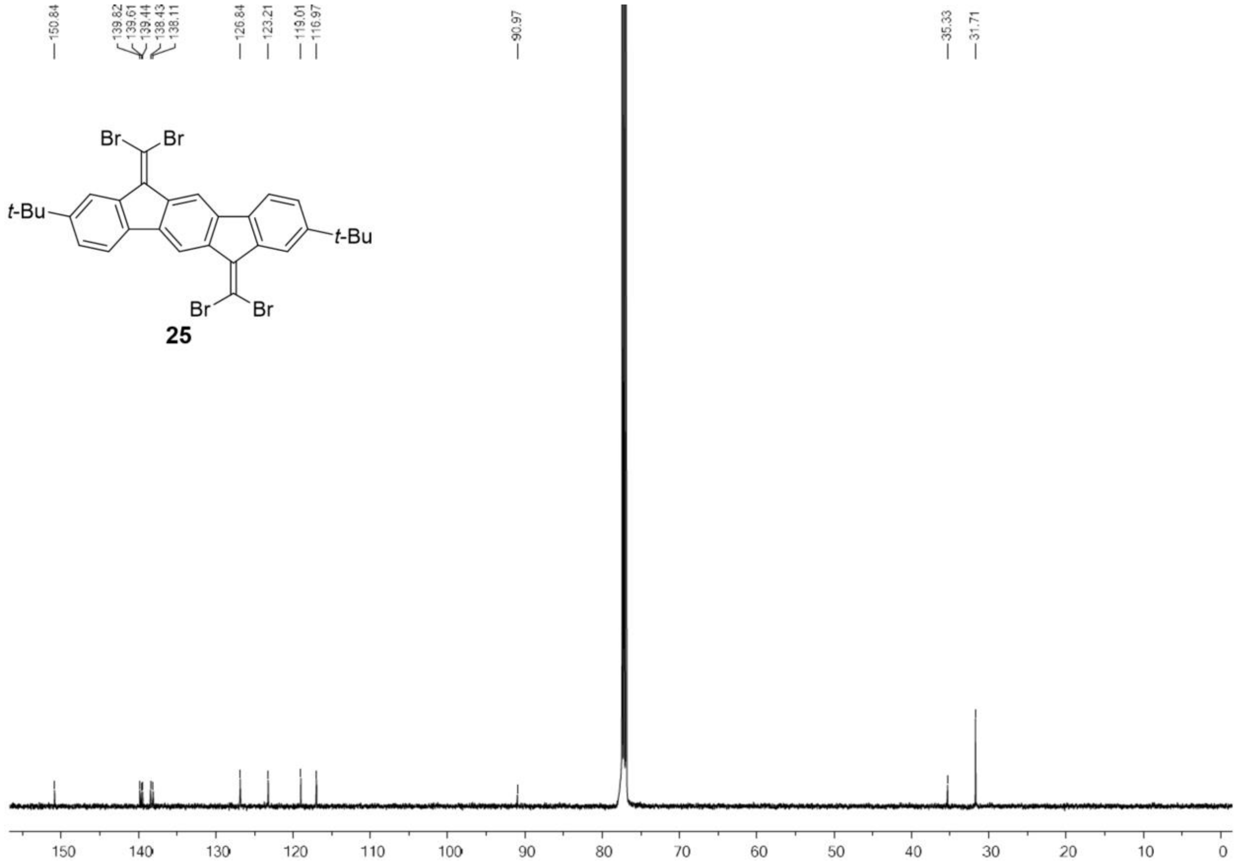

^{13}C NMR spectrum (126 MHz, $CDCl_3$) of **25**.

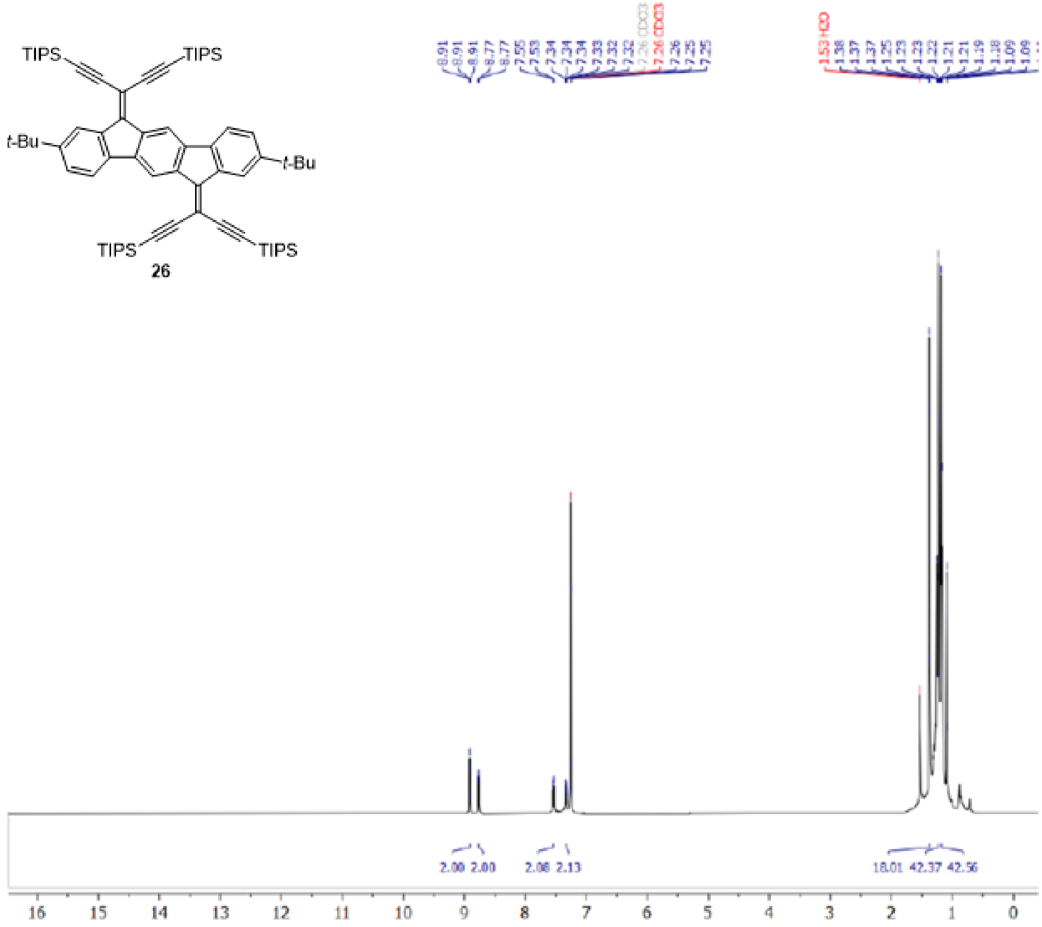

^{1}H NMR spectrum (500 MHz, $CDCl_3$) of **26**.

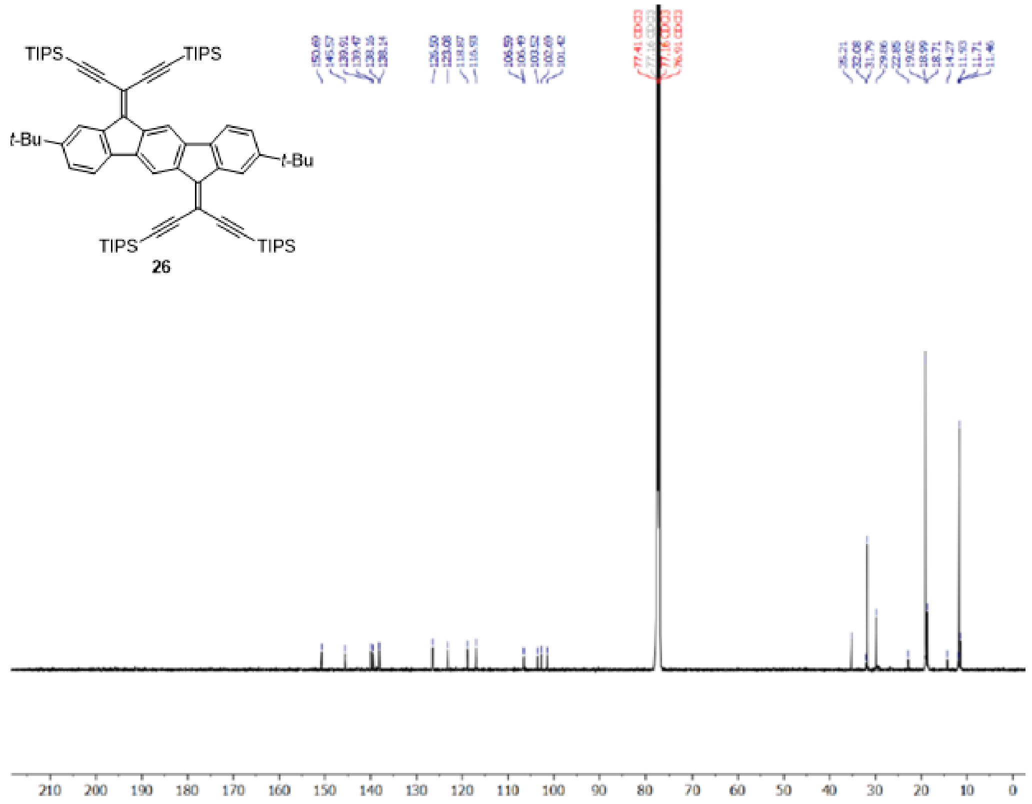

^{13}C NMR spectrum (126 MHz, $CDCl_3$) of **26**.

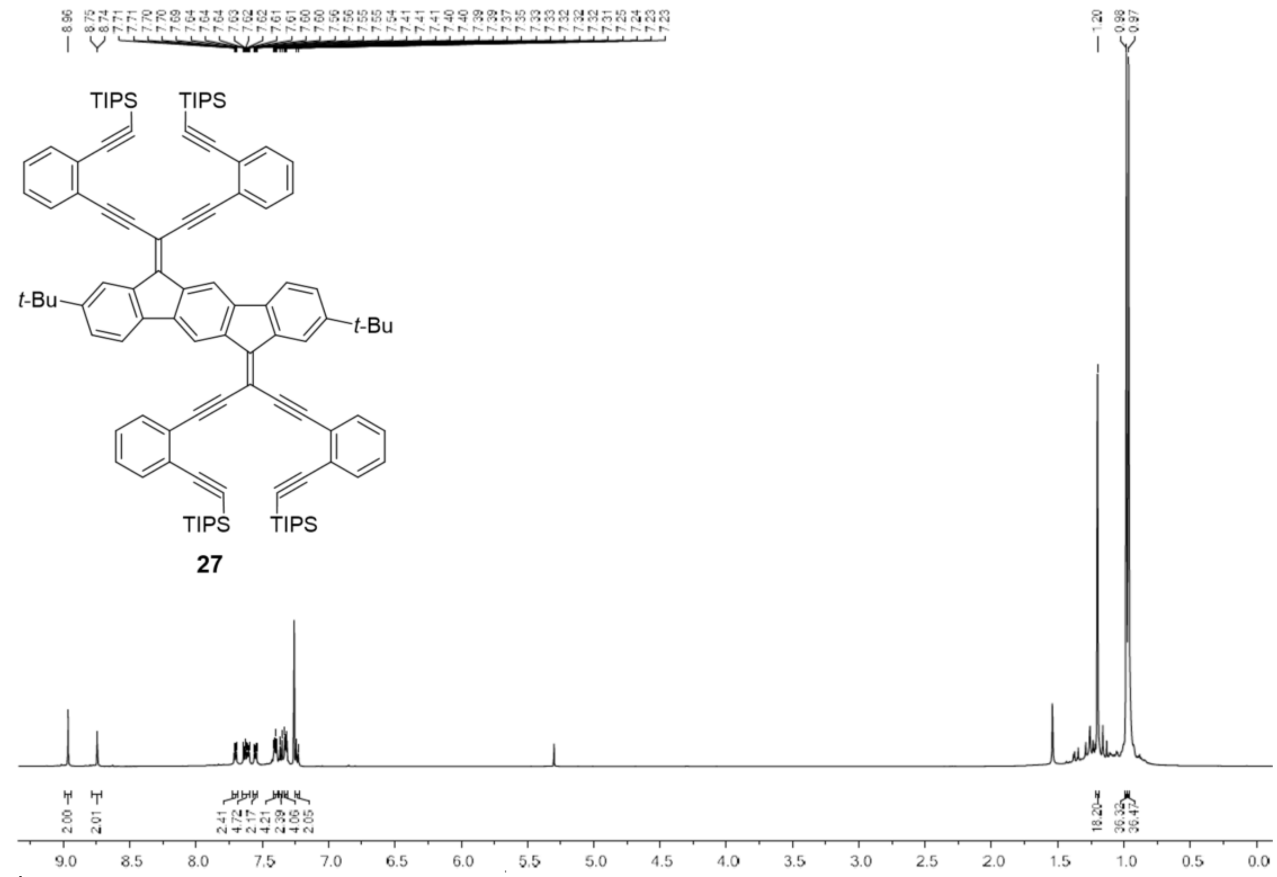

^{1}H NMR spectrum (500 MHz, $CDCl_3$) of **27**.

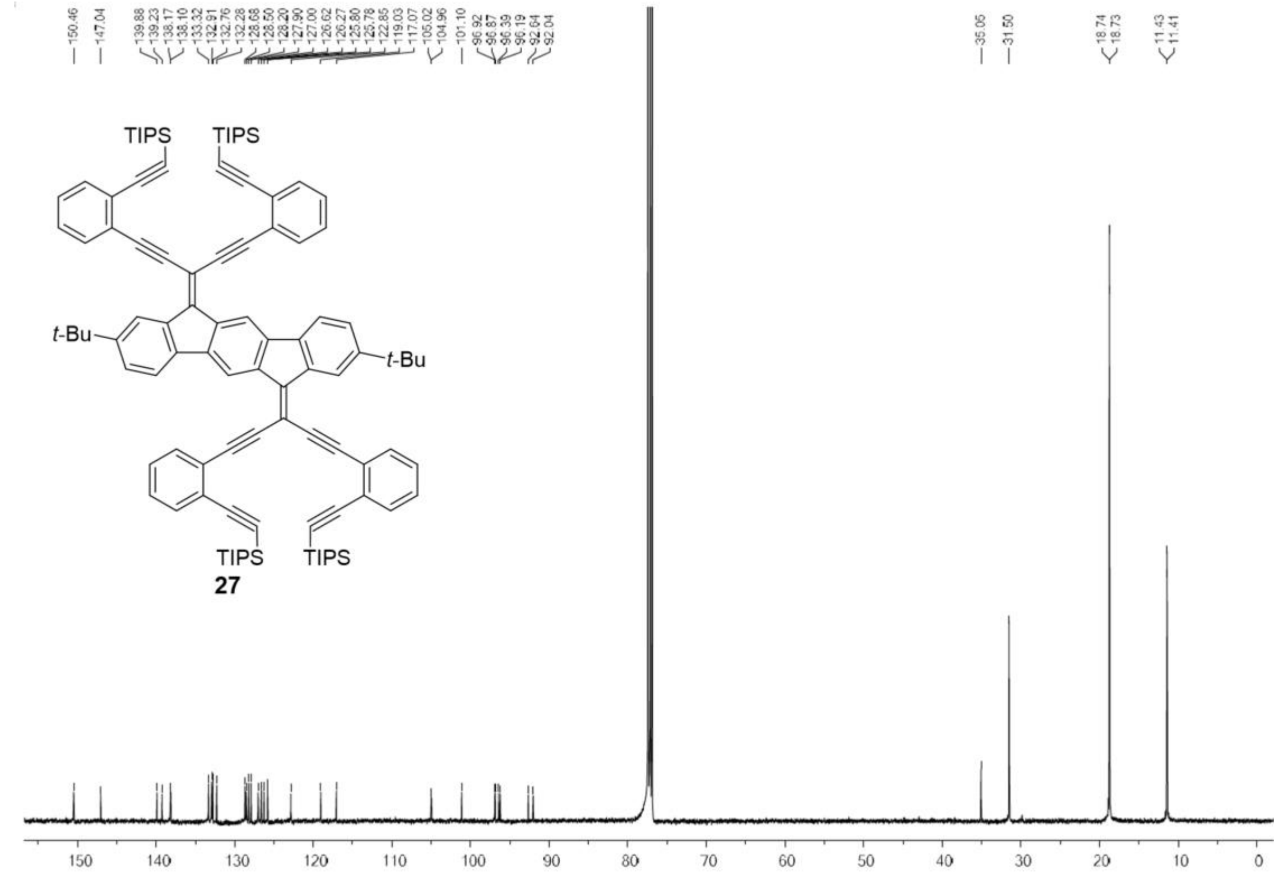

^{13}C NMR spectrum (126 MHz, $CDCl_3$) of **27**.

Electrochemistry

Compounds **11** and **15** were studied in MeCN and compounds **13**, **16**, **17**, **22**, **23**, **26**, and **27** in CH_2Cl_2 (all measurements with 0.1 M Bu_4NPF_6 as supporting electrolyte).

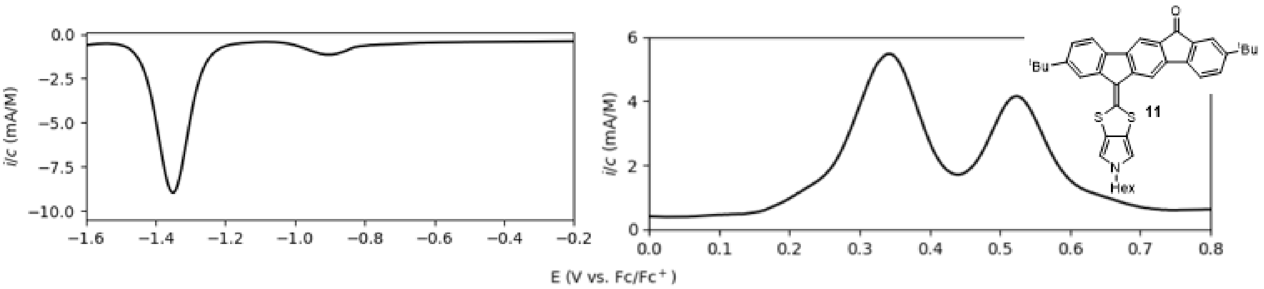

Figure S5. Differential pulse voltammograms of **11**.

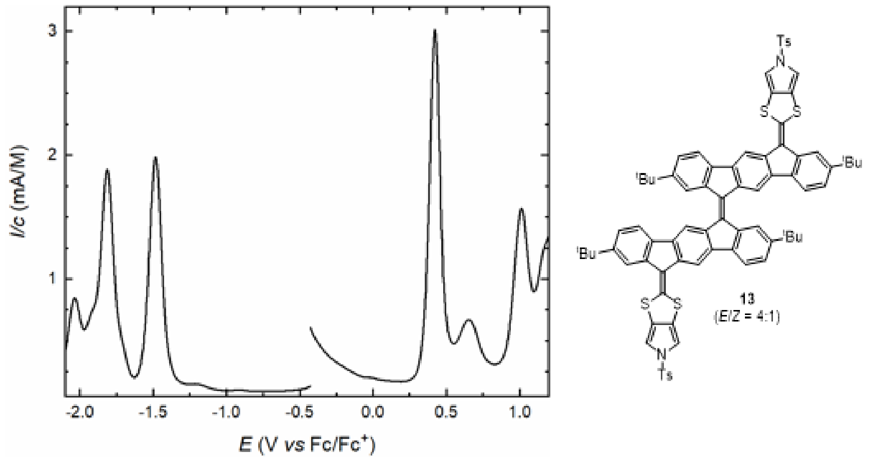

Figure S6. Differential pulse voltammograms of **13**.

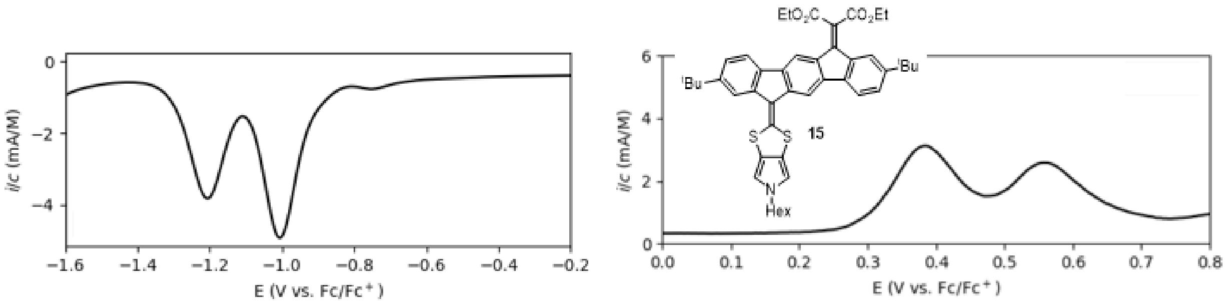

Figure S7. Differential pulse voltammograms of **15**.

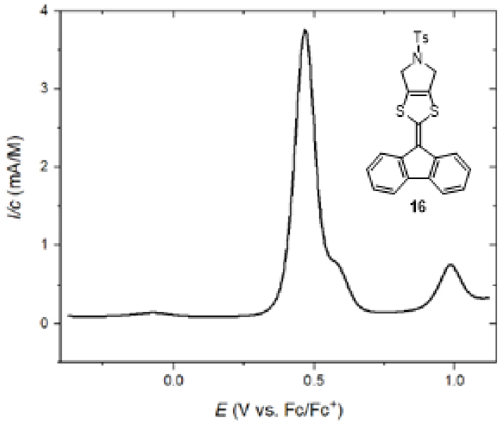

Figure S8. Differential pulse voltammogram of **16**.

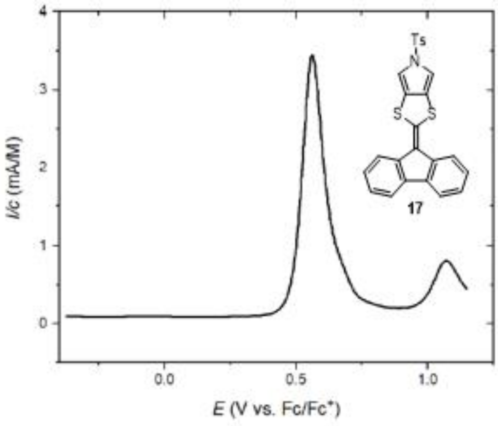

Figure S9. Differential pulse voltammogram of **17**.

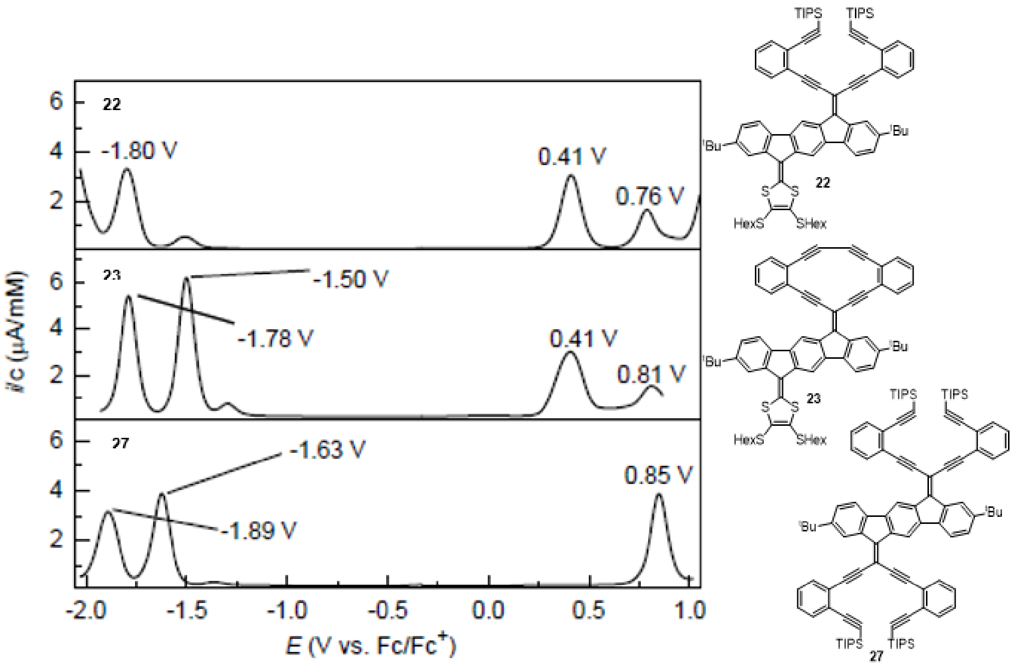

Figure S10. Differential pulse voltammograms of **22**, **23**, and **27**.

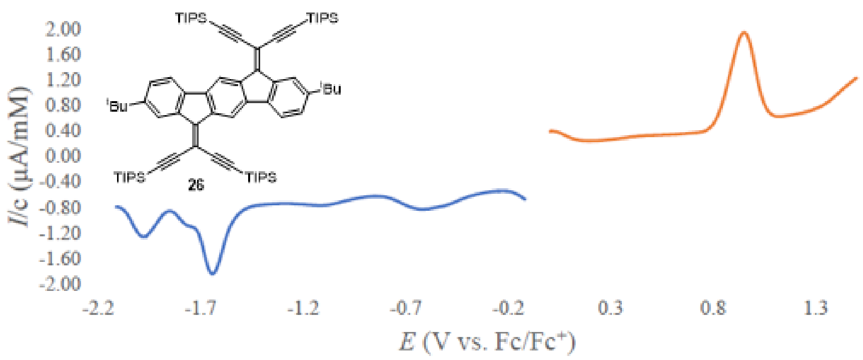

Figure S11. Differential pulse voltammograms of **26**.

X-ray crystallographic data

X-ray crystallographic data for compound 25

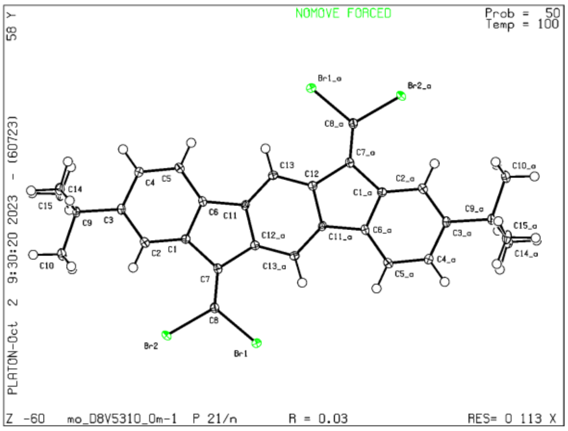

A yellow, Prism-shaped crystal of **25** was mounted on a MiTeGen micromount with perfluoroether oil. Data were collected from a shock-cooled single crystal at 100(2) K on a Bruker D8 VENTURE dual wavelength Mo/Cu three-circle diffractometer with a microfocus sealed X-ray tube using a mirror optics as monochromator and a Bruker PHOTON II detector. The diffractometer was equipped with an Oxford Cryostream 800 low temperature device and used MoK_α radiation (λ = 0.71073 Å). All data were integrated with SAINT and a multi-scan absorption correction using SADABS was applied.[2,3] The structure was solved by direct methods using SHELXT and refined by full-matrix least-squares methods against F^2 by SHELXL-2019/2.[4,5] All non-hydrogen atoms were refined with anisotropic displacement parameters. All hydrogen atoms were refined isotropic on calculated positions using a riding model with their U_{iso} values constrained to 1.5 times the U_{eq} of their pivot atoms for terminal sp^3 carbon atoms and 1.2 times for all other carbon atoms. Crystallographic data for the structures reported here have been deposited with the Cambridge Crystallographic Data Centre.[6] CCDC 2298562 contains the supplementary crystallographic data for this paper. These data can be obtained free of charge from The Cambridge Crystallographic Data Centre via www.ccdc.cam.ac.uk/structures. This report and the CIF file were generated using FinalCif.[7]

Table S1. Crystal data and structure refinement for 25

CCDC number	2298652
Empirical formula	$C_{30}H_{26}Br_4$
Formula weight	706.15
Temperature [K]	100(2)
Crystal system	monoclinic
Space group (number)	$P2_1/n$ (14)
a [Å]	13.2258(8)
b [Å]	7.3391(4)
c [Å]	13.3672(7)
α [°]	90
β [°]	95.467(2)
γ [°]	90
Volume [Å^3]	1291.59(13)
Z	2
ρ_{calc} [gcm^{-3}]	1.816
μ [mm^{-1}]	6.250
F(000)	692
Crystal size [mm^3]	0.198×0.157×0.057
Crystal colour	yellow
Crystal shape	Prism
Radiation	MoK_α (λ=0.71073 Å)
2θ range [°]	4.55 to 57.40 (0.74 Å)
Index ranges	−17 ≤ h ≤ 17 −9 ≤ k ≤ 9 −18 ≤ l ≤ 14
Reflections collected	27084
Independent reflections	3334 R_{int} = 0.0635 R_{sigma} = 0.0364
Completeness to θ = 25.242°	99.9 %

Data / Restraints / Parameters	3334/0/157	Final R indexes [all data]	R_1 = 0.0321 wR_2 = 0.0602
Goodness-of-fit on F^2	1.080	Largest peak/hole [eÅ^{-3}]	0.51/-0.64
Final R indexes [I≥2σ(I)]	R_1 = 0.0251 wR_2 = 0.0579		

Table S2. Atomic coordinates and U_{eq} [Å^2] for 25

Atom	*x*	*y*	*z*	U_{eq}
Br1	0.29892(2)	0.63650(3)	0.30213(2)	0.01543(7)
C1	0.42044(14)	0.6095(2)	0.61240(15)	0.0093(4)
Br2	0.27288(2)	0.33569(3)	0.45940(2)	0.01515(7)
C2	0.39622(14)	0.4607(3)	0.67120(15)	0.0099(4)
H2	0.360185	0.360102	0.640588	0.012
C3	0.42458(14)	0.4592(2)	0.77425(15)	0.0098(4)
C4	0.48072(15)	0.6061(3)	0.81784(15)	0.0121(4)
H4	0.500465	0.605415	0.888057	0.014
C5	0.50796(15)	0.7526(3)	0.76017(15)	0.0121(4)
H5	0.547176	0.850073	0.790283	0.015
C6	0.47708(14)	0.7546(2)	0.65808(14)	0.0093(4)
C7	0.39576(15)	0.6547(2)	0.50379(15)	0.0090(4)
C8	0.33639(15)	0.5603(3)	0.43508(15)	0.0107(4)
C9	0.39588(15)	0.3015(3)	0.84160(15)	0.0114(4)
C10	0.33549(17)	0.1523(3)	0.78209(17)	0.0163(4)
H10A	0.318839	0.054926	0.827957	0.024
H10B	0.376382	0.102253	0.731176	0.024
H10C	0.272658	0.204144	0.749091	0.024
C11	0.49324(14)	0.8929(3)	0.58244(14)	0.0090(4)

C12	0.55456(15)	1.1656(2)	0.51102(15)	0.0092(4)
C13	0.54697(14)	1.0558(3)	0.59519(14)	0.0104(4)
H13	0.577596	1.091211	0.659426	0.012
C14	0.49348(16)	0.2167(3)	0.89362(16)	0.0154(4)
H14A	0.475682	0.116329	0.937021	0.023
H14B	0.531492	0.309495	0.934294	0.023
H14C	0.535435	0.170132	0.842669	0.023
C15	0.32980(17)	0.3753(3)	0.92187(16)	0.0168(4)
H15A	0.317177	0.277610	0.969085	0.025
H15B	0.264946	0.419227	0.889055	0.025
H15C	0.365369	0.475861	0.958382	0.025

U_{eq} is defined as 1/3 of the trace of the orthogonalized U_{ij} tensor.

Table S3. Anisotropic displacement parameters [Å^2] for 25. The anisotropic displacement factor exponent takes the form: $-2\pi^2 [h^2(a^*)^2U_{11} + k^2(b^*)^2U_{22} + \ldots + 2hka^*b^*U_{12}]$

Atom	U_{11}	U_{22}	U_{33}	U_{23}	U_{13}	U_{12}
Br1	0.02016(12)	0.01511(10)	0.01020(11)	0.00171(7)	−0.00281(8)	−0.00646(7)
C1	0.0103(9)	0.0088(8)	0.0090(9)	−0.0009(7)	0.0018(7)	0.0007(7)
Br2	0.01921(12)	0.01176(10)	0.01395(11)	0.00077(7)	−0.00127(8)	−0.00786(7)
C2	0.0086(9)	0.0086(8)	0.0129(9)	0.0002(7)	0.0018(7)	0.0004(7)
C3	0.0095(9)	0.0090(8)	0.0112(9)	0.0022(7)	0.0027(7)	0.0014(7)
C4	0.0143(10)	0.0121(9)	0.0096(9)	0.0018(7)	0.0005(8)	−0.0015(7)
C5	0.0139(10)	0.0090(9)	0.0133(10)	0.0002(7)	0.0007(8)	−0.0020(7)
C6	0.0091(9)	0.0076(8)	0.0114(9)	0.0013(7)	0.0022(7)	−0.0003(7)
C7	0.0092(9)	0.0073(8)	0.0109(9)	0.0002(7)	0.0027(7)	0.0011(6)
C8	0.0122(9)	0.0092(8)	0.0109(9)	0.0008(7)	0.0020(7)	−0.0001(7)
C9	0.0127(10)	0.0108(8)	0.0107(9)	0.0035(7)	0.0019(7)	−0.0020(7)

C10	0.0187(11)	0.0131(9)	0.0168(11)	0.0047(8)	0.0001(8)	−0.0047(7)
C11	0.0107(9)	0.0093(8)	0.0074(9)	0.0004(7)	0.0033(7)	0.0003(7)
C12	0.0100(9)	0.0086(8)	0.0094(9)	−0.0006(7)	0.0024(7)	0.0004(7)
C13	0.0120(9)	0.0105(8)	0.0087(9)	−0.0013(7)	0.0011(7)	−0.0006(7)
C14	0.0179(10)	0.0117(9)	0.0160(10)	0.0047(8)	−0.0011(8)	−0.0002(8)
C15	0.0188(11)	0.0187(10)	0.0138(10)	0.0046(8)	0.0061(8)	−0.0016(8)

Table S4. Bond lengths and angles for 25.

**Atom–Atom**	**Length [Å]**
Br1–C8	1.884(2)
C1–C2	1.400(3)
C1–C6	1.408(3)
C1–C7	1.495(3)
Br2–C8	1.8923(19)
C2–C3	1.393(3)
C2–H2	0.9500
C3–C4	1.404(3)
C3–C9	1.536(3)
C4–C5	1.391(3)
C4–H4	0.9500
C5–C6	1.387(3)
C5–H5	0.9500
C6–C11	1.463(3)
C7–C8	1.343(3)
C7–C12[#1]	1.495(2)

C9–C10	1.533(3)
C9–C14	1.538(3)
C9–C15	1.545(3)
C10–H10A	0.9800
C10–H10B	0.9800
C10–H10C	0.9800
C11–C13	1.393(3)
C11–C12[#1]	1.412(3)
C12–C13	1.395(3)
C13–H13	0.9500
C14–H14A	0.9800
C14–H14B	0.9800
C14–H14C	0.9800
C15–H15A	0.9800
C15–H15B	0.9800
C15–H15C	0.9800

Atom–Atom–Atom	Angle [°]
C2–C1–C6	119.17(18)
C2–C1–C7	132.69(18)
C6–C1–C7	108.13(16)
C3–C2–C1	120.46(18)
C3–C2–H2	119.8
C1–C2–H2	119.8
C2–C3–C4	119.09(17)
C2–C3–C9	121.84(17)
C4–C3–C9	119.06(17)
C5–C4–C3	121.24(19)
C5–C4–H4	119.4
C3–C4–H4	119.4
C6–C5–C4	119.07(18)
C6–C5–H5	120.5
C4–C5–H5	120.5
C5–C6–C1	120.91(17)
C5–C6–C11	129.69(17)
C1–C6–C11	109.39(17)
C8–C7–C1	127.52(17)
C8–C7–C12	126.90(18)
C1–C7–C12	105.46(16)
C7–C8–Br1	125.19(15)
C7–C8–Br2	124.91(15)
Br1–C8–Br2	109.84(10)
C10–C9–C3	112.32(17)
C10–C9–C14	108.56(16)
C3–C9–C14	109.05(15)
C10–C9–C15	108.04(16)
C3–C9–C15	109.33(16)
C14–C9–C15	109.50(17)
C9–C10–H10A	109.5
C9–C10–H10B	109.5
H10A–C10–H10B	109.5
C9–C10–H10C	109.5
H10A–C10–H10C	109.5
H10B–C10–H10C	109.5
C13–C11–C12	123.31(17)
C13–C11–C6	128.13(18)
C12–C11–C6	108.55(17)
C13–C12–C11	118.53(17)
C13–C12–C7	133.03(18)
C11–C12–C7	108.44(16)
C11–C13–C12	118.16(18)

C11–C13–H13	120.9
C12–C13–H13	120.9
C9–C14–H14A	109.5
C9–C14–H14B	109.5
H14A–C14–H14B	109.5
C9–C14–H14C	109.5
H14A–C14–H14C	109.5
H14B–C14–H14C	109.5
C9–C15–H15A	109.5
C9–C15–H15B	109.5
H15A–C15–H15B	109.5
C9–C15–H15C	109.5
H15A–C15–H15C	109.5
H15B–C15–H15C	109.5

Symmetry transformations used to generate equivalent atoms: #1: 1-X, 2-Y, 1-Z;

Table S5. Torsion angles for 25.

**Atom–Atom–Atom–Atom**	**Torsion Angle [°]**
C6–C1–C2–C3	2.3(3)
C7–C1–C2–C3	−175.90(18)
C1–C2–C3–C4	−2.1(3)
C1–C2–C3–C9	177.71(17)
C2–C3–C4–C5	0.2(3)
C9–C3–C4–C5	−179.55(17)
C3–C4–C5–C6	1.3(3)
C4–C5–C6–C1	−1.1(3)
C4–C5–C6–C11	178.26(19)
C2–C1–C6–C5	−0.7(3)
C7–C1–C6–C5	177.91(17)
C2–C1–C6–C11	179.83(16)
C7–C1–C6–C11	−1.6(2)
C2–C1–C7–C8	4.0(3)
C6–C1–C7–C8	−174.36(19)
C2–C1–C7–C12#1	−179.75(19)
C6–C1–C7–C12#1	1.9(2)
C1–C7–C8–Br1	174.07(14)
C12#1–C7–C8–Br1	−1.4(3)
C1–C7–C8–Br2	−2.9(3)
C12#1–C7–C8–Br2	−178.39(14)
C2–C3–C9–C10	0.6(3)

C4–C3–C9–C10	−179.60(18)
C2–C3–C9–C14	120.99(19)
C4–C3–C9–C14	−59.2(2)
C2–C3–C9–C15	−119.3(2)
C4–C3–C9–C15	60.5(2)
C5–C6–C11–C13	2.0(3)
C1–C6–C11–C13	−178.58(18)
C5–C6–C11–C12$^{\#1}$	−178.83(19)
C1–C6–C11–C12$^{\#1}$	0.6(2)
C12$^{\#1}$–C11–C13–C12	−0.6(3)
C6–C11–C13–C12	178.42(18)
C11$^{\#1}$–C12–C13–C11	0.6(3)
C7$^{\#1}$–C12–C13–C11	179.59(19)

Symmetry transformations used to generate equivalent atoms:

#1: 1-X, 2-Y, 1-Z;

X-ray crystallographic data for compound 26

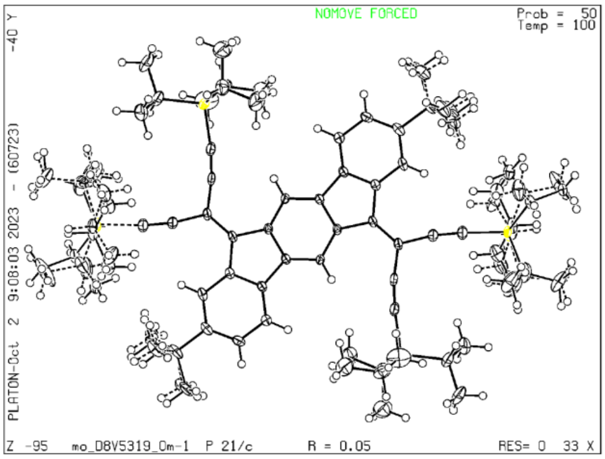

A red, Prism-shaped crystal of **26** was mounted on a MiTeGen micromount with perfluoroether oil. Data were collected from a shock-cooled single crystal at 100(2) K on a Bruker D8 VENTURE dual wavelength Mo/Cu three-circle diffractometer with a microfocus sealed X-ray tube using a mirror optics as monochromator and a Bruker PHOTON II detector. The diffractometer was equipped with an Oxford Cryostream 800 low temperature device and used MoK_{α} radiation (λ = 0.71073 Å). All data were integrated with SAINT and a multi-scan absorption correction using SADABS was applied.[2,3] The structure was solved by direct methods using SHELXT and refined by full-matrix least-squares methods against F^2 by SHELXL-2019/2.[4,5] All non-hydrogen atoms were refined with anisotropic displacement parameters. All hydrogen atoms were refined with isotropic displacement parameters. Some were refined freely and some on calculated positions using a riding model with their U_{iso} values constrained to 1.5 times the U_{eq} of their pivot atoms for terminal sp^3 carbon atoms and 1.2 times for all other carbon atoms. The disordered Si-trisisopropyl groups were modeled as tw parts using SADI, RIGU and SIMU restraints. Two pairs of C-atoms were restrained using EADP due to their high displacement factors. The disordered tert-butyl group was modelled as two parts using SADI, RIGU and SIMU restraints.Crystallographic data for the structures reported here have been deposited with the Cambridge Crystallographic Data Centre.[6] CCDC 2298654 contains the supplementary crystallographic data for this paper. These data can be obtained free of charge from The Cambridge Crystallographic Data Centre via www.ccdc.cam.ac.uk/structures. This report and the CIF file were generated using FinalCif.[7]

Table S6. Crystal data and structure refinement for 26.

CCDC number	2298654
Empirical formula	$C_{74}H_{110}Si_4$
Formula weight	1111.97
Temperature [K]	100(2)
Crystal system	monoclinic
Space group (number)	$P2_1/c$ (14)
a [Å]	17.1710(13)
b [Å]	12.6070(11)
c [Å]	18.4564(15)
α [°]	90
β [°]	117.037(3)
γ [°]	90
Volume [Å^3]	3558.7(5)
Z	2
ρ_{calc} [gcm^{-3}]	1.038
μ [mm^{-1}]	0.121
F(000)	1220
Crystal size [mm^3]	0.193×0.119×0.102
Crystal colour	red
Crystal shape	Prism
Radiation	MoK_{α} (λ=0.71073 Å)
2θ range [°]	4.07 to 53.46 (0.79 Å)
Index ranges	−21 ≤ h ≤ 21<br>−15 ≤ k ≤ 15<br>−23 ≤ l ≤ 23
Reflections collected	66001

Independent reflections	7555 R_{int} = 0.0889 R_{sigma} = 0.0416	Goodness-of-fit on F^2	1.056
Completeness to θ = 25.242°	99.9 %	Final R indexes [I≥2σ(I)]	R_1 = 0.0505 wR_2 = 0.1116
Data / Restraints / Parameters	7555/180/495	Final R indexes [all data]	R_1 = 0.0766 wR_2 = 0.1236
		Largest peak/hole [eÅ^{-3}]	0.41/-0.39

Table S7. Atomic coordinates and U_{eq} [Å^2] for 26.

Atom	*x*	*y*	*z*	U_{eq}
C1	0.10143(11)	0.31813(14)	0.51153(11)	0.0185(4)
Si2	0.38701(3)	0.59837(4)	0.77968(3)	0.02454(14)
C2	0.03976(11)	0.24713(13)	0.45658(10)	0.0164(4)
C3	0.06520(13)	0.14571(14)	0.44642(11)	0.0202(4)
H1C	0.0229(14)	0.0996(17)	0.4096(13)	0.030
C4	0.15256(13)	0.11411(14)	0.49002(11)	0.0234(4)
C5	0.21203(14)	0.18690(17)	0.54253(13)	0.0303(5)
H1B	0.2710(16)	0.1674(19)	0.5704(15)	0.045
C6	0.18773(13)	0.28850(16)	0.55375(13)	0.0284(4)
H1	0.2301(16)	0.3350(19)	0.5923(14)	0.043
C7	0.05680(11)	0.41734(13)	0.51183(10)	0.0167(4)
C8	0.03246(11)	0.59339(13)	0.54361(10)	0.0162(3)
C9	0.04599(11)	0.69892(13)	0.58181(11)	0.0164(4)
C10	0.12129(11)	0.73580(14)	0.64396(11)	0.0190(4)
C11	0.12931(12)	0.83560(15)	0.68468(12)	0.0236(4)
C12	0.14696(13)	0.91324(16)	0.72614(13)	0.0304(5)
C15	0.17895(15)	0.00121(15)	0.47813(13)	0.0311(5)

C17	0.09023(11)	0.50995(14)	0.55550(11)	0.0181(4)
H1AA	0.1491(14)	0.5164(16)	0.5911(13)	0.027
C18	0.20237(12)	0.67874(15)	0.67912(11)	0.0223(4)
C19	0.27379(12)	0.64176(15)	0.71509(12)	0.0259(4)
C20	0.44068(14)	0.5768(2)	0.71240(14)	0.0387(5)
H20	0.502545	0.555043	0.747880	0.046
C21	0.4430(2)	0.6795(3)	0.67013(19)	0.0658(8)
H21A	0.471623	0.666899	0.635544	0.099
H21B	0.475644	0.733347	0.711049	0.099
H21C	0.383108	0.704486	0.636566	0.099
C29	0.38165(15)	0.47717(17)	0.83689(14)	0.0366(5)
H29	0.363467	0.502743	0.878219	0.044
C30	0.31338(18)	0.3960(2)	0.78506(17)	0.0537(7)
H30A	0.257123	0.431561	0.753830	0.081
H30B	0.307234	0.342063	0.820373	0.081
H30C	0.331825	0.362025	0.747603	0.081
C31	0.43939(15)	0.71311(19)	0.85077(14)	0.0425(6)
H31	0.426723	0.777801	0.815871	0.051
C32	0.39801(18)	0.7320(2)	0.90777(16)	0.0598(8)
H32A	0.334761	0.741452	0.875504	0.090
H32B	0.423351	0.795854	0.940319	0.090
H32C	0.409460	0.670809	0.943948	0.090
C33	0.39781(18)	0.4895(3)	0.64939(16)	0.0567(8)
H33A	0.335784	0.506079	0.616161	0.085

H33B	0.403333	0.421558	0.677161	0.085
H33C	0.426764	0.484665	0.614384	0.085
C36	0.47032(18)	0.4244(2)	0.88450(19)	0.0636(8)
H36A	0.493002	0.401814	0.846882	0.095
H36B	0.463983	0.362435	0.913455	0.095
H36C	0.511067	0.474929	0.923804	0.095
C37	0.53924(17)	0.7044(2)	0.89770(18)	0.0639(8)
H37A	0.563138	0.689844	0.859554	0.096
H37B	0.554986	0.646503	0.937325	0.096
H37C	0.563402	0.771199	0.926226	0.096
C16A	0.1372(5)	-0.0763(3)	0.5091(4)	0.0386(14)
H16A	0.073530	-0.069812	0.478490	0.058
H16B	0.155467	-0.062538	0.566839	0.058
H16C	0.154914	-0.148154	0.502589	0.058
C27A	0.1569(6)	-0.0139(10)	0.3879(4)	0.0274(13)
H27A	0.093966	-0.004197	0.353892	0.041
H27B	0.173725	-0.085561	0.379807	0.041
H27C	0.189060	0.038401	0.372615	0.041
C28A	0.2817(3)	-0.0129(4)	0.5276(3)	0.0409(13)
H28A	0.297756	-0.084948	0.519615	0.061
H28B	0.300043	-0.000950	0.585565	0.061
H28C	0.310711	0.038548	0.508148	0.061
C16B	0.0994(6)	-0.0806(6)	0.4708(7)	0.0294(17)
H16D	0.044362	-0.059732	0.424345	0.044

H16E	0.092829	-0.076684	0.520810	0.044
H16F	0.114152	-0.153356	0.463020	0.044
C27B	0.1764(13)	-0.009(2)	0.3966(8)	0.041(3)
H27D	0.118952	0.013759	0.354493	0.061
H27E	0.186808	-0.082687	0.387248	0.061
H27F	0.221818	0.036421	0.394472	0.061
C28B	0.2577(7)	-0.0419(8)	0.5450(7)	0.048(2)
H28D	0.268232	-0.113670	0.531054	0.072
H28E	0.249900	-0.044400	0.594356	0.072
H28F	0.307710	0.003490	0.554358	0.072
C13A	0.1368(8)	0.9862(10)	0.8672(6)	0.029(2)
H13A	0.151448	1.043984	0.908421	0.034
C14A	0.1747(6)	0.8821(10)	0.9133(6)	0.0383(18)
H14A	0.162370	0.824107	0.874168	0.057
H14B	0.147869	0.866357	0.948913	0.057
H14C	0.238093	0.889415	0.946077	0.057
C22A	0.2995(8)	1.0302(16)	0.8492(11)	0.049(5)
H22A	0.316393	0.958074	0.873896	0.059
C23A	0.337(3)	1.032(4)	0.7842(19)	0.063(3)
H23A	0.310954	0.973838	0.745476	0.095
H23B	0.400473	1.024090	0.812267	0.095
H23C	0.321742	1.099902	0.754887	0.095
C24A	0.1479(3)	1.1596(4)	0.7490(3)	0.0230(11)
H24A	0.197592	1.187020	0.740264	0.028

C25A	0.0668(4)	1.1584(4)	0.6668(3)	0.0293(12)
H25A	0.053920	1.230624	0.644973	0.044
H25B	0.017043	1.130871	0.673443	0.044
H25C	0.077324	1.112770	0.629150	0.044
C26A	0.0353(4)	0.9775(5)	0.8205(4)	0.0348(14)
H26A	0.010477	1.046735	0.797475	0.052
H26B	0.012667	0.954854	0.858202	0.052
H26C	0.018852	0.925338	0.776641	0.052
C34A	0.347(3)	1.101(4)	0.918(3)	0.051(3)
H34A	0.345291	1.173546	0.898402	0.076
H34B	0.408285	1.077669	0.947501	0.076
H34C	0.320333	1.099063	0.954945	0.076
C35A	0.1344(4)	1.2372(8)	0.8076(5)	0.0285(15)
H35A	0.121213	1.308210	0.783368	0.043
H35B	0.187814	1.239987	0.859500	0.043
H35C	0.085664	1.212510	0.817093	0.043
Si1A	0.1796(4)	1.0244(6)	0.7973(5)	0.0194(8)
C13B	0.1462(7)	0.9670(9)	0.8880(6)	0.0226(18)
H13B	0.156071	1.026397	0.927090	0.027
C14B	0.2034(6)	0.8726(10)	0.9368(6)	0.040(2)
H14D	0.197227	0.814075	0.899603	0.060
H14E	0.184568	0.849007	0.976948	0.060
H14F	0.264766	0.894841	0.964805	0.060
C22B	0.3053(7)	1.0138(15)	0.8521(9)	0.0208(18)

H22B	0.325338	0.943951	0.880164	0.025
C23B	0.345(3)	1.025(4)	0.7979(19)	0.063(3)
H23D	0.319353	0.972764	0.753827	0.095
H23E	0.408121	1.012631	0.828323	0.095
H23F	0.334335	1.096536	0.774873	0.095
C24B	0.1095(5)	1.1320(4)	0.7542(3)	0.0349(15)
H24B	0.047557	1.106640	0.729571	0.042
C25B	0.1258(6)	1.1746(4)	0.6846(3)	0.047(2)
H25D	0.123833	1.115794	0.649074	0.070
H25E	0.183340	1.208537	0.707005	0.070
H25F	0.080525	1.226595	0.653171	0.070
C26B	0.0502(4)	0.9392(7)	0.8497(4)	0.0400(17)
H26D	0.015426	1.002671	0.824473	0.060
H26E	0.035192	0.912587	0.891685	0.060
H26F	0.037701	0.884474	0.808216	0.060
C34B	0.342(4)	1.102(4)	0.921(3)	0.051(3)
H34D	0.317796	1.171357	0.897742	0.076
H34E	0.405639	1.104153	0.945485	0.076
H34F	0.324021	1.084801	0.963591	0.076
C35B	0.1165(10)	1.2244(11)	0.8103(8)	0.101(5)
H35D	0.069749	1.275610	0.780830	0.152
H35E	0.173405	1.259217	0.828492	0.152
H35F	0.111008	1.197796	0.857644	0.152
Si1B	0.1810(5)	1.0155(6)	0.8089(5)	0.0273(14)

U_{eq} is defined as 1/3 of the trace of the orthogonalized U_{ij} tensor.

Table S8. Anisotropic displacement parameters [Å²] for 26. The anisotropic displacement factor exponent takes the form: $-2\pi^2 [h^2(a^*)^2U_{11} + k^2(b^*)^2U_{22} + \ldots + 2hka^*b^*U_{12}]$

Atom	U_{11}	U_{22}	U_{33}	U_{23}	U_{13}	U_{12}
C1	0.0192(9)	0.0152(8)	0.0234(9)	−0.0034(7)	0.0116(8)	0.0003(7)
Si2	0.0173(3)	0.0230(3)	0.0277(3)	−0.0062(2)	0.0053(2)	−0.0045(2)
C2	0.0183(9)	0.0139(8)	0.0218(9)	−0.0024(7)	0.0133(7)	−0.0024(7)
C3	0.0280(10)	0.0127(8)	0.0262(10)	−0.0036(7)	0.0177(8)	−0.0026(7)
C4	0.0345(11)	0.0164(9)	0.0243(9)	0.0027(7)	0.0178(8)	0.0083(8)
C5	0.0261(10)	0.0297(11)	0.0297(11)	−0.0047(9)	0.0078(9)	0.0129(9)
C6	0.0215(10)	0.0263(10)	0.0310(11)	−0.0108(9)	0.0064(9)	0.0034(8)
C7	0.0161(8)	0.0138(8)	0.0225(9)	−0.0035(7)	0.0108(7)	−0.0009(7)
C8	0.0155(8)	0.0138(8)	0.0223(9)	−0.0050(7)	0.0112(7)	−0.0040(7)
C9	0.0183(8)	0.0122(8)	0.0243(9)	−0.0047(7)	0.0147(7)	−0.0028(7)
C10	0.0194(9)	0.0171(9)	0.0256(9)	−0.0091(7)	0.0147(8)	−0.0054(7)
C11	0.0169(9)	0.0255(10)	0.0318(10)	−0.0116(8)	0.0140(8)	−0.0049(8)
C12	0.0213(10)	0.0302(11)	0.0412(12)	−0.0181(9)	0.0155(9)	−0.0059(8)
C15	0.0471(12)	0.0166(9)	0.0362(11)	0.0031(8)	0.0248(10)	0.0131(8)
C17	0.0130(8)	0.0166(9)	0.0237(9)	−0.0077(7)	0.0073(7)	−0.0028(7)
C18	0.0216(10)	0.0213(9)	0.0262(10)	−0.0147(8)	0.0127(8)	−0.0097(8)
C19	0.0223(10)	0.0251(10)	0.0298(10)	−0.0133(8)	0.0115(9)	−0.0079(8)
C20	0.0215(10)	0.0560(15)	0.0376(12)	−0.0003(11)	0.0124(9)	−0.0004(10)
C21	0.0562(17)	0.084(2)	0.070(2)	0.0195(17)	0.0393(16)	0.0017(16)
C29	0.0418(13)	0.0307(11)	0.0358(12)	−0.0019(9)	0.0165(10)	−0.0033(10)
C30	0.0531(16)	0.0376(14)	0.0643(17)	0.0014(12)	0.0212(14)	−0.0187(12)
C31	0.0323(12)	0.0330(12)	0.0407(13)	−0.0107(10)	−0.0022(10)	−0.0070(10)

C32	0.0594(17)	0.0531(16)	0.0455(15)	−0.0261(13)	0.0051(13)	0.0121(14)
C33	0.0467(15)	0.083(2)	0.0463(15)	−0.0214(14)	0.0263(13)	0.0005(14)
C36	0.0534(17)	0.0399(15)	0.072(2)	0.0127(14)	0.0058(15)	0.0001(13)
C37	0.0396(15)	0.0557(17)	0.0641(18)	−0.0215(14)	−0.0046(13)	−0.0184(13)
C16A	0.065(4)	0.0178(17)	0.042(3)	0.0113(19)	0.033(3)	0.010(2)
C27A	0.031(3)	0.022(3)	0.038(2)	−0.0040(18)	0.023(2)	0.005(2)
C28A	0.052(3)	0.021(2)	0.044(2)	0.0024(16)	0.0175(19)	0.0195(19)
C16B	0.040(3)	0.016(3)	0.026(4)	0.008(3)	0.011(3)	0.009(2)
C27B	0.058(8)	0.027(5)	0.049(3)	0.002(3)	0.035(4)	0.013(5)
C28B	0.050(4)	0.020(4)	0.059(4)	0.004(3)	0.013(3)	0.012(3)
C13A	0.039(3)	0.022(4)	0.030(5)	−0.010(3)	0.021(3)	−0.007(2)
C14A	0.052(5)	0.032(3)	0.044(4)	−0.001(3)	0.034(4)	0.000(3)
C22A	0.053(7)	0.050(10)	0.051(6)	−0.013(5)	0.031(5)	−0.018(5)
C23A	0.042(6)	0.103(6)	0.051(7)	−0.021(7)	0.027(7)	−0.030(5)
C24A	0.029(2)	0.018(2)	0.028(2)	−0.0059(15)	0.0183(18)	−0.0052(17)
C25A	0.036(3)	0.024(2)	0.028(2)	−0.0026(15)	0.0153(19)	−0.0069(19)
C26A	0.038(3)	0.039(3)	0.037(4)	−0.008(2)	0.025(3)	−0.008(2)
C34A	0.043(5)	0.064(2)	0.036(3)	−0.0132(19)	0.010(3)	−0.028(2)
C35A	0.026(2)	0.023(3)	0.031(3)	−0.013(2)	0.008(2)	0.001(2)
Si1A	0.0184(13)	0.0191(14)	0.0194(16)	−0.0101(11)	0.0073(10)	−0.0029(9)
C13B	0.031(3)	0.024(4)	0.022(4)	−0.009(3)	0.019(3)	−0.008(3)
C14B	0.055(5)	0.025(3)	0.046(5)	−0.001(3)	0.028(4)	−0.001(4)
C22B	0.011(3)	0.017(3)	0.026(3)	−0.002(2)	0.000(3)	−0.006(2)
C23B	0.042(6)	0.103(6)	0.051(7)	−0.021(7)	0.027(7)	−0.030(5)

C24B	0.056(4)	0.023(2)	0.035(3)	0.0012(19)	0.029(3)	0.007(2)
C25B	0.082(6)	0.027(3)	0.038(3)	−0.003(2)	0.033(3)	−0.012(3)
C26B	0.034(3)	0.063(5)	0.028(3)	−0.014(3)	0.018(3)	−0.018(3)
C34B	0.043(5)	0.064(2)	0.036(3)	−0.0132(19)	0.010(3)	−0.028(2)
C35B	0.214(14)	0.042(5)	0.054(5)	0.008(4)	0.065(7)	0.057(7)
Si1B	0.0369(17)	0.0155(13)	0.025(2)	−0.0109(13)	0.0102(12)	−0.0004(9)

Table S9. Bond lengths and angles for 26.

**Atom–Atom**	**Length [Å]**
C1–C6	1.376(3)
C1–C2	1.405(2)
C1–C7	1.468(2)
Si2–C19	1.842(2)
Si2–C20	1.872(2)
Si2–C29	1.883(2)
Si2–C31	1.884(2)
C2–C3	1.391(2)
C2–C9$^{\#1}$	1.478(2)
C3–C4	1.400(3)
C3–H1C	0.94(2)
C4–C5	1.387(3)
C4–C15	1.539(2)
C5–C6	1.392(3)
C5–H1B	0.94(2)
C6–H1	0.95(2)
C7–C17	1.386(2)
C7–C8$^{\#1}$	1.410(2)
C8–C17	1.393(2)
C8–C9	1.474(2)
C9–C10	1.362(2)
C10–C18	1.434(3)
C10–C11	1.440(2)
C11–C12	1.194(3)
C12–Si1A	1.826(7)
C12–Si1B	1.877(8)
C15–C28B	1.459(8)
C15–C16A	1.472(4)
C15–C27B	1.491(11)
C15–C27A	1.543(6)
C15–C28A	1.585(5)
C15–C16B	1.668(7)
C17–H1AA	0.93(2)

C18–C19	1.192(3)
C20–C21	1.521(4)
C20–C33	1.526(3)
C20–H20	1.0000
C21–H21A	0.9800
C21–H21B	0.9800
C21–H21C	0.9800
C29–C36	1.522(3)
C29–C30	1.522(3)
C29–H29	1.0000
C30–H30A	0.9800
C30–H30B	0.9800
C30–H30C	0.9800
C31–C32	1.532(4)
C31–C37	1.534(3)
C31–H31	1.0000
C32–H32A	0.9800
C32–H32B	0.9800
C32–H32C	0.9800
C33–H33A	0.9800
C33–H33B	0.9800
C33–H33C	0.9800
C36–H36A	0.9800
C36–H36B	0.9800

C36–H36C	0.9800
C37–H37A	0.9800
C37–H37B	0.9800
C37–H37C	0.9800
C16A–H16A	0.9800
C16A–H16B	0.9800
C16A–H16C	0.9800
C27A–H27A	0.9800
C27A–H27B	0.9800
C27A–H27C	0.9800
C28A–H28A	0.9800
C28A–H28B	0.9800
C28A–H28C	0.9800
C16B–H16D	0.9800
C16B–H16E	0.9800
C16B–H16F	0.9800
C27B–H27D	0.9800
C27B–H27E	0.9800
C27B–H27F	0.9800
C28B–H28D	0.9800
C28B–H28E	0.9800
C28B–H28F	0.9800
C13A–C14A	1.539(10)
C13A–C26A	1.557(12)

C13A–Si1A	1.816(10)
C13A–H13A	1.0000
C14A–H14A	0.9800
C14A–H14B	0.9800
C14A–H14C	0.9800
C22A–C34A	1.46(6)
C22A–C23A	1.60(3)
C22A–Si1A	1.835(11)
C22A–H22A	1.0000
C23A–H23A	0.9800
C23A–H23B	0.9800
C23A–H23C	0.9800
C24A–C25A	1.523(7)
C24A–C35A	1.552(9)
C24A–Si1A	1.885(8)
C24A–H24A	1.0000
C25A–H25A	0.9800
C25A–H25B	0.9800
C25A–H25C	0.9800
C26A–H26A	0.9800
C26A–H26B	0.9800
C26A–H26C	0.9800
C34A–H34A	0.9800
C34A–H34B	0.9800

C34A–H34C	0.9800
C35A–H35A	0.9800
C35A–H35B	0.9800
C35A–H35C	0.9800
C13B–C26B	1.510(11)
C13B–C14B	1.545(9)
C13B–Si1B	1.912(10)
C13B–H13B	1.0000
C14B–H14D	0.9800
C14B–H14E	0.9800
C14B–H14F	0.9800
C22B–C23B	1.45(3)
C22B–C34B	1.59(6)
C22B–Si1B	1.909(9)
C22B–H22B	1.0000
C23B–H23D	0.9800
C23B–H23E	0.9800
C23B–H23F	0.9800
C24B–C35B	1.526(13)
C24B–C25B	1.530(9)
C24B–Si1B	1.887(8)
C24B–H24B	1.0000
C25B–H25D	0.9800
C25B–H25E	0.9800

C25B–H25F	0.9800
C26B–H26D	0.9800
C26B–H26E	0.9800
C26B–H26F	0.9800
C34B–H34D	0.9800
C34B–H34E	0.9800
C34B–H34F	0.9800
C35B–H35D	0.9800
C35B–H35E	0.9800
C35B–H35F	0.9800
**Atom–Atom–Atom**	**Angle [°]**
C6–C1–C2	120.00(16)
C6–C1–C7	131.22(17)
C2–C1–C7	108.78(15)
C19–Si2–C20	107.87(10)
C19–Si2–C29	107.31(10)
C20–Si2–C29	114.72(11)
C19–Si2–C31	104.60(9)
C20–Si2–C31	110.51(11)
C29–Si2–C31	111.22(11)
C3–C2–C1	120.19(16)
C3–C2–C9	131.60(16)

C1–C2–C9	108.21(14)
C2–C3–C4	120.28(17)
C2–C3–H1C	118.7(13)
C4–C3–H1C	121.0(13)
C5–C4–C3	118.01(17)
C5–C4–C15	122.70(18)
C3–C4–C15	119.29(17)
C4–C5–C6	122.52(19)
C4–C5–H1B	118.6(15)
C6–C5–H1B	118.9(15)
C1–C6–C5	118.97(18)
C1–C6–H1	121.1(14)
C5–C6–H1	119.9(14)
C17–C7–C8	121.93(15)
C17–C7–C1	129.85(16)
C8–C7–C1	108.22(14)
C17–C8–C7	120.21(15)
C17–C8–C9	131.31(16)
C7–C8–C9	108.46(15)
C10–C9–C8	126.43(16)
C10–C9–C2	127.16(15)
C8–C9–C2	106.26(14)
C9–C10–C18	124.38(16)
C9–C10–C11	124.54(17)

C18–C10–C11	111.06(15)
C12–C11–C10	170.9(2)
C11–C12–Si1A	174.9(3)
C11–C12–Si1B	168.3(3)
C28B–C15–C27B	114.4(10)
C28B–C15–C4	116.3(4)
C16A–C15–C4	109.4(2)
C27B–C15–C4	110.2(10)
C16A–C15–C27A	114.4(5)
C4–C15–C27A	108.9(5)
C16A–C15–C28A	108.2(3)
C4–C15–C28A	110.1(2)
C27A–C15–C28A	105.7(4)
C28B–C15–C16B	104.4(5)
C27B–C15–C16B	102.7(9)
C4–C15–C16B	107.4(3)
C7–C17–C8	117.86(16)
C7–C17–H1AA	121.1(13)
C8–C17–H1AA	121.0(13)

C19–C18–C10	171.70(19)
C18–C19–Si2	172.85(17)
C21–C20–C33	109.8(2)
C21–C20–Si2	110.59(18)
C33–C20–Si2	113.44(16)
C21–C20–H20	107.6
C33–C20–H20	107.6
Si2–C20–H20	107.6
C20–C21–H21A	109.5
C20–C21–H21B	109.5
H21A–C21–H21B	109.5
C20–C21–H21C	109.5
H21A–C21–H21C	109.5
H21B–C21–H21C	109.5
C36–C29–C30	110.6(2)
C36–C29–Si2	112.79(17)
C30–C29–Si2	114.41(16)
C36–C29–H29	106.1
C30–C29–H29	106.1
Si2–C29–H29	106.1
C29–C30–H30A	109.5

C29–C30–H30B	109.5
H30A–C30–H30B	109.5
C29–C30–H30C	109.5
H30A–C30–H30C	109.5
H30B–C30–H30C	109.5
C32–C31–C37	111.8(2)
C32–C31–Si2	111.96(17)
C37–C31–Si2	113.29(17)
C32–C31–H31	106.4
C37–C31–H31	106.4
Si2–C31–H31	106.4
C31–C32–H32A	109.5
C31–C32–H32B	109.5
H32A–C32–H32B	109.5
C31–C32–H32C	109.5
H32A–C32–H32C	109.5
H32B–C32–H32C	109.5
C20–C33–H33A	109.5
C20–C33–H33B	109.5

H33A–C33–H33B	109.5
C20–C33–H33C	109.5
H33A–C33–H33C	109.5
H33B–C33–H33C	109.5
C29–C36–H36A	109.5
C29–C36–H36B	109.5
H36A–C36–H36B	109.5
C29–C36–H36C	109.5
H36A–C36–H36C	109.5
H36B–C36–H36C	109.5
C31–C37–H37A	109.5
C31–C37–H37B	109.5
H37A–C37–H37B	109.5
C31–C37–H37C	109.5
H37A–C37–H37C	109.5
H37B–C37–H37C	109.5

C15–C16A–H16A	109.5
C15–C16A–H16B	109.5
H16A–C16A–H16B	109.5
C15–C16A–H16C	109.5
H16A–C16A–H16C	109.5
H16B–C16A–H16C	109.5
C15–C27A–H27A	109.5
C15–C27A–H27B	109.5
H27A–C27A–H27B	109.5
C15–C27A–H27C	109.5
H27A–C27A–H27C	109.5
H27B–C27A–H27C	109.5

C15–C28A–H28A	109.5
C15–C28A–H28B	109.5
H28A–C28A–H28B	109.5
C15–C28A–H28C	109.5
H28A–C28A–H28C	109.5
H28B–C28A–H28C	109.5
C15–C16B–H16D	109.5
C15–C16B–H16E	109.5
H16D–C16B–H16E	109.5
C15–C16B–H16F	109.5
H16D–C16B–H16F	109.5
H16E–C16B–H16F	109.5

C15–C27B–H27D	109.5
C15–C27B–H27E	109.5
H27D–C27B–H27E	109.5
C15–C27B–H27F	109.5
H27D–C27B–H27F	109.5
H27E–C27B–H27F	109.5
C15–C28B–H28D	109.5
C15–C28B–H28E	109.5
H28D–C28B–H28E	109.5
C15–C28B–H28F	109.5
H28D–C28B–H28F	109.5
H28E–C28B–H28F	109.5

C14A–C13A–C26A	109.4(7)
C14A–C13A–Si1A	114.4(9)
C26A–C13A–Si1A	109.8(6)
C14A–C13A–H13A	107.7
C26A–C13A–H13A	107.7
Si1A–C13A–H13A	107.7
C13A–C14A–H14A	109.5
C13A–C14A–H14B	109.5
H14A–C14A–H14B	109.5
C13A–C14A–H14C	109.5
H14A–C14A–H14C	109.5
H14B–C14A–H14C	109.5

C34A–C22A–C23A	113(3)
C34A–C22A–Si1A	122(2)
C23A–C22A–Si1A	110.3(18)
C34A–C22A–H22A	103.0
C23A–C22A–H22A	103.0
Si1A–C22A–H22A	103.0
C22A–C23A–H23A	109.5
C22A–C23A–H23B	109.5
H23A–C23A–H23B	109.5
C22A–C23A–H23C	109.5
H23A–C23A–H23C	109.5
H23B–C23A–H23C	109.5

C25A–C24A–C35A	110.1(4)
C25A–C24A–Si1A	113.5(4)
C35A–C24A–Si1A	110.0(5)
C25A–C24A–H24A	107.7
C35A–C24A–H24A	107.7
Si1A–C24A–H24A	107.7
C24A–C25A–H25A	109.5
C24A–C25A–H25B	109.5
H25A–C25A–H25B	109.5
C24A–C25A–H25C	109.5
H25A–C25A–H25C	109.5
H25B–C25A–H25C	109.5

C13A–C26A–H26A	109.5
C13A–C26A–H26B	109.5
H26A–C26A–H26B	109.5
C13A–C26A–H26C	109.5
H26A–C26A–H26C	109.5
H26B–C26A–H26C	109.5
C22A–C34A–H34A	109.5
C22A–C34A–H34B	109.5
H34A–C34A–H34B	109.5
C22A–C34A–H34C	109.5
H34A–C34A–H34C	109.5
H34B–C34A–H34C	109.5

C24A–C35A–H35A	109.5
C24A–C35A–H35B	109.5
H35A–C35A–H35B	109.5
C24A–C35A–H35C	109.5
H35A–C35A–H35C	109.5
H35B–C35A–H35C	109.5
C13A–Si1A–C12	102.4(6)
C13A–Si1A–C22A	111.2(9)
C12–Si1A–C22A	108.1(7)
C13A–Si1A–C24A	116.9(5)
C12–Si1A–C24A	115.2(4)
C22A–Si1A–C24A	103.0(7)
C26B–C13B–C14B	111.4(7)
C26B–C13B–Si1B	111.8(6)

C14B–C13B–Si1B	110.8(8)
C26B–C13B–H13B	107.6
C14B–C13B–H13B	107.6
Si1B–C13B–H13B	107.6
C13B–C14B–H14D	109.5
C13B–C14B–H14E	109.5
H14D–C14B–H14E	109.5
C13B–C14B–H14F	109.5
H14D–C14B–H14F	109.5
H14E–C14B–H14F	109.5
C23B–C22B–C34B	111(3)
C23B–C22B–Si1B	119.8(19)

C34B–C22B–Si1B	106(2)
C23B–C22B–H22B	106.4
C34B–C22B–H22B	106.4
Si1B–C22B–H22B	106.4
C22B–C23B–H23D	109.5
C22B–C23B–H23E	109.5
H23D–C23B–H23E	109.5
C22B–C23B–H23F	109.5
H23D–C23B–H23F	109.5
H23E–C23B–H23F	109.5
C35B–C24B–C25B	108.3(7)
C35B–C24B–Si1B	113.5(7)

C25B–C24B–Si1B	113.5(5)
C35B–C24B–H24B	107.0
C25B–C24B–H24B	107.0
Si1B–C24B–H24B	107.0
C24B–C25B–H25D	109.5
C24B–C25B–H25E	109.5
H25D–C25B–H25E	109.5
C24B–C25B–H25F	109.5
H25D–C25B–H25F	109.5
H25E–C25B–H25F	109.5
C13B–C26B–H26D	109.5
C13B–C26B–H26E	109.5

H26D–C26B–H26E	109.5
C13B–C26B–H26F	109.5
H26D–C26B–H26F	109.5
H26E–C26B–H26F	109.5
C22B–C34B–H34D	109.5
C22B–C34B–H34E	109.5
H34D–C34B–H34E	109.5
C22B–C34B–H34F	109.5
H34D–C34B–H34F	109.5
H34E–C34B–H34F	109.5
C24B–C35B–H35D	109.5
C24B–C35B–H35E	109.5

H35D–C35B–H35E	109.5
C24B–C35B–H35F	109.5
H35D–C35B–H35F	109.5
H35E–C35B–H35F	109.5
C12–Si1B–C24B	102.3(4)
C12–Si1B–C22B	102.0(7)
C24B–Si1B–C22B	124.4(7)
C12–Si1B–C13B	108.1(6)
C24B–Si1B–C13B	107.7(5)
C22B–Si1B–C13B	110.8(8)

Symmetry transformations used to generate equivalent atoms:

#1: -X, 1-Y, 1-Z;

Table S10. Torsion angles for 26.

Atom–Atom–Atom–Atom	Torsion Angle [°]
C6–C1–C2–C3	1.9(3)
C7–C1–C2–C3	−178.43(16)
C6–C1–C2–C9$^{\#1}$	−177.91(17)
C7–C1–C2–C9$^{\#1}$	1.79(19)
C1–C2–C3–C4	−0.8(3)
C9$^{\#1}$–C2–C3–C4	178.87(17)
C2–C3–C4–C5	−0.4(3)
C2–C3–C4–C15	179.59(17)
C3–C4–C5–C6	0.7(3)
C15–C4–C5–C6	−179.3(2)
C2–C1–C6–C5	−1.6(3)
C7–C1–C6–C5	178.8(2)
C4–C5–C6–C1	0.3(3)
C6–C1–C7–C17	0.1(3)
C2–C1–C7–C17	−179.52(18)
C6–C1–C7–C8$^{\#1}$	179.4(2)
C2–C1–C7–C8$^{\#1}$	−0.3(2)
C17–C8–C9–C10	−4.7(3)
C7$^{\#1}$–C8–C9–C10	173.49(17)
C17–C8–C9–C2$^{\#1}$	179.36(18)
C7$^{\#1}$–C8–C9–C2$^{\#1}$	−2.40(19)

C8–C9–C10–C18	4.2(3)
C2#1–C9–C10–C18	179.29(17)
C8–C9–C10–C11	−173.72(17)
C2#1–C9–C10–C11	1.3(3)
C5–C4–C15–C28B	24.0(7)
C3–C4–C15–C28B	−156.0(7)
C5–C4–C15–C16A	113.9(4)
C3–C4–C15–C16A	−66.1(4)
C5–C4–C15–C27B	−108.3(8)
C3–C4–C15–C27B	71.7(8)
C5–C4–C15–C27A	−120.3(4)
C3–C4–C15–C27A	59.6(4)
C5–C4–C15–C28A	−4.9(4)
C3–C4–C15–C28A	175.1(3)
C5–C4–C15–C16B	140.5(4)
C3–C4–C15–C16B	−39.5(4)
C8#1–C7–C17–C8	0.5(3)
C1–C7–C17–C8	179.65(17)
C7#1–C8–C17–C7	−0.5(3)
C9–C8–C17–C7	177.58(17)
C19–Si2–C20–C21	61.4(2)
C29–Si2–C20–C21	−179.11(17)
C31–Si2–C20–C21	−52.4(2)
C19–Si2–C20–C33	−62.5(2)

C29–Si2–C20–C33	57.0(2)
C31–Si2–C20–C33	−176.32(18)
C19–Si2–C29–C36	172.53(18)
C20–Si2–C29–C36	52.7(2)
C31–Si2–C29–C36	−73.6(2)
C19–Si2–C29–C30	45.0(2)
C20–Si2–C29–C30	−74.8(2)
C31–Si2–C29–C30	158.84(18)
C19–Si2–C31–C32	65.06(19)
C20–Si2–C31–C32	−179.10(17)
C29–Si2–C31–C32	−50.5(2)
C19–Si2–C31–C37	−167.4(2)
C20–Si2–C31–C37	−51.5(2)
C29–Si2–C31–C37	77.1(2)
C14A–C13A–Si1A–C12	61.0(8)
C26A–C13A–Si1A–C12	−62.4(8)
C14A–C13A–Si1A–C22A	−54.3(11)
C26A–C13A–Si1A–C22A	−177.7(9)
C14A–C13A–Si1A–C24A	−172.1(6)

C26A–C13A–Si1A–C24A	64.5(9)
C34A–C22A–Si1A–C13A	−60(3)
C23A–C22A–Si1A–C13A	164(2)
C34A–C22A–Si1A–C12	−171(2)
C23A–C22A–Si1A–C12	53(2)
C34A–C22A–Si1A–C24A	66(3)
C23A–C22A–Si1A–C24A	−70(2)
C25A–C24A–Si1A–C13A	−93.0(7)
C35A–C24A–Si1A–C13A	30.8(7)
C25A–C24A–Si1A–C12	27.4(6)
C35A–C24A–Si1A–C12	151.2(4)
C25A–C24A–Si1A–C22A	144.8(8)

C35A–C24A–Si1A–C22A	−91.3(8)
C11–C12–Si1B–C24B	−150.6(13)
C11–C12–Si1B–C22B	79.8(16)
C11–C12–Si1B–C13B	−37.1(17)
C35B–C24B–Si1B–C12	173.6(7)
C25B–C24B–Si1B–C12	−62.1(7)
C35B–C24B–Si1B–C22B	−72.3(11)
C25B–C24B–Si1B–C22B	51.9(10)
C35B–C24B–Si1B–C13B	59.8(9)
C25B–C24B–Si1B–C13B	−176.0(6)

Symmetry transformations used to generate equivalent atoms:

#1: -X, 1-Y, 1-Z;

X-ray crystallographic data for compound 29

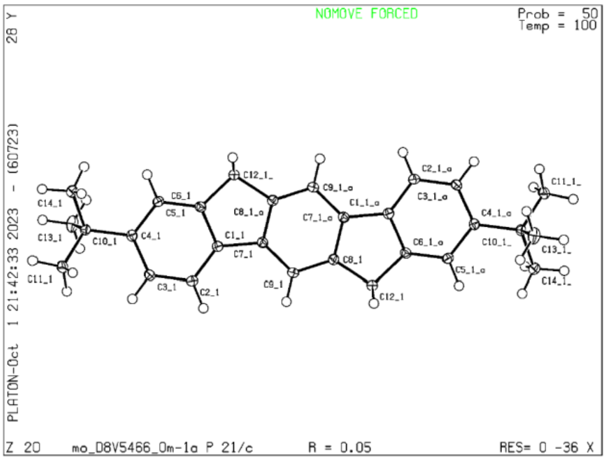

A yellow, Prism-shaped crystal of **29** was mounted on a MiTeGen micromount with perfluoroether oil. Data were collected from a shock-cooled single crystal at 100(2) K on a Bruker D8 VENTURE dual wavelength Mo/Cu three-circle diffractometer with a microfocus sealed X-ray tube using a mirror optics as monochromator and a Bruker PHOTON II detector. The diffractometer was equipped with an Oxford Cryostream 800 low temperature device and used MoK_α radiation (λ = 0.71073 Å). All data were integrated with SAINT and a multi-scan absorption correction using SADABS was applied.[2,3] The structure was solved by direct methods using SHELXT and refined by full-matrix least-squares methods against F^2 by SHELXL-2019/2.[4,5] All non-hydrogen atoms were refined with anisotropic displacement parameters. All hydrogen atoms were refined isotropic on calculated positions using a riding model with their U_{iso} values constrained to 1.5 times the U_{eq} of their pivot atoms for terminal sp^3 carbon atoms and 1.2 times for all other carbon atoms. Crystallographic data for the structures reported here have been deposited with the Cambridge Crystallographic Data Centre.[6] CCDC 2298651 contains the supplementary crystallographic data for this paper. These data can be obtained free of charge from The Cambridge Crystallographic Data Centre via www.ccdc.cam.ac.uk/structures. This report and the CIF file were generated using FinalCif.[7]

Table S10. Crystal data and structure refinement for 29.

CCDC number	2298651
Empirical formula	$C_{28}H_{30}$
Formula weight	366.52
Temperature [K]	100(2)
Crystal system	monoclinic
Space group (number)	$P2_1/c$ (14)
a [Å]	16.0713(8)
b [Å]	5.9999(3)
c [Å]	10.4377(5)
α [°]	90
β [°]	92.484(2)
γ [°]	90
Volume [Å^3]	1005.52(9)
Z	2
ρ_{calc} [gcm^{-3}]	1.211
μ [mm^{-1}]	0.068
F(000)	396
Crystal size [mm^3]	0.219×0.177×0.173
Crystal colour	yellow
Crystal shape	Prism
Radiation	MoK_α (λ=0.71073 Å)
2θ range [°]	5.07 to 60.02 (0.71 Å)
Index ranges	$-22 \le h \le 22$ $-8 \le k \le 8$ $-14 \le l \le 14$
Reflections collected	26009
Independent reflections	2946 R_{int} = 0.0642 R_{sigma} = 0.0365
Completeness to θ = 25.242°	99.7 %
Data / Restraints / Parameters	2946/0/130
Goodness-of-fit on F^2	1.038
Final *R* indexes [$I \ge 2\sigma(I)$]	R_1 = 0.0477 wR_2 = 0.1152

Final R indexes [all data]	R_1 = 0.0670<br>wR_2 = 0.1250	Largest peak/hole [eÅ^{-3}] 0.40/-0.24

Table S12. Atomic coordinates and U_{eq} [Å^2] for 29.

Atom	*x*	*y*	*z*	U_{eq}
C1_1	0.37323(7)	0.42327(18)	0.67566(10)	0.0123(2)
C2_1	0.35447(7)	0.26514(19)	0.76761(11)	0.0145(2)
H2_1	0.390867	0.142983	0.784831	0.017
C3_1	0.28160(7)	0.28830(19)	0.83411(10)	0.0143(2)
H3_1	0.268773	0.179677	0.896469	0.017
C4_1	0.22656(7)	0.46733(18)	0.81174(10)	0.0120(2)
C5_1	0.24746(7)	0.62659(19)	0.72008(10)	0.0135(2)
H5_1	0.211718	0.750362	0.703608	0.016
C6_1	0.31967(7)	0.60515(18)	0.65340(10)	0.0129(2)
C7_1	0.44327(7)	0.44208(18)	0.59044(10)	0.0123(2)
C8_1	0.56725(7)	0.36132(19)	0.48252(10)	0.0125(2)
C9_1	0.51099(7)	0.30090(19)	0.57375(10)	0.0136(2)
H9_1	0.518328	0.168547	0.622910	0.016
C10_1	0.14528(7)	0.48775(19)	0.88233(10)	0.0129(2)
C11_1	0.15002(8)	0.3645(2)	1.01127(11)	0.0184(3)
H11A_1	0.099596	0.395180	1.057781	0.028
H11B_1	0.198958	0.415824	1.062280	0.028
H11C_1	0.154544	0.203879	0.996096	0.028
C12_1	0.64618(7)	0.24406(19)	0.44759(11)	0.0153(2)
H12A_1	0.634131	0.093489	0.412712	0.018
H12B_1	0.685600	0.230986	0.522764	0.018

C13_1	0.07415(7)	0.3858(2)	0.79820(12)	0.0201(3)
H13A_1	0.021736	0.398780	0.842224	0.030
H13B_1	0.086071	0.228181	0.782513	0.030
H13C_1	0.069317	0.465424	0.716238	0.030
C14_1	0.12359(7)	0.7330(2)	0.90859(11)	0.0168(2)
H14A_1	0.073207	0.739939	0.958079	0.025
H14B_1	0.113853	0.811444	0.826968	0.025
H14C_1	0.169885	0.803625	0.957429	0.025

U_{eq} is defined as 1/3 of the trace of the orthogonalized U_{ij} tensor.

Table S13. Anisotropic displacement parameters [Å^2] for 29. The anisotropic displacement factor exponent takes the form: $-2\pi^2 [h^2(a^*)^2U_{11} + k^2(b^*)^2U_{22} + \ldots + 2hka^*b^*U_{12}]$

Atom	U_{11}	U_{22}	U_{33}	U_{23}	U_{13}	U_{12}
C1_1	0.0133(5)	0.0127(5)	0.0108(5)	−0.0005(4)	0.0000(4)	0.0005(4)
C2_1	0.0160(5)	0.0125(5)	0.0152(5)	0.0025(4)	0.0017(4)	0.0034(4)
C3_1	0.0172(5)	0.0129(5)	0.0130(5)	0.0019(4)	0.0035(4)	0.0008(4)
C4_1	0.0130(5)	0.0124(5)	0.0106(5)	−0.0015(4)	0.0011(4)	−0.0009(4)
C5_1	0.0143(5)	0.0126(5)	0.0137(5)	0.0006(4)	0.0013(4)	0.0028(4)
C6_1	0.0144(5)	0.0127(5)	0.0117(5)	0.0007(4)	0.0010(4)	0.0003(4)
C7_1	0.0129(5)	0.0127(5)	0.0113(5)	0.0005(4)	0.0006(4)	0.0000(4)
C8_1	0.0127(5)	0.0122(5)	0.0125(5)	−0.0002(4)	0.0013(4)	0.0003(4)
C9_1	0.0152(5)	0.0121(5)	0.0136(5)	0.0023(4)	0.0013(4)	0.0010(4)
C10_1	0.0132(5)	0.0122(5)	0.0136(5)	−0.0004(4)	0.0031(4)	0.0001(4)
C11_1	0.0234(6)	0.0172(6)	0.0152(5)	0.0021(4)	0.0081(4)	0.0038(5)
C12_1	0.0152(5)	0.0146(5)	0.0165(5)	0.0044(4)	0.0041(4)	0.0037(4)
C13_1	0.0157(5)	0.0248(6)	0.0199(6)	−0.0069(5)	0.0026(4)	−0.0029(5)

C14_1	0.0182(5)	0.0142(5)	0.0184(5)	−0.0003(4)	0.0054(4)	0.0024(4)

Table S14. Bond lengths and angles for 29.

Atom–Atom	Length [Å]
C1_1–C2_1	1.3919(15)
C1_1–C6_1	1.4030(15)
C1_1–C7_1	1.4690(15)
C2_1–C3_1	1.3940(15)
C2_1–H2_1	0.9500
C3_1–C4_1	1.4046(15)
C3_1–H3_1	0.9500
C4_1–C5_1	1.4032(15)
C4_1–C10_1	1.5322(15)
C5_1–C6_1	1.3849(15)
C5_1–H5_1	0.9500
C6_1–C12_1[#1]	1.5102(15)
C7_1–C9_1	1.3960(15)
C7_1–C8_1[#1]	1.4103(15)
C8_1–C9_1	1.3894(15)
C8_1–C12_1	1.5090(15)
C9_1–H9_1	0.9500
C10_1–C11_1	1.5345(15)
C10_1–C13_1	1.5380(16)
C10_1–C14_1	1.5393(16)
C11_1–H11A_1	0.9800
C11_1–H11B_1	0.9800
C11_1–H11C_1	0.9800
C12_1–H12A_1	0.9900
C12_1–H12B_1	0.9900
C13_1–H13A_1	0.9800
C13_1–H13B_1	0.9800
C13_1–H13C_1	0.9800
C14_1–H14A_1	0.9800
C14_1–H14B_1	0.9800
C14_1–H14C_1	0.9800
**Atom–Atom–Atom**	**Angle [°]**
C2_1–C1_1–C6_1	119.65(10)
C2_1–C1_1–C7_1	131.64(10)
C6_1–C1_1–C7_1	108.70(9)
C1_1–C2_1–C3_1	119.15(10)

C1_1–C2_1–H2_1	120.4
C3_1–C2_1–H2_1	120.4
C2_1–C3_1–C4_1	122.01(10)
C2_1–C3_1–H3_1	119.0
C4_1–C3_1–H3_1	119.0
C5_1–C4_1–C3_1	117.81(10)
C5_1–C4_1–C10_1	120.51(10)
C3_1–C4_1–C10_1	121.67(10)
C6_1–C5_1–C4_1	120.66(10)
C6_1–C5_1–H5_1	119.7
C4_1–C5_1–H5_1	119.7
C5_1–C6_1–C1_1	120.70(10)

C5_1–C6_1–C12_1	129.26(10)
C1_1–C6_1–C12_1	110.04(9)
C9_1–C7_1–C8_1	121.07(10)
C9_1–C7_1–C1_1	130.61(10)
C8_1–C7_1–C1_1	108.31(9)
C9_1–C8_1–C7_1	121.47(10)
C9_1–C8_1–C12_1	128.52(10)
C7_1–C8_1–C12_1	109.99(9)
C8_1–C9_1–C7_1	117.47(10)
C8_1–C9_1–H9_1	121.3
C7_1–C9_1–H9_1	121.3
C4_1–C10_1–C11_1	111.86(9)

C4_1–C10_1–C13_1	108.77(9)
C11_1–C10_1–C13_1	108.46(10)
C4_1–C10_1–C14_1	111.50(9)
C11_1–C10_1–C14_1	107.90(9)
C13_1–C10_1–C14_1	108.26(10)
C10_1–C11_1–H11A_1	109.5
C10_1–C11_1–H11B_1	109.5
H11A_1–C11_1–H11B_1	109.5
C10_1–C11_1–H11C_1	109.5
H11A_1–C11_1–H11C_1	109.5
H11B_1–C11_1–H11C_1	109.5
C8_1–C12_1–C6_1	102.95(9)

C8_1–C12_1–H12A_1	111.2
C6_1–C12_1–H12A_1	111.2
C8_1–C12_1–H12B_1	111.2
C6_1–C12_1–H12B_1	111.2
H12A_1–C12_1–H12B_1	109.1
C10_1–C13_1–H13A_1	109.5
C10_1–C13_1–H13B_1	109.5
H13A_1–C13_1–H13B_1	109.5
C10_1–C13_1–H13C_1	109.5
H13A_1–C13_1–H13C_1	109.5
H13B_1–C13_1–H13C_1	109.5
C10_1–C14_1–H14A_1	109.5

C10_1–C14_1–H14B_1	109.5
H14A_1–C14_1–H14B_1	109.5
C10_1–C14_1–H14C_1	109.5
H14A_1–C14_1–H14C_1	109.5
H14B_1–C14_1–H14C_1	109.5

Symmetry transformations used to generate equivalent atoms:

#1: 1-X, 1-Y, 1-Z;

Table S15. Torsion angles for 29.

**Atom–Atom–Atom–Atom**	**Torsion Angle [°]**
C6_1–C1_1–C2_1–C3_1	1.35(16)
C7_1–C1_1–C2_1–C3_1	−179.04(11)
C1_1–C2_1–C3_1–C4_1	−0.36(17)
C2_1–C3_1–C4_1–C5_1	−0.65(16)
C2_1–C3_1–C4_1–C10_1	178.11(10)
C3_1–C4_1–C5_1–C6_1	0.68(16)
C10_1–C4_1–C5_1–C6_1	−178.10(10)
C4_1–C5_1–C6_1–C1_1	0.29(16)
C4_1–C5_1–C6_1–C12_1#1	−179.68(11)
C2_1–C1_1–C6_1–C5_1	−1.33(16)
C7_1–C1_1–C6_1–C5_1	178.98(10)
C2_1–C1_1–C6_1–C12_1#1	178.65(10)
C7_1–C1_1–C6_1–C12_1#1	−1.04(12)
C2_1–C1_1–C7_1–C9_1	2.4(2)
C6_1–C1_1–C7_1–C9_1	−177.98(11)

C2_1–C1_1–C7_1–C8_1$^{\#1}$	−178.71(11)
C6_1–C1_1–C7_1–C8_1$^{\#1}$	0.93(12)
C7_1$^{\#1}$–C8_1–C9_1–C7_1	0.17(18)
C12_1–C8_1–C9_1–C7_1	178.26(11)
C8_1$^{\#1}$–C7_1–C9_1–C8_1	−0.17(18)
C1_1–C7_1–C9_1–C8_1	178.63(11)
C5_1–C4_1–C10_1–C11_1	−156.41(10)
C3_1–C4_1–C10_1–C11_1	24.86(14)
C5_1–C4_1–C10_1–C13_1	83.82(13)
C3_1–C4_1–C10_1–C13_1	−94.91(12)
C5_1–C4_1–C10_1–C14_1	−35.48(14)
C3_1–C4_1–C10_1–C14_1	145.78(11)

C9_1–C8_1–C12_1–C6_1$^{\#1}$	−178.11(11)
C7_1$^{\#1}$–C8_1–C12_1–C6_1$^{\#1}$	0.15(12)

Symmetry transformations used to generate equivalent atoms:

#1: 1-X, 1-Y, 1-Z;

References

[1] Broløs, L.; Kilde, M. D.; Brock-Nannestad, T.; Nielsen, M. B. *Eur. J. Org. Chem.* **2021**, *25*, 3537-3544.
[2] Bruker, *SAINT*, *V8.40A*, Bruker AXS Inc., Madison, Wisconsin, USA.
[3] Krause, L.; Herbst-Irmer, R.; Sheldrick, G. M.; Stalke, D. *J. Appl. Cryst.* **2015**, *48*, 3–10, doi:10.1107/S1600576714022985.
[4] Sheldrick, G. M. *Acta Cryst.* **2015**, *A71*, 3–8, doi:10.1107/S2053273314026370.
[5] Sheldrick, G. M. *Acta Cryst.* **2015**, *C71*, 3–8, doi:10.1107/S2053229614024218.
[6] Groom, C. R.; Bruno, I. J.; Lightfoot, M. P.; Ward, S. C. *Acta Cryst.* **2016**, *B72*, 171–179, doi:10.1107/S2052520616003954.
[7] Kratzert, D. *FinalCif*, *V125*, https://dkratzert.de/finalcif.html
